# Gene expression profiling in brain of mice exposed to the marine neurotoxin ciguatoxin reveals an acute anti-inflammatory, neuroprotective response

**DOI:** 10.1186/1471-2202-11-107

**Published:** 2010-08-26

**Authors:** James C Ryan, Jeanine S Morey, Marie-Yasmine Dechraoui Bottein, John S Ramsdell, Frances M Van Dolah

**Affiliations:** 1 Marine Biotoxins Program, NOAA Center for Coastal Environmental Health and Biomolecular Research, Charleston, SC, USA

## Abstract

**Background:**

Ciguatoxins (CTXs) are polyether marine neurotoxins and potent activators of voltage-gated sodium channels. This toxin is carried by multiple reef-fish species and human consumption of ciguatoxins can result in an explosive gastrointestinal/neurologic illness. This study characterizes the global transcriptional response in mouse brain to a symptomatic dose of the highly toxic Pacific ciguatoxin P-CTX-1 and additionally compares this data to transcriptional profiles from liver and whole blood examined previously. Adult male C57/BL6 mice were injected with 0.26 ng/g P-CTX-1 while controls received only vehicle. Animals were sacrificed at 1, 4 and 24 hrs and transcriptional profiling was performed on brain RNA with Agilent whole genome microarrays. RT-PCR was used to independently validate gene expression and the web tool DAVID was used to analyze gene ontology (GO) and molecular pathway enrichment of the gene expression data.

**Results:**

A pronounced 4°C hypothermic response was recorded in these mice, reaching a minimum at 1 hr and lasting for 8 hrs post toxin exposure. Ratio expression data were filtered by intensity, fold change and p-value, with the resulting data used for time course analysis, K-means clustering, ontology classification and KEGG pathway enrichment. Top GO hits for this gene set included acute phase response and mono-oxygenase activity. Molecular pathway analysis showed enrichment for complement/coagulation cascades and metabolism of xenobiotics. Many immediate early genes such as Fos, Jun and Early Growth Response isoforms were down-regulated although others associated with stress such as glucocorticoid responsive genes were up-regulated. Real time PCR confirmation was performed on 22 differentially expressed genes with a correlation of 0.9 (Spearman's Rho, p < 0.0001) with microarray results.

**Conclusions:**

Many of the genes differentially expressed in this study, in parallel with the hypothermia, figure prominently in protection against neuroinflammation. Pathologic activity of the complement/coagulation cascade has been shown in patients suffering from a chronic form of ciguatera poisoning and is of particular interest in this model. Anti-inflammatory processes were at work not only in the brain but were also seen in whole blood and liver of these animals, creating a systemic anti-inflammatory environment to protect against the initial cellular damage caused by the toxin.

## Background

Ciguatoxins (CTXs) are a suite of heat stable, lipid soluble, cyclic polyethers produced by benthic marine dinoflagellates of the genus *Gambierdiscus *[1]. These toxins activate voltage-gated sodium channels (VGSCs) [2], are bioaccumulated and metabolized to increasingly potent toxins through trophic transfer in reef associated fish [3], and are responsible for causing ciguatera fish poisoning (CFP) in humans, affecting an estimated 50,000 - 100,000 people each year [4]. CFP is characterized by acute gastrointestinal and neurological symptoms, including vomiting, diarrhea, abdominal pain, severe localized itching, tingling of extremities and lips, and thermal dysthesia. While gastrointestinal symptoms typically resolve within few days, other symptoms of CFP can last from several weeks to, in some cases, several years [5]. These long term symptoms can include fatigue, weakness, depression, as well as hypersensitivity to repeated exposure, and recurrence of symptoms that may occur upon consumption of non-toxic fish or alcohol [5].

**Figure 1:**
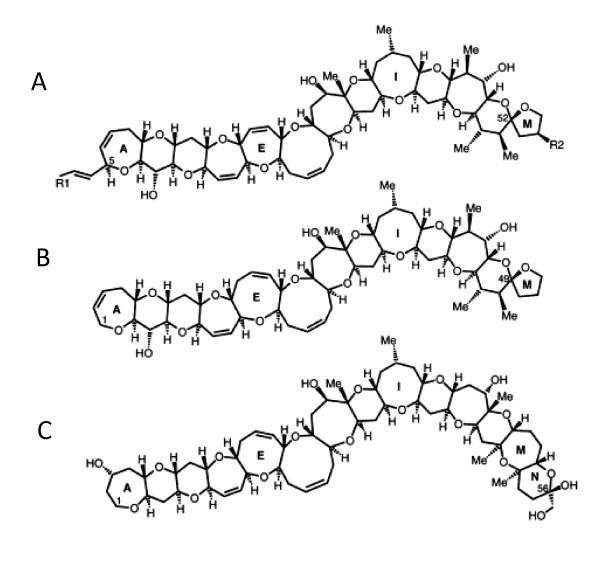
**Backbones of Pacific and Caribbean ciguatoxins**. A = P-CTX type 1, B = P-CTX type 2, C = C-CTX.

Suites of CTX congeners have been distinguished, with minor differences in their cyclic polyether backbone. As reviewed by Lewis [6], Pacific CTXs (P-CTX) have thirteen fused rings, with either a seven (Type 1) or eight (Type 2) member ring in the E position (Figure 1). CTX congeners are differentiated within each type by the hydroxyl groups at their A- or M-ring, and exclusively for the type-1, the four carbon saturated side chain extending from the A-ring. Differences in the R-groups of the type 1 P-CTX change the partition coefficient of the molecule by more than three orders of magnitude (NCBI PubChem Compound log P value of 2.5 for P-CTX-1 and 5.7 for P-CTX-4B). P-CTX-4B, the most lipophilic identified ciguatoxin, is a primary product of the algae while P-CTX-1, more polar, is a fish metabolite and 16-fold more potent than P-CTX-4B [7]. Other families of ciguatoxins have also been isolated from the Caribbean and Indian Ocean [8,9]. The former has fourteen fused rings; the later has not been structurally elucidated. In the Pacific Ocean, neurological symptoms dominate. Indian Ocean CFP is similar to the Pacific with the addition of hallucinogenic symptoms while gastrointestinal symptoms predominate in the Caribbean [10]. P-CTX-1 is the most potent known ciguatoxin and reported to cause human illness at 0.1 ppb [11]. It is a major congener found in carnivorous fish of the region and is thought to be a significant source of CFP in the Pacific [10].

The action of CTX isolated from a *Gambierdicus toxicus *culture, from the Martinique clone MQ2, was previously investigated in mice [12,13]. The toxin induced a rapid decrease in core body temperature that persisted for several hours with a corresponding induction of c-Fos mRNA in brain. Immunostaining for c-Fos-like immunoreactivity, showed positive immunoreactivity in only select brain regions including the medial preoptic and supraoptic nuclei of the hypothalamus and certain regions of the brain stem including the locus coeruleus, dorsolateral parabranchial nucleus, area postrema and the nucleus of the solitary tract. This study indicated that Caribbean ciguatoxin(s) produced by the algae had neuroexcitatory actions on several autonomic hypothalamic and brain stem regions, many of which appeared to be mediated by ascending projections.

Recent studies of acute CTX effects in mice have been conducted with the more potent and polar P-CTX-1 to examine toxicogenomics during a 24 hour period, which coincides with observable symptoms. P-CTX-1 exposure resulted in a rapid central response to lower body temperature and reduced motor activity, and a more persistent effect on spinal heat antinociception and delayed fever-like response [14]. Gene expression studies were conducted in parallel to characterize the immune response by examining whole blood, as well as potential detoxification pathways in the liver of these same animals. Through both proteomic and transcriptomic analysis of whole blood, there was evidence of an anti-inflammatory Th2 immune response [15], which is thought to be neuroprotective and may be advantageous to preventing neuronal damage during exposure to CTX. In the liver we identified differential expression of several genes involved in phase 1 and phase 2 detoxification pathways [16]. With such a high density of sodium channels in the brain, even limited ciguatoxin penetration makes this an interesting tissue to examine. The current study was designed to investigate responses of the brain to P-CTX-1 using oligonucleotide microarrays and real-time PCR. Gene ontology (GO) and Kyoto Encyclopedia of Genes and Genomes (KEGG) molecular pathway analysis were performed to identify possible enrichment of genes with specific biological themes.

## Results

### Mouse Symptomatic Responses

The animals used in this study were also analyzed in two companion papers looking at the transcriptomic response to CTX in blood and liver, for further details of symptoms see [15,16]. The treated mice all displayed hallmark symptoms of CTX exposure including hypothermia and hypoactivity while control animals displayed no obvious symptoms. All treated mice showed a rapid depression in core body temperature, which reached a minimum of 33°C at 1 h and slowly returned to basal temperature (37°C) by 8 hr.

### Microarray Analysis

Raw and processed gene expression data have been deposited in NCBI's Gene Expression Omnibus (GEO, http://www.ncbi.nlm.nih.gov/geo/, GEO Series accession number GSE20949). Triplicate Agilent whole mouse genome arrays from each time point (1, 4, and 24 h) underwent weighted averaging by Rosetta Resolver software to produce an average differential expression ratio for every gene at each time point. As different cutoffs for quality filtering for microarray data can provide different insights, we subjected our data to three different levels of stringency filters: high (1.7 fold change, p < 10^-5 ^and signal > 70 counts in both channels); medium (1.7 fold change, p < 10^-5^); and low (1.5 fold change, p < 0.0005) in at least one time point for a feature to be called significant, and then analyzed each data set individually. Of the 41,234 sequence probes on the array, totals of 550, 707, and 1,625 (Additional file 1), respectively, were found to be significantly differentially expressed. Although there are cases on the array where more than one probe is used to query different parts along the sequence of a single gene, and in some instances, identical probes to the same gene are replicated mainly for QC purposes, the vast majority of genes are queried using a single probe with the Agilent platform. Similar to earlier transcriptional profiling in blood and liver of the same CTX exposed animals, the majority of differential expression was seen at the 4 hr time point.

**Figure 2:**
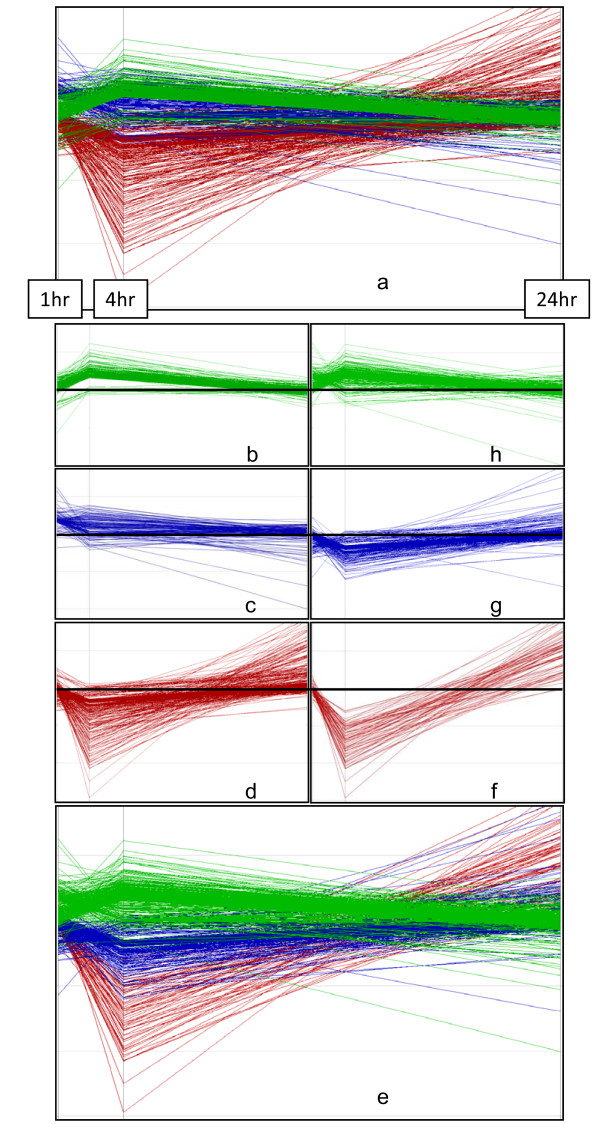
**Kmeans clustering of medium stringency microarray dataset**. 2a-d Pearson metric, 2e-h Euclidean metric. Each color represents the results of a discrete K-means cluster. Each line in the cluster represents the expression of a member gene over the time course. Bar across figures b-d and f-h indicates no change from control animal gene expression.

The use of different stringency filters, in this case, produced data sets with similar results throughout the analysis. For brevity, and more importantly a lack of appreciable differences, we did not present each set individually in all the figures and tables, but did present results from each level of quality filter. Clustering of the data resulting from each stringency filter was performed by K-means using 2 different metrics, Euclidian and Pearson, to identify genes with similar expression patterns (Figure 2). Membership in a cluster can provide insight into the activation of specific pathways, and crosstalk between pathways. The use of two different metrics for the K-means algorithm can identify different patterns of similarity, which is helpful for interpreting coordinated gene expression. The Pearson metric clusters genes with an emphasis on profiles of similar shape and direction while the Euclidian metric places an emphasis on absolute distance between expression values. Use of the different metrics produced slightly different cluster membership, as illustrated for the medium stringency filter data in Figure 2. This in turn produced slightly different ontology and pathway enrichment results as well (Table 1).

**Table 1:** Comparison of Gene Ontology and metabolic pathway enrichment by cluster metric (Euclidean vs Pearson) for medium stringency dataset.

*Pearson metric - Cluster 1 d - Biological Process*	Genes	% input	P-Value	Benjam
response to wounding	28	10.6	1.7E-15	9.2E-12
acute inflammatory response	17	6.4	2.6E-15	6.9E-12
response to external stimulus	32	12.1	5.6E-14	9.6E-11
inflammatory response	20	7.6	4.3E-11	5.6E-08
acute-phase response	9	3.4	7.8E-10	8.1E-07
response to stress	31	11.7	1.0E-07	9.0E-05
electron transport	22	8.3	2.4E-07	1.7E-04
complement activation	8	3	9.5E-07	6.2E-04
activ of plasma proteins in acute inflam response	8	3	9.5E-07	6.2E-04
complement activation, classical pathway	7	2.7	1.2E-06	6.0E-04
wound healing	10	3.8	1.8E-06	8.5E-04
generation of precursor metabolites and energy	23	8.7	2.3E-06	1.0E-03
humoral immune response by immunoglobulin	7	2.7	3.7E-06	1.5E-03
complement activation, alternative pathway	5	1.9	4.3E-06	1.6E-03
blood coagulation	8	3	8.1E-06	2.8E-03
coagulation	8	3	9.1E-06	2.9E-03
defense response	25	9.5	9.1E-06	2.8E-03
hemostasis	8	3	1.1E-05	3.2E-03
regulation of body fluid levels	8	3	5.0E-05	1.4E-02
activation of immune response	8	3	7.4E-05	1.9E-02
regulation of multicellular organismal process	14	5.3	1.1E-04	2.7E-02
regulation of immune response	9	3.4	1.4E-04	3.3E-02
immune response	19	7.2	1.5E-04	3.4E-02
regulation of immune system process	9	3.4	1.6E-04	3.3E-02
positive reg of multicellular organismal process	9	3.4	2.2E-04	4.4E-02
innate immune response	8	3	2.2E-04	4.4E-02
				
***Euclidian metric - Cluster 1g - Biological Process***	**Genes**	**% input**	**P-Value**	**Benjam**
acute inflammatory response	8	3.8	1.5E-05	7.5E-02
humoral immune response by immunoglobulin	6	2.8	2.1E-05	5.3E-02
response to external stimulus	17	8.1	2.7E-05	4.6E-02
***Euclidian metric - Cluster 1f - Biological Process***				
response to wounding	16	24.6	3.4E-14	1.7E-10
response to external stimulus	17	26.2	1.4E-12	3.6E-09
acute inflammatory response	9	13.8	1.8E-10	3.1E-07
acute-phase response	7	10.8	2.2E-10	2.9E-07
response to stress	17	26.2	3.4E-09	3.5E-06
wound healing	8	12.3	1.6E-08	1.4E-05
inflammatory response	10	15.4	3.6E-08	2.7E-05
blood coagulation	6	9.2	1.3E-06	8.5E-04
coagulation	6	9.2	1.4E-06	8.2E-04
hemostasis	6	9.2	1.7E-06	8.7E-04
regulation of body fluid levels	6	9.2	5.2E-06	2.4E-03
defense response	11	16.9	8.4E-05	3.6E-02

*Genes *heading indicates number of genes mapped to an ontology category. *% input *indicates the percentage of mapped genes from the total number of genes in the cluster. *P-Value *derived from Fishers exact test and *Benjam *indicates P-value after application of Benjamini multiple test correction.

### Gene Ontology and Pathway Analysis

GO (gene ontology) analysis using DAVID (Database for Annotation, Visualization of Integrated Discovery) [17] can identify over representation of GO categories, which can give insight into mechanisms responsive to ciguatoxin. The resultant gene data from each stringency filter was used as a whole but tended to produce non-specific, although significant, results such as the GO categories "metabolic process" and "binding". Analysis of individual K-means clusters was more informative. Overall, the described stringency and clustering metrics differed only slightly in resultant GO enrichment. Regardless of clustering metric or stringency, the genes of the up-regulated cluster (Figure 2b &2h) showed no significant enrichment for any GO category. The other clusters (Figure 2d, f, g), however, were more revealing. GO analysis of high, medium and low stringency filters, for both Euclidean and Pearson metrics, were similar, with only the medium stringency data shown (Table 1). As a result of the different metrics, some genes fell into different clusters producing different GO results, and no significant results occurred for cluster 1C, using the Pearson metric. When taken together, the most significant ontology classifications were for acute inflammatory response, response to external stimulus, and response to wounding, regardless of clustering metric or stringency filter.

KEGG pathway analysis of the 1,625 low stringency genes showed significant enrichment (p < 0.05) for three molecular pathways: complement/coagulation cascades, linoleic acid metabolism and metabolism of xenobiotics (Table 2). The last 2 pathways are dominated with cytochrome p450 enzymes, or Cyps. Cyps are reductases and oxidases critical to metabolism of xenobiotic compounds such as drugs and the toxin used here. Many of the Cyp isoforms found in brain in this study are also found in liver and are discussed in detail in the companion paper Morey et al, describing gene expression in the liver of these animals, the major site for metabolism of xenobiotics [16]. DAVID analysis of medium and high stringency data revealed enrichment of 5 pathways, including the 3 resulting from the low stringency set, as well as two new ones, arachidonic acid metabolism and γ hexachlorocyclohexane degradation, both Cyp rich. Analysis of individual clusters strengthened the significance (lower p-values) of these pathways, but did not add any new insights.

**Table 2:** Comparison of KEGG molecular pathway enrichment for high, medium and low stringency filtered genes.

*Low Stringency Set*	Genes	%input	P-Value	Benjam
Complement and coagulation cascades	26	1.3	1.3E-08	2.6E-06
Linoleic acid metabolism	15	0.7	1.3E-04	1.3E-02
Metabolism of xenobiotics by cytochrome P450	18	0.9	5.9E-04	3.8E-02
				
***Medium Stringency Set***	Genes	%input	P-Value	Benjam
Complement and coagulation cascades	19	3.1	7.4E-12	1.5E-09
Linoleic acid metabolism	15	2.5	1.1E-10	1.1E-08
Metabolism of xenobiotics by cytochrome P450	16	2.6	5.9E-09	3.8E-07
Arachidonic acid metabolism	10	1.7	6.2E-04	3.0E-02
gamma-Hexachlorocyclohexane degradation	6	1	9.1E-04	3.5E-02
				
***High Stingency Set***	Genes	%input	P-Value	Benjam
Linoleic acid metabolism	15	2.9	1.2E-11	2.3E-09
Complement and coagulation cascades	16	3.1	7.3E-10	7.1E-08
Metabolism of xenobiotics by cytochrome P450	15	2.9	6.0E-09	3.9E-07
Arachidonic acid metabolism	10	2	1.8E-04	8.9E-03
gamma-Hexachlorocyclohexane degradation	6	1.2	4.3E-04	1.7E-02

*Genes *heading indicates number of genes mapped to an ontology category. *% input *indicates the percentage of mapped genes from the total number of genes in the cluster. *P-Value *derived from Fishers exact test and *Benjam *indicates P-value after application of Benjamini multiple test correction.

The enriched molecular pathways and GO categories identified at all stringency levels for this data were predominantly driven by genes in the cluster down-regulated at 4 hrs and strongly up-regulated at 24 hrs (Figure 2d and 2f). The dramatic fold change seen here could be due in part to low expression levels for most of these genes, such that small changes in fluorescence intensity result in large fold-changes that may overestimate their responses. This is the main reason we added a minimum intensity criterion to our most stringent filter, minimizing false positives. In addition to genes involved in complement and coagulation pathways, and cytochrome p450 genes, there are several genes of the mouse major urinary protein family, or Mup, found in this down-regulated cluster. Mups are small ligand binding proteins best known for their role in excretion of pheromones. The differential expression of these genes is probably a result of chemosignalling induced by the stress of the experiment, with a relative lag in induction created by the hypothermia of treated animals. The binding pocket of Mups will accommodate a variety of small hydrophobic pheromone ligands, typically one quarter the size of ciguatoxin [18], but it would be interesting to determine if these proteins may be able to bind and excrete hydrophobic ciguatoxins.

### Comparison of brain, liver and blood gene expression

For each tissue, the low stringency conditions, (1.5 fold change, p < 0.0005) were used to filter for significant gene probes. This resulted in 17 probes that were differentially regulated and common to all tissues (Table 3). There were two instances of different, but similarly reporting, probes for the genes FK506 bp and Gag protein, reducing our unique gene total to 15. In general, these genes were regulated in the same direction for the three tissues assayed at 1 and 4 hrs. However, at 24 hrs, there was no statistically significant differential expression for these 15 genes in brain, only 2 significant changers in liver, and 1 in blood. When two of the three tissues had statistically significant differential gene expression at any time point, all three tissues were consistently regulated in the same direction as shown in Table 3, with two exceptions. C/EBP was significant at both 1 and 4 hours in all three tissues, although in brain and liver the gene was up-regulated at both time points, while in blood it was down-regulated at both time points. Arginase1 was significantly up-regulated in blood and liver but down in brain at 4 hrs.

**Table 3:** Significantly altered genes common to brain, liver and blood.

Accession #	Sequence Description	1 hr	4 hr
NM_019440	Immunity-related GTPase family M member 2 (Irgm2)		↑
NM_029000	GTPase, very large interferon inducible 1 (Gvin1)		↓
AF053745	Glycosylated gag protein (Mus dunni endogenous virus)		↑
NM_007705	Cold inducible RNA binding protein (Cirbp)		↑
NM_008245	Hematopoietically expressed homeobox (Hhex)	↓	
NM_026268	Dual specificity phosphatase 6 (Dusp6)		↓
NM_008361	Interleukin 1 beta (Il1b)		↓
NM_010220	FK506 binding protein 5 (Fkbp5)	↑	↑
BC057864	Polymeric immunoglobulin receptor 3 precursor (Pigr3)	↑	↑
NM_008330	Interferon gamma inducible protein		↓
NM_007679	CCAAT/enhancer binding protein (C/EBP), delta		
NM_010907	NFkB inhibitor, alpha (Nfkbia)	↑	↑
NM_016693	Mitogen-activated protein kinase kinase kinase 6 (Map3k6)		↑
BC021340	Poly (ADP-ribose) polymerase family, member 14 (Parp 14)		↓
NM_007482	Arginase 1 (Arg1)		

Arrows indicate where gene expression was in the same direction for all three tissues, when at least 2 tissues reported statistically significant data. Blank cells were largely due to the lack of significant data from more than 1 tissue at that particular time point.

### qPCR Validation

**Figure 3:**
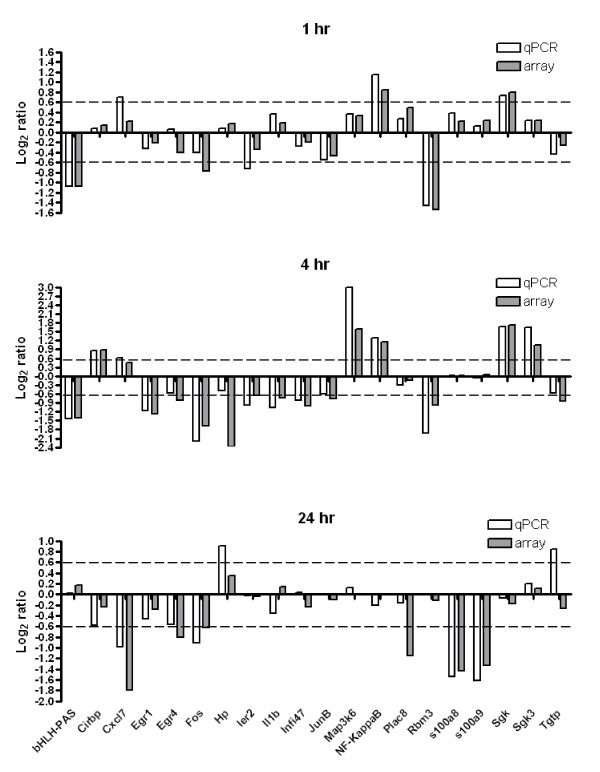
**Validation of microarray results by real-time PCR**. Dashed line indicates 1.5 fold change.

Twenty-two genes were selected for verification by real-time PCR, including transmembrane protein 59 used for normalization (Figure 3). Overall, the changes in gene expression measured by qPCR strongly supported the microarray results, with a correlation of 0.90 across the time series (Spearman's Rho, p < 0.0001, n = 66). Correlations at individual time points decreased throughout the time series with a maximum correlation of 0.91 (Spearman's Rho, p < 0.0001, n = 22) observed at 1 hr. Correlations decreased to 0.89 (Spearman's Rho, p < 0.0001, n = 22) or 0.65 (Spearman's Rho, p = 0.0011, n = 22) at the 4 and 24 hr time points, respectively. The fact that many of the genes validated exhibited very minor changes at the 24 hr time point is likely the cause of decreased correlation, as has been previously observed [19]. Overall, the direction of change reported by qPCR and microarray agreed for 59 of 66 samples and, with one exception, the observed fold change was less than 1.2 when the methods disagreed.

## Discussion

This study examines the genomic response in brain to a sub-lethal dose of the potent marine neurotoxin, P-CTX-1. The neurotoxic action of CTX is attributed to its activation of voltage-gated sodium channels (VGSCs) in peripheral and central nervous systems and cardiac, smooth and striated muscle, which collectively impact multiple organ systems [20]. Ciguatoxin is also associated with action on other systems, such as immune cells [21], not traditionally viewed as excitable. The brain provides a fascinating organ for investigation of ciguatoxin action because of its regional specialization, universal expression of voltage-gated sodium channels on neurons and certain glial cells, direct and humoral input from the periphery and a specialized immune system. Yet, other aspects of the brain provide complications to such analysis, such as regional differences in permeability to toxic agents and a strong hypothermic response to ciguatoxin in mice that is rarely reported in humans.

### Hypothermia/Immediate Early Response Genes/Neuroprotection

CTX-treated mice displayed a rapid 4°C decrease in core body temperature, which reached a minimum at 1 hr and slowly returned to baseline by 8 hr. Other marine polyether toxins, including maitotoxin and brevetoxin, cause similar acute stage hypothermia in mice [22,23] as do a variety of other xenobiotics including heavy metals, ethanol and organophosphates [24]. Many studies have shown hypothermia to positively impact recovery from traumatic injury and this hypothermic response appears to be a programmed protective mechanism against toxic insult. A recent review of beneficial effects of induced hypothermia after neurological injury cited four key elements for success: 1) speed of induction, with better outcomes in animals when cooling commences shortly after injury, 2) duration of cooling, which depends on degree of injury, 3) speed of rewarming, which should be slow, otherwise destructive processes will be reinitiated, and 4) management of side effects [25]. Animals in this study showed a rapid induction of hypothermia, with maximal cooling one hour after toxin exposure. Their return to normothermia took 7 hrs, relatively protracted compared to initiation. The protective mechanisms of hypothermia after brain trauma are well documented and include suppression of excitotoxicity, free radical production, intercellular signaling cascades, cerebral metabolism, neuroinflammation, blood brain barrier disruption, and seizure activity, as well as stabilizing membranes and cytoskeletal elements, in addition to modification of early gene expression [26].

Immediate early genes (IEGs) are a class of genes that quickly respond to a wide range of stimuli and also regulate a wide variety of functions. Primarily transcription factors, these genes such as Fos, Jun and Early Growth Response (EGR) isoforms can be found active in most acute gene expression studies. One study of hypothermia in rats proved to dramatically reduce the expression of IEGs after brain injury, but only to the level of control animals [27]. What is interesting in this study is that these genes, in particular, c-Fos, Jun B, Immediate early response 2 (Ier2), nuclear receptor 4A (Nr4a1), transcription factor NXF (NXF), Early growth response 1 (EGR1), EGR2, EGR4 are all significantly down-regulated at 1 and 4 hrs compared to controls. Many of these genes were independently validated by RT-PCR with similar results (Figure 3). In contrast, an earlier investigation of a Caribbean CTX isolated from algae, indicated a transient induction of c-Fos mRNA by northern analysis that paralleled a reduction of core body temperature [12]. In that study, immunohistochemistry for the Fos protein showed limited regional expression in hypothalamic and brain stem nuclei. It is possible that the Caribbean CTX used previously is less polar than P-CTX-1 used in the current study, where cFos is down-regulated, and therefore may have greater brain penetration. Comparable studies with the marine algal toxin domoic acid, a glutamate analog not known to cause hypothermia, produced acute up-regulation of these genes in the brain [28] and extensive expression of Fos and neuroexcitatory damage throughout the limbic system [28-31]. Domoic acid induced responses of immediate early genes parallel their rapid induction in brains of animals following hypoxia-ischemia, and are thought to play a role in the death cascade of sensitive neurons [32]. Another study using microarrays in a mouse seizure model found 6 genes specific to seizure that were up-regulated after one hour [33]. Three of these genes are significantly down-regulated in response to P-CTX at 1 and 4 hours, cFos, Egr1 and Nxf. A fourth, serum and glucocorticoid regulated kinase (Sgk) is up-regulated at 1 and 4 hrs while the remaining two were statistically unchanged in this study. Significantly, glucocorticoids up-regulate anti-inflammatory gene expression while also suppressing inflammatory gene expression [34]. Glucocorticoid regulation of inflammation is ubiquitous and is reflected by a gene found significantly up-regulated at 1 and 4 hrs in all tissue studied from these animals, FK506 binding protein 5. Fk506bp5 has been shown to inhibit binding of cortisol to the glucocorticoid receptor [35] and its increased expression suggests tight regulation of glucocorticoid signaling in the anti-inflammatory response of these animals. Additionally, CTX has been shown to act directly on adrenal chomafin cells and increase the release of catecholamines [36,37].

Another hallmark of anti-inflammatory response in the CTX treated mice is the down-regulation of IL1β gene expression in brain at 4 h (-1.7 fold), as was also seen in liver (-5.9 fold) and blood (-2.9 fold) [15,16]. IL1β is well known for its role in inflammation and the generation of fever, its down regulation, along with the down regulation of other inflammatory cytokines, may be contributing to the hypothermic response [38]. To further diminish IL1β signaling, the IL1 type 2 receptor is up-regulated, which is a decoy receptor that binds IL1β without any concomitant signaling, thereby sequestering the inflammatory cytokine [39]. This same regulation of Il1β was also seen in blood of these animals. Chemokine ligand 5, Ccl5, a chemotactic signal for eosinophil and T lymphocyte recruitment into inflamed areas was also down-regulated here. Another protein indicative of inflammation is the s100 protein heterodimer A8 and A9, calgranulin. Both subunits were found significantly down-regulated at 24 hrs. Additionally, just missing our fold change cut-off, cyclooxygenase II (COX2) was also down-regulated (-1.4 fold, p = .00004) in brain at 4 hrs. Hypothermia is quite effective at suppressing inflammation, and inflammatory genes that are down-regulated seem to correlate well with the temperature suppression seen here. Concurrently, many anti-inflammatory genes, such as those involved with glucocorticoid signaling, are up-regulated acutely.

### Coagulation and Complement

**Figure 4:**
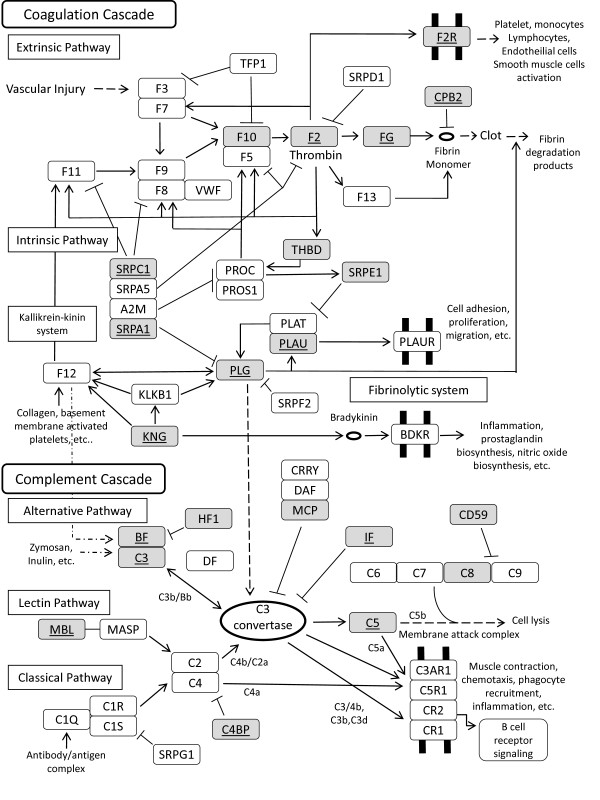
**Complement/Coagulation pathway activation**. Adaptation of KEGG Complement and Coagulation pathways. Shaded pathway members were found significant by low stringency filtering of data set while members underlined were found only in the high stringency results.

Molecular pathway analysis identified significant activity of complement and coagulation cascades. The complement system is a critical first line of defense against invading pathogens and also in the removal of cellular debris, such as necrotic tissue. The coagulation system is critical in maintaining hemostasis and together these two systems control many of the initial events in response to injury. Both systems employ cascades of serine proteases, which now have been shown to exhibit crosstalk at multiple levels, and together these systems significantly influence the magnitude and progression of inflammation in response to injury [40]. At all levels of data stringency, these pathways were found to undergo a high degree of regulation compared to control animals. Additionally, two poorly annotated genes in the high stringency data set, that were not mapped to the KEGG pathway (Figure 4), appear to have von Willebrand factor domains, a protein crucial to blood coagulation. The complement/coagulation pathway is of particular interest in light of recent findings in cases of chronic ciguatera illness in humans. These patients suffer from pathologic expression of activated complement component C4 as well as the essential clotting protein Factor VIII, ristocetin associated cofactor and von Willebrand's antigen itself [41]. The expression of these pathways is sharply down-regulated after acute CTX exposure in mice and coincides with the temperature suppression, which may be prophylactic against a potentially pronounced dysregulation.

Hypo-osmotic stress, due to sodium influx caused by ciguatoxin, is thought to cause neuronal swelling, which is why the osmolyte mannitol is thought to reduce some symptoms of exposure [42,43]. NFAT5, a transcription factor found to be up-regulated in response to hyper-osmotic stress [44] was down-regulated at 1 hr. Interestingly, recent studies have extended the direct actions of ciguatoxins beyond neurons and into blood cells. A study using frog erythrocytes showed that ciguatoxin caused swelling and actin cytoskeletal deformation in RBCs, through nitric oxide pathways [45]. The enzyme arginase 1 (Arg1) was one of only fifteen genes that was significantly regulated in liver, blood and brain of the P-CTX treated mice. Arg1 competes with inducible nitric oxide synthase (iNOS) for the L-arginine substrate, causing the reciprocal inhibition of these enzymes [46]. Expression of Arg1 is induced in macrophages predominantly by anti-inflammatory Th2 cytokines while iNOS is induced by inflammatory Th1 cytokines [47]. An *in vitro *study in macrophages showed ciguatoxin to stimulate an immune response, including a dramatic induction of iNOS [48]. Although Arg1 was sharply up-regulated in blood of the CTX treated mice, it was down-regulated in brain at 4 hrs.

### Comparison of Brain Gene Expression with Other Tissues

Fifteen genes were found to be similarly, and significantly, altered by ciguatoxin exposure in brain, liver and blood (Table 3). An interesting aspect of this comparison is the diversity of function between these tissues and the deficiency of target voltage gated sodium channels (VGSCs) in liver and leukocytes. The regulation of these genes is more likely driven by hypothermia and humoral input, rather than direct action of the toxin. In fact, many shared genes are readily identified as being immune related, such as immunity related GTPase, very large Ifn inducible GTPase, IL1β, Immunoglobulin receptor 3, Infγ inducible protein, NFκB inhibitor, and CEBPδ, which was formerly known as NF-IL6β. Cold inducible RNA binding protein (Cirbp) was up-regulated, peaking at 4 hrs in liver and brain, and then peaking in blood at 24 hrs. The differential expression of this gene is easily explained by the temperature suppression. Cirbp is a cold stress inducible protein that has been shown to protect cells from TNFα induced apoptosis [49]. Another sign of stress response in these animals is the common up regulation of the endogenous retroviral protein Gag. Gag can initiate innate immune activity through toll like receptors; TLR7, a receptor important to innate viral response, was found in the high stringency data, up-regulated more than 2-fold in brain. However, with the down regulation of genes involved with inflammation, the acute effects of TLR signaling are probably not fully realized in these animals.

## Conclusions

Although some of the genes in the brain induced by P-CTX-1 could indicate neuronal damage and dysfunction, these genes were not statistically over-represented, unlike the pathways discussed here. This is consistent with sensory, motor and autonomic actions of ciguatoxin that originate in the periphery. Instead, what was most apparent from this data was the suppression of inflammatory processes and activated humoral signaling. The hypothermic response coupled with down-regulation of complement/coagulation pathways and other inflammatory pathways could be involved in suppression of cerebral inflammation and edema during a critical time window after trauma. These processes were at work not only in the brain but were common to blood, liver and brain, creating a systemic anti-inflammatory environment to protect against the initial cellular damage caused by the toxin. Hypothermia is not a typical feature of human intoxications, but reports of temperature dysregulation are not unusual in ciguatera poisoning. Future work is needed to address questions raised in this study regarding potential differences in brain penetration and activation of thermoregulatory neurons by the different toxin congeners, as well as humoral contributions to temperature management.

## Methods

### Exposure to P-CTX-1

All exposures were conducted at Duke University in accordance with institutional and NIH guidelines for the ethical care and use of laboratory animals. Adult male C57/BL6 mice were maintained on a 12 hr:12 hr light:dark cycle and were given food and water *ad libitum*. Radio frequency transmitters were implanted in the mice to measure core temperature and motor activity as previously published [50]. Briefly, mice were anesthetized with ketamine HCl and a sedative analgesic, medetomidine HCl, and the transmitter (TA10TA-F40; Data Science International Sciences, St. Paul, MN) was implanted in the abdominal cavity. Following surgery, mice were administered atipamezol HCl by i.p. injection to counteract the anesthetic effect of ketamine. The mice were allowed at least 10 days of recovery before testing while animal health was monitored.

On the day of exposure, mice were weighed and randomly assigned to control or experimental groups. Three groups of control mice (n = 3) were injected i.p. with a single dose of physiological saline with 1% Tween 60 (vehicle). Three groups of experimental mice (n = 3) were injected ip with 0.26 ng/g P-CTX-1 in vehicle. P-CTX-1, obtained from Dr. Richard Lewis (University of Queensland, Australia), was purified from moray eel liver as described in Lewis *et al*. [51] with >90% purity. At 1, 4, and 24 hr post-injection, 3 experimental mice and 3 time-matched controls were anesthetized with 50 mg/ml sodium pentobarbital i.p. Brains were immediately dissected, flash frozen in liquid nitrogen, and stored at -80°C until RNA processing.

### RNA processing

The brains of the 3 control mice for each time point were pooled prior to RNA extraction while RNA was extracted from brains of individual CTX exposed mice. The brains were crushed using a BioPulverizer (BioSpec Products, Inc., Bartlesville, OK) in liquid nitrogen. The crushed tissue was immediately placed in cooled Tri-Reagent (Molecular Research Center, Inc., Cincinnati, OH) and homogenized using a Tissue-Tearor (United Lab Plastics, St. Louis, MO) at 25,000 rpm for 1 min on ice. All homogenates were processed according to the manufacturer's protocol. RNA was resuspended in nuclease-free water and further processed using an RNeasy mini-column (Qiagen, Valencia, CA) according to manufacturer's protocol. RNA was then quantified using a NanoDrop ND-1000 (Wilmington, DE) and qualified on an Agilent 2100 Bioanalyzer (Foster City, CA).

### RNA Labeling and Array Hybridization

Five hundred nanograms of total RNA from control and experimental animals was separately amplified and labeled with either Cy3 or Cy5 labeled CTP (GE Healthcare Life Sciences) using the Ambion Message Amp Amino Allyl kit according to manufacturer's protocol. Following labeling and clean up, amplified RNA and dye incorporation were quantified using a NanoDrop ND-1000. Seven hundred fifty ng each of Cy3 and Cy5 labeled targets were combined and hybridized to an Agilent catalog 44 K whole genome mouse oligonucleotide array for 17 h at 60°C. After hybridization, arrays were washed consecutively in solutions of 6X SSPE with 0.005% N-lauroylsarcosine and 0.06X SSPE with 0.005% N-lauroylsarcosine for 1 min each at room temperature. This was followed by a final 30 sec wash in Agilent Stabilization and Drying solution. Three biological replicates, including a dye swap, were performed at each time point.

### Microarray Analysis

The microarrays were run in a two color format where at each time point total brain RNA from 3 individual mice exposed to P-CTX-1 was compared to pooled total brain RNA from time-matched control mice (n = 3). Microarrays were imaged on an Agilent microarray scanner, extracted with Agilent Feature Extraction software version A8.5.3, and data analyzed with Rosetta Resolver 7.0 gene expression analysis system (Rosetta Informatics, Seattle, WA). Features were subjected to a combination linear and LOWESS normalization algorithm using a rank consistency filter. Resolver generated a weighted average composite array from the replicates (n = 3) for each time point based on the error model for the Agilent platform [52]. The composite arrays were used for a trend analysis to determine the expression pattern of genes throughout the time course. The data were subjected to three levels of stringency filters, high, medium and low, to sort out possible bias due to data quality. These filters consisted of: high (1.7 fold change) + (p < 10^-5^) + (signal > 70 counts in both channels); medium (1.7 fold change) + (p < 10^-5^); and low (1.5 fold change) + (p < 0.0005) in at least one time point. Data resulting from all 3 stringency filters were clustered using K-means with both Euclidean and Pearson metrics and further analyzed using the web tool DAVID (Database for Annotation, Visualization and Integrated Discovery) [17]. Gene ontology (GO) and KEGG molecular pathway analysis was performed to identify possible enrichment of genes with specific biological themes using both the data set as a whole and then in the individual K-means clusters. DAVID calculates a modified Fishers Exact p-value to demonstrate GO or molecular pathway enrichment, where p-values less than 0.05 after Benjamini multiple test correction are considered to be strongly enriched in the annotation category. For comparison between tissues, our low stringency filter (fc > 1.5, p < .0005) was applied to data from brain, liver and blood, to capture maximum overlap for analysis.

### Quantitative Real-Time PCR

Differentially expressed genes of interest were selected for validation of the microarray results by quantitative real-time PCR (qPCR). Triplicate reverse transcription reactions were carried out using 500 ng total RNA with an oligo(dT) primer using Ambion's RETROscript Kit (Austin, TX). Gene specific primers (Additional file 2) were used for qPCR on an ABI 7500 using the ABI Power SYBR Green master mix (Applied Biosystems, Foster City, CA). The optimal annealing temperature for each primer set was determined prior to the analysis of experimental samples. The specificity of each primer set and size of the amplicon were verified by analysis with Agilent's Bioanalyzer 2100 and further confirmed by melting curve analysis. The efficiency of each primer set was determined using a serial dilution series of cDNA from mouse brain. Duplicate 25 μl qPCR reactions were run from each cDNA triplicate. A cycle threshold (C_t_) was assigned at the beginning of the logarithmic phase of PCR amplification and the difference in the C_t _values of the control and experimental samples were used to determine the relative expression of the gene in each sample. Transmembrane protein 59 (NM_029565) was used for normalization as its expression did not change significantly in microarray or qPCR experiments (Wilcoxon, p > 0.05). As data were not normally distributed (Shapiro-Wilk W test), correlation to the microarray data set was determined by Spearman's Rho using JMP version 5.1.2 (SAS Institute, Cary, NC). Gene abbreviations; basic helix-loop-helix/Per-ARNT-Sim (bHLH-Pas), Cold inducible RNA-binding protein (Cirbp), Chemokine (C-X-C motif) ligand 7 (Cxcl7), Early growth response 1 (Egr1), Early growth response 4 (Egr4), c-fos (Fos), Haptoglobin (Hp), Immediate early response 2 (Ier2), Interleukin 1 beta (Il1b), Interferon inducible protein 47 (Infi47), Jun-b (JunB), MAP kinase kinase kinase 6 (Map3k6), Nuclear Factor kappa B (NF-KappaB), Placenta specific 8 (Plac8), RNA binding motif protein 3 (Rbm3), s100a8 (s100a8), s100a9 (s100a9), Serum and glucocorticoid regulated kinase (Sgk), Serum and glucocorticoid regulated kinase 3 (Sgk3), T-cell specific GTPase (Tgtp).

## Abbreviations

(CFP): Ciguatera Fish Poisoning; (CTX): Ciguatoxin, (P-CTX): Pacific Ciguatoxin, (DAVID): Database for Annotation, Visualization and Integrated Discovery; (GO): Gene Ontology; (Inf): Interferon; (Il1β): Interleukin 1 beta; (iNOS): Inducible nitric oxide synthase; (qPCR): Real time polymerase chain reaction; (Th): Helper T cell; (VGSC): Voltage gated sodium channel

## Competing interests

The authors declare that they have no competing interests.

## Authors' contributions

JCR performed the microarray studies and analysis, and drafted the manuscript. JSM performed the RT-PCR and helped edit the manuscript. MYBD participated in the animal dosing, tissue collection and editing of manuscript. JSR participated in the design of the study and editing of manuscript. FMV participated in the design of the study and editing of manuscript. All authors read and approved the final manuscript.

## NOAA Disclaimer

This publication does not constitute an endorsement of any commercial product or intend to be an opinion beyond scientific or other results obtained by the National Oceanic and Atmospheric Administration (NOAA). No reference shall be made to NOAA, or this publication furnished by NOAA, to any advertising or sales promotion which would indicate or imply that NOAA recommends or endorses any proprietary product mentioned herein, or which has as its purpose an interest to cause the advertised product to be used or purchased because of this publication.

## Supplementary Material

Additional file 1 **Processed and filtered microarray data**. The processed and filtered gene expression data used for analysis. Click here for file

Additional file 2 **PCR primers**. Accession numbers for genes along with PCR primers and annealing temperatures used for RT-PCR validation of microarray data. Click here for file

## References

[B1] Yasumoto T The chemistry and biological function of natural marine toxins Chem Rec 2001 1 3 228 242 10.1002/tcr.1010 11895121

[B2] Bidard JN Vijverberg HP Frelin C Chungue E Legrand AM Bagnis R Lazdunski M Ciguatoxin is a novel type of Na+ channel toxin J Biol Chem 1984 259 13 8353 8357 6330108

[B3] Legrand AM Fukui M Cruchet P Yasumoto T Progress on chemical knowledge of ciguatoxins Bull Soc Pathol Exot 1992 85 5 Pt 2 467 469 1340346

[B4] Begier EM Backer LC Weisman RS Hammond RM Fleming LE Blythe D Outbreak bias in illness reporting and case confirmation in ciguatera fish poisoning surveillance in south Florida Public Health Reports 2006 121 6 658 665 17278400 10.1177/003335490612100605 PMC1781907

[B5] Lehane L Lewis RJ Ciguatera: recent advances but the risk remains Int J Food Microbiol 2000 61 2-3 91 125 10.1016/S0168-1605(00)00382-2 11078162

[B6] Lewis RJ The changing face of ciguatera Toxicon 2001 39 1 97 106 10.1016/S0041-0101(00)00161-6 10936626

[B7] Murata M Legrand AM Ishibashi Y Fukui M Yasumoto T Structures and configurations of ciguatoxin from the moray eel Gymnothorax javanicus and its likely precursor from the dinoflagellate Gambierdiscus toxicus Journal of the American Chemical Society 1990 112 11 4380 4386 10.1021/ja00167a040

[B8] Hamilton B Hurbungs M Jones A Lewis RJ Multiple ciguatoxins present in Indian Ocean reef fish Toxicon 2002 40 9 1347 1353 10.1016/S0041-0101(02)00146-0 12220721

[B9] Pottier I Vernoux JP Jones A Lewis RJ Characterisation of multiple Caribbean ciguatoxins and congeners in individual specimens of horse-eye jack (Caranx latus) by high-performance liquid chromatography/mass spectrometry Toxicon 2002 40 7 929 939 10.1016/S0041-0101(02)00088-0 12076647

[B10] Lewis RJ Ciguatera: Australian perspectives on a global problem Toxicon 2006 48 7 799 809 10.1016/j.toxicon.2006.07.019 16930661

[B11] Lehane L Lewis RJ Ciguatera: recent advances but the risk remains International Journal of Food Microbiology 2000 61 2-3 91 125 10.1016/S0168-1605(00)00382-2 11078162

[B12] Peng YG Taylor TB Finch RE Moeller PD Ramsdell JS Neuroexcitatory actions of ciguatoxin on brain regions associated with thermoregulation Neuroreport 1995 6 2 305 309 10.1097/00001756-199501000-00020 7756616

[B13] Babinchak JA Moeller PDR Van Dolah FM Eyo PB Ramsdell JS Production of ciguatoxins in cultured Gambierdiscus toxicus Memoirs of the Queensland Museum Brisbane 1994 34 3 447 453

[B14] Bottein Dechraoui MY Rezvani AH Gordon CJ Levin ED Ramsdell JS Repeat exposure to ciguatoxin leads to enhanced and sustained thermoregulatory, pain threshold and motor activity responses in mice: relationship to blood ciguatoxin concentrations Toxicology 2008 246 1 55 62 10.1016/j.tox.2007.12.013 18280027

[B15] Ryan JC Bottein Dechraoui MY Morey JS Rezvani A Levin ED Gordon CJ Ramsdell JS Van Dolah FM Transcriptional profiling of whole blood and serum protein analysis of mice exposed to the neurotoxin Pacific Ciguatoxin-1 Neurotoxicology 2007 28 6 1099 1109 10.1016/j.neuro.2007.05.013 17868886

[B16] Morey JS Ryan JC Bottein Dechraoui MY Rezvani AH Levin ED Gordon CJ Ramsdell JS Van Dolah FM Liver genomic responses to ciguatoxin: evidence for activation of phase I and phase II detoxification pathways following an acute hypothermic response in mice Toxicological Sciences 2008 103 2 298 310 10.1093/toxsci/kfn055 18353800

[B17] Dennis G Sherman B Hosack D Yang J Gao W Lane HC Lempicki R DAVID: Database for Annotation, Visualization, and Integrated Discovery Genome Biology 2003 4 5 P3 10.1186/gb-2003-4-5-p3 12734009 PMC193660

[B18] Pertinhez TA Ferrari E Casali E Patel JA Spisni A Smith LJ The binding cavity of mouse major urinary protein is optimised for a variety of ligand binding modes Biochemical and Biophysical Research Communications 2009 390 4 1266 1271 10.1016/j.bbrc.2009.10.133 19878650

[B19] Morey J Ryan J Van Dolah F Microarray validation: factors influencing correlation between oligonucleotide microarrays and real-time PCR Biological Procedures Online 2006 8 1 175 193 10.1251/bpo126 17242735 PMC1779618

[B20] Dechraoui MY Wacksman JJ Ramsdell JS Species selective resistance of cardiac muscle voltage gated sodium channels: characterization of brevetoxin and ciguatoxin binding sites in rats and fish Toxicon 2006 48 6 702 712 10.1016/j.toxicon.2006.07.032 16973200

[B21] Ryan JC Bottein Dechraoui MY Morey JS Rezvani A Levin ED Gordon CJ Ramsdell JS Van Dolah FM Transcriptional profiling of whole blood and serum protein analysis of mice exposed to the neurotoxin Pacific Ciguatoxin-1 Neurotoxicology 2007 28 6 1099 1109 10.1016/j.neuro.2007.05.013 17868886

[B22] Gordon CJ Kimm-Brinson KL Padnos B Ramsdell JS Acute and delayed thermoregulatory response of mice exposed to brevetoxin Toxicon 2001 39 9 1367 1374 10.1016/S0041-0101(01)00092-7 11384725

[B23] Gordon CJ Yang Y Ramsdell JS Behavioral thermoregulatory response to maitotoxin in mice Toxicon 1998 36 10 1341 1347 10.1016/S0041-0101(98)00010-5 9723833

[B24] Gordon CJ Temperature Regulation in Laboratory Rodents 1993 New York: Press Syndicate of the University of Cambridge

[B25] Polderman KHMD Mechanisms of action, physiological effects, and complications of hypothermia Critical Care Medicine Therapeutic Temperature Management: State of the Art in the Critically Ill 2009 37 7 S186 S202 10.1097/CCM.0b013e3181aa5241 19535947

[B26] Truettner JS Suzuki T Dietrich WD The effect of therapeutic hypothermia on the expression of inflammatory response genes following moderate traumatic brain injury in the rat Molecular Brain Research 2005 138 2 124 134 10.1016/j.molbrainres.2005.04.006 15922484

[B27] Matsuda T Effects of hypothermia on c-fos and zif/268 gene expression following rat forebrain ischemia Journal of Anesthesia 1999 13 2 99 106 10.1007/s005400050034 14530948

[B28] Ryan JC Morey JS Ramsdell JS Van Dolah FM Acute phase gene expression in mice exposed to the marine neurotoxin domoic acid Neuroscience 2005 136 4 1121 1132 10.1016/j.neuroscience.2005.08.047 16216424

[B29] Colman JR Nowocin KJ Switzer RC Trusk TC Ramsdell JS Mapping and reconstruction of domoic acid-induced neurodegeneration in the mouse brain Neurotoxicol Teratol 2005 27 5 753 767 10.1016/j.ntt.2005.06.009 16109471

[B30] Peng YG Ramsdell JS Brain Fos induction is a sensitive biomarker for the lowest observed neuroexcitatory effects of domoic acid Fundam Appl Toxicol 1996 31 2 162 168 10.1006/faat.1996.0087 8789781

[B31] Peng YG Taylor TB Finch RE Switzer RC Ramsdell JS Neuroexcitatory and neurotoxic actions of the amnesic shellfish poison, domoic acid Neuroreport 1994 5 8 981 985 10.1097/00001756-199404000-00032 8061308

[B32] Dragunow M Beilharz E Sirimanne E Lawlor P Williams C Bravo R Gluckman P Immediate-early gene protein expression in neurons undergoing delayed death, but not necrosis, following hypoxic-ischaemic injury to the young rat brain Brain Research Molecular Brain Research 1994 25 1-2 19 33 10.1016/0169-328X(94)90274-7 7984048

[B33] Flood WD Moyer RW Tsykin A Sutherland GR Koblar SA <i>Nxf</i> and <i>Fbxo33</i>: novel seizure-responsive genes in mice European Journal of Neuroscience 2004 20 7 1819 1826 10.1111/j.1460-9568.2004.03646.x 15380003

[B34] Rhen T Cidlowski JA Antiinflammatory Action of Glucocorticoids -- New Mechanisms for Old Drugs N Engl J Med 2005 353 16 1711 1723 10.1056/NEJMra050541 16236742

[B35] Tatro ET Everall IP Kaul M Achim CL Modulation of glucocorticoid receptor nuclear translocation in neurons by immunophilins FKBP51 and FKBP52: Implications for major depressive disorder Brain Research 2009 1286 1 12 10.1016/j.brainres.2009.06.036 19545546 PMC2724600

[B36] Mattei C Wen PJ Nguyen-Huu TD Alvarez M Benoit E Bourdelais AJ Lewis RJ Baden DG Molgo J Meunier FA Brevenal inhibits pacific ciguatoxin-1B-induced neurosecretion from bovine chromaffin cells PLoS ONE 2008 3 10 e3448 10.1371/journal.pone.0003448 18941627 PMC2565126

[B37] Nguyen-Huu TD Mattei C Wen PJ Bourdelais AJ Lewis RJ Benoit E Baden DG Molgó J Meunier FA Ciguatoxin-induced catecholamine secretion in bovine chromaffin cells: Mechanism of action and reversible inhibition by brevenal Toxicon in press Corrected Proof 19682481 10.1016/j.toxicon.2009.08.002

[B38] Ren K Torres R Role of interleukin-1[beta] during pain and inflammation Brain Research Reviews 2009 60 1 57 64 10.1016/j.brainresrev.2008.12.020 19166877 PMC3076185

[B39] Mantovani A Locati M Polentarutti N Vecchi A Garlanda C Extracellular and intracellular decoys in the tuning of inflammatory cytokines and Toll-like receptors: the new entry TIR8/SIGIRR Journal of Leukocyte Biology 2004 75 5 738 742 10.1189/jlb.1003473 14673019

[B40] Amara U Rittirsch D Flierl M Bruckner U Klos A Gebhard F Lambris JD Huber-Lang M Interaction between the coagulation and complement system Adv Exp Med Biol 2008 632 71 79 19025115 10.1007/978-0-387-78952-1_6 PMC2713875

[B41] Shoemaker RC House D Ryan JC Defining the neurotoxin derived illness chronic ciguatera using markers of chronic systemic inflammatory disturbances: A case/control study. Neurotoxicology and Teratology 2010 10.1016/j.ntt.2010.05.007 20685390

[B42] Mattei C Molgo J Marquais M Vernoux J Benoit E Hyperosmolar D-mannitol reverses the increased membrane excitability and the nodal swelling caused by Caribbean ciguatoxin-1 in single frog myelinated axons Brain Research 1999 847 1 50 58 10.1016/S0006-8993(99)02032-6 10564735

[B43] Mattei C Dechraoui MY Molgo J Meunier FA Legrand AM Benoit E Neurotoxins targetting receptor site 5 of voltage-dependent sodium channels increase the nodal volume of myelinated axons Journal of Neuroscience Research 1999 55 6 666 673 10.1002/(SICI)1097-4547(19990315)55:6<666::AID-JNR2>3.0.CO;2-H 10220108

[B44] López-Rodríguez C Aramburu J Jin L Rakeman AS Michino M Rao A Bridging the NFAT and NF-[kappa]B Families: NFAT5 Dimerization Regulates Cytokine Gene Transcription in Response to Osmotic Stress Immunity 2001 15 1 47 58 10.1016/S1074-7613(01)00165-0 11485737

[B45] Sauviat MP Boydron-Le Garrec R Masson JB Lewis RL Vernoux JP Molgo J Laurent D Benoit E Mechanisms involved in the swelling of erythrocytes caused by Pacific and Caribbean ciguatoxins Blood Cells Molecules & Diseases 2006 36 1 1 9 10.1016/j.bcmd.2005.10.007 16364667

[B46] Modolell M Corraliza IM Link F Soler G Eichmann K Reciprocal regulation of the nitric oxide synthase/arginase balance in mouse bone marrow-derived macrophages by TH1 and TH2 cytokines Eur J Immunol 1995 25 4 1101 1104 10.1002/eji.1830250436 7537672

[B47] Munder M Eichmann K Modolell M Alternative Metabolic States in Murine Macrophages Reflected by the Nitric Oxide Synthase/Arginase Balance: Competitive Regulation by CD4+ T Cells Correlates with Th1/Th2 Phenotype J Immunol 1998 160 11 5347 5354 9605134

[B48] Matsui M Kumar-Roine S Darius HT Chinain M Laurent D Pauillac S Pacific ciguatoxin 1B-induced modulation of inflammatory mediators in a murine macrophage cell line Toxicon in press Corrected Proof 19520100 10.1016/j.toxicon.2009.05.039

[B49] Sakurai T Itoh K Higashitsuji H Nonoguchi K Liu Y Watanabe H Nakano T Fukumoto M Chiba T Fujita J Cirp protects against tumor necrosis factor-[alpha]-induced apoptosis via activation of extracellular signal-regulated kinase Biochimica et Biophysica Acta (BBA) - Molecular Cell Research 2006 1763 3 290 295 10.1016/j.bbamcr.2006.02.007 16569452

[B50] Gordon CJ Ramsdell JS Effects of marine algal toxins on thermoregulation in mice Neurotoxicology & Teratology 2005 27 5 727 731 10.1016/j.ntt.2005.06.012 16111859

[B51] Lewis RJ Sellin M Poli MA Norton RS MacLeod JK Sheil MM Purification and characterization of ciguatoxins from moray eel (Lycodontis javanicus, Muraenidae) Toxicon 1991 29 9 1115 1127 10.1016/0041-0101(91)90209-A 1665604

[B52] Weng L Dai H Zhan Y He Y Stepaniants SB Bassett DE Rosetta error model for gene expression analysis Bioinformatics 2006 22 9 1111 1121 10.1093/bioinformatics/btl045 16522673

