# Supplementary material for: Gene expression profiling in brain of mice exposed to the marine neurotoxin ciguatoxin reveals an acute anti-inflammatory, neuroprotective response
Source: BMC Neurosci. 2010 Aug 26;11:107. doi: 10.1186/1471-2202-11-107 (PMC2939656; doi:10.1186/1471-2202-11-107)
Supplement: Additional file 1 — Processed and filtered microarray data. The processed and filtered gene expression data used for analysis. [file 1471-2202-11-107-S1.PDF]

Additional file 1. Processed microarray data

		Time Point	1.0 Hours	1.0 Hours	4.0 Hours	4.0 Hours	24.0 Hours	24.0 Hours
Sequence Code	Sequence Name(s)	Sequence Description	P-value	Fold Change	P-value	Fold Change	P-value	Fold Change
A_51_P453329	4932416A15	hypothetical protein 4932416A15	5.24E-01	1.07	2.24E-18	1.60	2.71E-01	-1.09
A_51_P520809	2300010F08Rik,943008	Eph receptor A7, mRNA (cDNA clone MGC:14	8.07E-01	1.03	4.33E-09	2.26	9.26E-01	-1.02
A_52_P488814	Shprh,AA450458,AU02	Mus musculus 16 days embryo head cDNA, F	3.79E-02	1.14	7.10E-07	1.62	4.91E-01	1.07
A_52_P346256	2810422B04Rik,AU045	RIKEN cDNA 2810422B04 gene	7.06E-01	-1.03	8.00E-05	1.65	5.80E-04	-1.19
A_51_P503797	Lair1,Lair-1,mLair-1,BB1	DNA segment, Chr 7, Brigham & Women's Ge	9.99E-01	1.00	4.89E-09	-1.51	9.36E-01	1.00
A_52_P569602	D030004A10Rik	PREDICTED: similar to Snrpf protein [Mus mu	1.70E-08	1.53	1.00E+00	1.00	5.99E-01	1.28
A_51_P139334	Sorcs1,Sorcs	VPS10 domain receptor protein SORCS 1, mR	1.14E-07	1.84	8.40E-04	1.54	1.62E-01	1.15
A_52_P662013	Plg,Pg,AI649309	plasminogen	1.77E-01	-1.64	1.42E-08	-6.83	1.13E-03	6.61
A_52_P652289	AK032921	Mus musculus 12 days embryo male wolffian	1.53E-02	1.25	4.40E-04	1.70	1.94E-01	-1.04
A_52_P387458	1810035L17Rik	RIKEN cDNA 1810035L17 gene, mRNA (cDNA	3.33E-14	-2.21	1.30E-04	-1.87	9.16E-03	-1.22
A_51_P281134	Crsp2,ORF1,Gm641,Tra	Cofactor required for Sp1 transcriptional acti	4.41E-01	1.07	1.10E-04	1.72	9.81E-03	-1.22
A_52_P3530	D3Ertd751e,28100090	RIKEN cDNA 4930415G15 gene	4.31E-01	-1.06	4.93E-08	1.77	7.90E-03	-1.15
A_52_P175316	4921505C17Rik,AVO3,A	RIKEN cDNA 4921505C17 gene, mRNA (cDNA	2.00E-05	1.67	1.10E-02	2.19	7.70E-02	1.57
A_51_P219385	Six5,Dmahp,MDMAHP,	Sine oculis-related homeobox 5 homolog (Dr	1.03E-01	-1.06	6.16E-07	-1.65	9.39E-01	-1.00
A_51_P338443	Angptl4,ARP4,Bk89,FIA	angiopoietin-like 4	2.05E-03	1.48	0.00E+00	2.94	2.40E-04	1.33
A_52_P505907	Tnrc15,Gigyf2,AI85236	trinucleotide repeat containing 15	4.56E-01	-1.08	2.18E-10	-3.02	1.96E-01	1.10
A_51_P228574	Tat,MGC37772,MGC37	tyrosine aminotransferase	7.55E-01	-1.02	9.74E-31	-2.27	2.41E-01	1.43
A_52_P550912	Cdc2l5,2310015O17Rik	Cell division cycle 2-like 5 (cholinesterase-rel	2.31E-01	1.19	8.75E-06	1.55	5.51E-01	1.04
A_52_P638439	Ppp1r2,IPP-2,D16Ertd2	protein phosphatase 1, regulatory (inhibitor)	4.54E-01	1.04	7.66E-06	1.54	1.92E-01	-1.04
A_51_P467349	Trim62,AI450348,6330	Tripartite motif-containing 62 (Trim62), mRN	6.77E-01	-1.04	1.70E-04	1.54	9.42E-01	1.02
A_52_P1076447	AK087404	0 day neonate eyeball cDNA, RIKEN full-lengt	1.16E-12	1.75	4.53E-01	-1.76	5.88E-01	1.24
A_52_P124083	A530082C11Rik,SLC35E	RIKEN cDNA A530082C11 gene, mRNA (cDNA	2.60E-04	-1.38	1.90E-04	-1.56	9.94E-01	-1.00
A_51_P418725	Plekhf1,APPD,PHAFIN1,	RIKEN cDNA 1810013P09 gene	1.05E-01	1.11	5.14E-11	1.61	3.30E-01	-1.12
A_52_P350214	TC1064783	Unknown	1.77E-03	1.13	3.90E-04	-1.73	1.67E-01	1.13
A_51_P156309	AK047917	Mus musculus 16 days embryo head cDNA, F	1.80E-04	1.67	8.47E-01	1.07	1.66E-01	1.52
A_52_P80944	Zfp36,Ttp,Gos24,TISII,N	zinc finger protein 36	7.59E-11	1.46	4.50E-04	1.81	1.16E-03	1.28
A_52_P980022	Mmp16,Mt3mmp,MT3	Matrix metallopeptidase 16 (Mmp16), mRNA	1.02E-01	-1.27	2.93E-01	-1.74	4.11E-06	1.31
A_52_P464372	P2ry1,P2Y1	purinergic receptor P2Y, G-protein coupled 1	2.15E-02	-1.14	7.00E-05	1.61	1.24E-02	-1.19
A_51_P335519	Slc22a12,Rst,OAT4L,UR	solute carrier family 22 (organic cation trans	2.79E-01	-1.36	3.95E-06	-1.57	4.08E-01	1.44
A_52_P107717	Exoc4,Sec8,C78892,Sec	SEC8 (S. cerevisiae), mRNA (cDNA clone MGC	1.64E-01	1.23	2.30E-04	2.36	7.79E-01	1.02
A_52_P956057	Fbxl11,Fbl7,Cxxc8,Fbl11	MKIAA1004 protein	1.19E-01	-1.32	1.78E-09	-1.83	4.00E-01	1.12
A_52_P4482	NAP108144-1	Unknown	3.24E-01	1.06	1.67E-06	1.76	3.56E-01	-1.07
A_51_P245503	Ugt2b1,1300012D20Rik	RIKEN cDNA 1300012D20 gene	1.00E+00	1.00	1.34E-06	-4.71	3.00E-05	24.41
A_52_P185343	Gna13,AU024132,AU04	Guanine nucleotide binding protein, alpha 13	5.00E-01	1.06	2.27E-30	1.62	1.23E-01	-1.08
A_51_P453909	Cyp2f2,Cyp2f	cytochrome P450, family 2, subfamily f, poly	2.71E-18	-1.49	3.32E-10	-1.99	2.47E-01	1.15
A_51_P303749	Depdc1b,XTP1,AW2604	expressed sequence AW260467	3.30E-01	-1.10	3.45E-02	-1.62	5.00E-05	-1.33
A_52_P795611	Aldh1l2,MGC19039,D3	Aldehyde dehydrogenase 1 family, member L	2.34E-07	1.64	1.35E-03	1.25	8.37E-02	1.26
A_51_P437628	Uncx4.1,Chx4	Unc4.1 homeobox (C. elegans)	1.30E-27	-1.63	9.49E-08	1.36	5.78E-01	-1.06
A_51_P388661	Msx2,Hox8,Hox-8,Hox8	homeo box, msh-like 2	0.00E+00	-1.54	3.22E-06	-1.63	8.74E-01	-1.01
A_51_P341821	Arid4b,BCAA,BRCAA1,R	Mus musculus 4 days neonate male adipose	4.10E-04	1.59	2.07E-01	-1.52	7.74E-02	1.22
A_52_P275324	C79248	RNA-binding region (RNP1, RRM) containing	7.97E-03	1.15	3.70E-04	1.50	9.98E-01	-1.00
A_51_P155257	B4galt1,GalT,Ggtb,Ggtb	UDP-Gal:betaGlcNAc beta 1,4- galactosyltran	4.93E-01	1.05	9.17E-08	1.51	7.22E-01	-1.03
A_52_P166868	Nudt13,4933433B15Rik	Nudix (nucleoside diphosphate linked moiety	1.34E-01	1.13	2.00E-05	1.63	1.83E-01	-1.10
A_51_P176154	Sirpb1,Sirpb,SIRP-beta,	Signal-regulatory protein beta 1 (Sirpb1), tran	7.14E-06	2.13	7.06E-02	-1.87	2.16E-01	-1.19
A_52_P139316	NAP112201-1	Unknown	8.51E-01	-1.02	3.51E-14	-10.57	1.39E-02	2.88
A_52_P827306	Dscr1l1,MCIP2,ZAKI-4	Calcineurin inhibitory protein ZAKI-4	3.18E-08	1.78	1.00E+00	1.00	3.41E-01	1.24
A_52_P747659	AK086345	Mus musculus 15 days embryo head cDNA, F	2.70E-04	1.63	6.72E-01	1.17	4.95E-01	-1.30
A_51_P446022	D430042O09Rik,MGC3	RIKEN cDNA D430042O09 gene, mRNA (cDN	1.11E-01	1.15	2.28E-07	1.68	9.99E-01	-1.00
A_52_P1188614	C230079D11Rik,Gm329	PREDICTED: RIKEN cDNA C230079D11 gene		5.91E-08	1.69	6.72E-01	1.10	1.46E-01	1.62
A_52_P64383	Klf15,CKLF,KKLF,AV048	Kruppel-like factor 15	3.00E-05	1.26	9.66E-18	2.17	9.22E-01	-1.01
A_52_P156273	4732496G21Rik,AI4483	RIKEN cDNA 4732496G21 gene (4732496G2	5.64E-06	1.24	5.11E-07	1.51	2.10E-01	1.07
A_51_P384314	B930007L02Rik,AI4474	RIKEN cDNA B930007L02 gene, mRNA (cDNA	7.48E-28	3.38	8.28E-02	2.65	5.35E-01	1.31
A_52_P83233	Xpa,Xpac,AI573865	xeroderma pigmentosum, complementation	2.00E-02	1.18	3.11E-17	1.54	9.79E-03	-1.09
A_51_P386503	1300007F04Rik	RIKEN cDNA 1300007F04 gene	2.60E-16	1.42	3.16E-10	2.26	1.53E-02	1.14
A_51_P179894	AK090370	NOD-derived CD11c +ve dendritic cells cDNA	5.40E-04	1.67	6.75E-10	2.35	1.88E-01	-1.14
A_52_P1132041	D130067I03Rik,B93008	RIKEN cDNA D130067I03 gene, mRNA (cDNA	4.00E-05	1.63	2.86E-01	1.25	2.85E-01	1.37
A_52_P996506	Nlgn1,BB179718,MGC1	Neuroligin 1 (Nlgn1), mRNA	1.40E-04	1.65	1.00E+00	1.00	1.00E+00	1.00
A_52_P188425	Prosc,1700024N20Rik,	Proline synthetase co-transcribed (Prosc), m	2.84E-01	1.11	1.87E-10	1.61	4.22E-01	-1.05
A_52_P374897	Arg2,AII,AU022422	arginase type II	2.84E-01	1.12	8.40E-17	1.57	6.70E-03	-1.15
A_52_P470030	Ankrd15,AU015049,AW	Ankyrin repeat domain 15, mRNA (cDNA clon	9.85E-01	1.00	4.40E-04	-3.62	5.40E-02	1.12
A_52_P1163884	Ina,NF-66,AV028420,M	Internexin neuronal intermediate filament pr	8.50E-09	1.30	1.70E-04	2.32	6.55E-01	1.05
A_51_P288876	Tmem45a,p19.5,M324	RIKEN cDNA C630002M10 gene	3.83E-01	-1.05	8.60E-08	-1.52	1.42E-01	-1.10
A_51_P486923	Ibsp,BSP	integrin binding sialoprotein	7.29E-01	-1.13	2.45E-01	1.80	1.53E-14	-4.34
A_52_P1107500	E130118H10Rik	0 day neonate eyeball cDNA, RIKEN full-lengt	1.07E-17	2.22	1.42E-13	2.08	6.00E-05	1.29
A_51_P361448	Scara5,AV278087,4932	RIKEN cDNA 4933425F03 gene	5.58E-02	-1.14	1.40E-04	1.51	4.58E-01	-1.07
A_52_P399998	Atp6v0a1,Vpp1,Vpp-1,	ATPase, H+ transporting, lysosomal V0 subur	1.65E-01	1.15	2.06E-12	1.80	4.95E-02	-1.22
A_52_P246255	Cyp2a5,Coh,Cyp15a2	cytochrome P450, family 2, subfamily a, poly	7.90E-01	1.08	1.30E-04	-3.07	5.60E-02	3.49
A_51_P238722	C1qr1,Ly68,AA4.1,C1qr	complement component 1, q subcomponent	6.92E-01	1.02	4.11E-17	-1.63	1.76E-01	-1.08
A_52_P939146	AK034770	12 days embryo embryonic body between di	7.45E-10	-2.83	1.63E-03	-2.36	2.00E-01	-1.18
A_52_P190506	Mrpl15,Rpml7,MRP-L7,	Mus musculus 8 days embryo whole body cD	2.72E-07	1.72	9.70E-18	2.94	9.87E-02	-1.14
A_51_P217878	Mtap1b,LC1,MAP5,MA	Microtubule-associated protein 1 B (Mtap1b	1.35E-06	1.30	1.10E-03	-1.67	2.27E-01	1.25
A_52_P29646	2810028N01Rik	Mus musculus RIKEN cDNA 2810028N01 gen	9.82E-01	-1.00	2.11E-09	1.66	2.53E-03	-1.20
A_51_P366138	Mertk,Eyk,Mer,Nyk	c-mer proto-oncogene tyrosine kinase	4.05E-01	1.05	2.39E-21	1.54	1.86E-01	1.06

A_52_P174840	Lrrcc1,AI195358,AI4474	RIKEN cDNA 1200008A14 gene	6.99E-01	1.03	2.35E-06	1.54	1.05E-03	-1.15
A_51_P241645	BC030440,MGC40914	cDNA sequence BC030440	5.85E-01	-1.07	6.55E-06	1.55	1.29E-01	-1.17
A_52_P675857	LOC233184	Mus musculus similar to Apopolysialoglycopr	8.59E-01	-1.01	1.60E-07	-1.70	5.10E-01	1.04
A_51_P496187	N28178,mKIAA1045,A2	expressed sequence N28178	3.98E-01	-1.43	6.00E-05	-1.55	4.50E-01	1.45
A_51_P402868	Pla2g3,9130003P18Rik	Phospholipase A2, group III, mRNA (cDNA clo	3.49E-01	1.07	1.13E-10	1.61	9.53E-02	1.31
A_51_P237856	Ifi203	interferon activated gene 203	5.11E-01	-1.07	1.24E-11	-1.62	4.38E-01	-1.07
A_52_P609648	E130120C16Rik	Mus musculus 0 day neonate eyeball cDNA, I	3.67E-20	1.51	2.66E-01	-1.09	6.50E-01	-1.03
A_51_P197213	Pnpla2,TTS-2.2,061003	RIKEN cDNA 0610039C21 gene	2.65E-02	1.10	1.91E-30	2.10	2.21E-01	1.08
A_52_P131423	Mbd1,PCM1,Cxxc3	Methyl-CpG binding domain protein 1 (Mbd1	7.79E-14	1.62	1.00E-05	1.72	3.45E-01	1.07
A_52_P684066	1110014L15Rik	18-day embryo whole body cDNA, RIKEN full	3.61E-03	-1.11	1.50E-04	2.05	7.58E-01	1.04
A_51_P161401	A130096K20	hypothetical protein A130096K20	4.31E-07	1.56	9.01E-01	1.07	2.06E-01	1.27
A_51_P187253	Irf8,Myls,ICSBP,IRF-8,Ic	interferon consensus sequence binding prote	1.40E-04	-1.23	4.22E-07	-1.61	6.03E-02	1.07
A_52_P557212	Adamts10	a disintegrin-like and metalloprotease (repro	2.60E-20	1.90	9.53E-01	1.02	8.69E-01	-1.02
A_52_P398941	9630011N22Rik	RIKEN cDNA 9630011N22 gene	1.70E-06	2.27	1.00E+00	1.00	2.18E-01	1.56
A_52_P25357	NAP027049-1	Unknown	1.07E-01	-1.16	9.19E-14	-1.58	1.48E-02	-1.39
A_52_P474528	NAP066348-1	Unknown	1.93E-10	1.85	3.78E-08	1.97	2.46E-01	1.08
A_52_P176026	Xlr4b,Xlr4	X-linked lymphocyte-regulated 4	3.82E-07	-2.11	7.81E-10	6.37	7.29E-01	1.16
A_51_P416295	Iigp2,GTPI,AI481100,M	expressed sequence AI481100	6.00E-05	-1.42	3.59E-09	-1.66	4.46E-01	-1.04
A_51_P167426	Herc1,MGC7618,B2302	Hect (homologous to the E6-AP (UBE3A) carb	5.02E-11	1.51	4.00E-05	2.33	6.25E-01	-1.04
A_52_P795289	Klhl7,Klhl6,SBBI26,D5Er	Kelch-like 7 (Drosophila) (Klhl7), mRNA	2.81E-06	2.38	1.00E+00	1.00	1.00E+00	1.00
A_52_P681557	2810043G22Rik	Mus musculus hypothetical LOC229146 (LOC	4.35E-02	-1.23	3.49E-06	1.86	2.12E-01	-1.15
A_51_P336325	Orm1,Agp-1,Agp-2,Orm	orosomucoid 1	5.67E-01	-1.36	0.00E+00	-12.49	2.85E-08	9.18
A_52_P1050743	1110035E04Rik	Nuclear factor of activated T-cells 5 (Nfat5)	6.45E-26	-1.81	3.11E-02	-1.18	4.60E-01	-1.13
A_51_P497937	Gja12,Cx47,B230382L1	gap junction membrane channel protein alph	1.10E-02	1.16	1.00E-05	-1.51	4.00E-05	-1.35
A_52_P661713	Gvin1,VLIG,Iigs1,VLIG-1	RIKEN cDNA 9130002C22 gene	1.89E-01	-1.06	1.22E-13	-1.94	7.32E-02	-1.25
A_51_P205385	Uox,AI663847	urate oxidase	5.81E-01	1.06	8.65E-12	-1.81	1.72E-01	1.90
A_51_P136014	Tfdp2,DP3,DP-3,A3300	DP-3=protein regulating cell cycle transcripti	5.39E-01	1.04	8.88E-18	1.63	7.12E-01	-1.01
A_52_P475578	B130020M22Rik	Mus musculus 16 days embryo head cDNA, F	2.80E-45	1.78	1.00E+00	1.00	4.18E-01	1.26
A_52_P1180340	Cadps,CAPS,CAPS1,AU0	Ca<2+>dependent activator protein for secre	1.37E-08	1.56	3.25E-01	1.09	3.60E-01	-1.06
A_52_P1367	TC1025241	U96416 cytochrome b {Dennyus distinctus ti	1.71E-14	-1.52	7.39E-02	-1.38	3.99E-01	-1.09
A_51_P513504	Gosr1,GS28,GOSRI,GOS	golgi SNAP receptor complex member 1	9.40E-02	1.15	4.40E-04	1.50	6.16E-03	-1.17
A_52_P448357	2610102M01Rik	RIKEN cDNA 2610102M01 gene	9.45E-03	1.15	1.00E-05	1.73	7.00E-05	-1.41
A_52_P1115713	Odz2,Odz3,Ten-m2,mK	ODZ2 (Odz2)	4.04E-08	1.65	5.58E-01	-1.07	2.85E-01	1.24
A_52_P572099	Plekha1,TAPP1,AA9605	Pleckstrin homology domain containing, fam	6.43E-36	1.55	1.61E-01	1.28	6.73E-01	1.07
A_51_P363862	Sdfr1,AW554172	Stromal cell derived factor receptor 1 (Sdfr1)	2.88E-07	1.56	1.00E+00	1.00	2.85E-03	1.39
A_52_P353068	ENSMUST0000006842C	Unknown	3.10E-04	-1.22	1.19E-01	-1.94	1.67E-02	1.35
A_51_P296328	Ahcyl1,DCAL,Irbit,AA40	S-adenosylhomocysteine hydrolase-like 1	2.92E-01	1.06	1.10E-04	1.54	4.07E-01	-1.03
A_51_P254895	BC013476,MGC18880	Mus musculus similar to cytochrome P450 4/	8.91E-01	-1.02	1.00E-28	-2.92	2.84E-02	1.43
A_52_P855955	BI697602	Transcribed locus	4.56E-02	-1.06	1.26E-22	1.51	1.08E-01	1.06
A_52_P482063	1500010G04Rik	RIKEN cDNA 1500010G04 gene	7.00E-05	-1.51	1.16E-03	-1.60	6.83E-01	1.03
A_52_P411430	Ctcf,AW108038	PREDICTED: Mus musculus hypothetical prot	3.78E-02	1.24	9.07E-15	1.63	5.62E-01	1.02
A_52_P851306	AK044145	Mus musculus 10 days neonate cortex cDNA,	2.00E-05	1.72	1.00E+00	1.00	8.21E-01	1.07
A_51_P132037	Lrrtm1,AW125451,463	Leucine rich repeat transmembrane neurona	3.08E-06	1.76	2.40E-02	1.59	6.58E-01	1.11
A_52_P24365	Lrrc44,BB020367,49304	RIKEN cDNA 4933403H06 gene	4.00E-05	-1.45	3.35E-01	1.50	8.24E-01	-1.05
A_51_P335413	2900027M19Rik	Transmembrane protein 28 (Tmem28), mRN	1.24E-08	1.69	5.36E-01	-1.18	9.38E-01	1.01
A_52_P673307	2610044O15Rik	RIKEN cDNA 2610044O15 gene	3.43E-02	1.11	1.03E-09	1.51	4.82E-03	-1.17
A_52_P232508	Mup3,MUP15,Mup-3,M	Mus musculus major urinary protein 3 (Mup3	8.78E-01	-1.03	2.40E-26	-30.96	6.17E-02	4.29
A_52_P49797	TC982387	AF053745 glycosylated gag protein {Mus dur	4.45E-16	2.07	6.29E-13	2.36	7.66E-01	-1.01
A_52_P35895	Akap2,AKAP-KL,AA9597	A kinase (PRKA) anchor protein 2, mRNA (cD	9.88E-01	-1.00	4.72E-06	1.52	9.01E-01	-1.01
A_52_P583399	ENSMUST00000048525	Unknown	9.37E-01	-1.00	1.50E-04	1.52	7.32E-01	-1.04
A_51_P458722	LOC432600	similar to keratin associated protein 9-1	4.66E-01	1.13	1.63E-10	2.25	5.95E-01	1.09
A_52_P1132271	Ube2e1,UbcM3,Ubce5,	Ubiquitin-conjugating enzyme E2E 1, UBC4/5	3.80E-01	1.06	1.00E-04	1.59	2.08E-01	1.15
A_52_P303841	LOC384261	hypothetical LOC384261	3.20E-07	1.18	9.00E-03	1.52	4.18E-01	-1.05
A_51_P212420	Lama4	Mus musculus laminin, alpha 4 (Lama4), mRN	1.35E-01	1.13	2.15E-41	-1.72	7.43E-01	1.02
A_51_P379552	3110047P20Rik,Hn1-ps	RIKEN cDNA 3110047P20 gene, mRNA (cDNA	9.43E-01	1.00	4.19E-19	-1.84	1.06E-03	1.16
A_52_P354974	Syt11,1500004A13Rik,3	RIKEN cDNA 5830417I10 gene, mRNA (cDNA	4.58E-02	1.24	5.08E-14	1.65	2.34E-01	-1.05
A_51_P270577	Rnf168,3110001H15Rik	RIKEN cDNA 3110001H15 gene	1.64E-01	-1.07	1.36E-08	1.79	5.93E-01	-1.05
A_52_P1116282	Odz3,Odz1,Ten-m3,mK	ODZ3 (Odz3)	1.10E-08	2.43	8.49E-01	1.08	2.66E-02	2.37
A_52_P523399	Six6os1,Six6OS,492150	RIKEN cDNA 4930447C04 gene	8.73E-01	-1.02	1.90E-04	1.62	9.75E-02	-1.15
A_52_P1028683	C230047L17Rik	PREDICTED: similar to cajalin 2 isoform a [Mu	3.00E-05	1.80	5.10E-01	1.41	9.40E-02	1.39
A_52_P762590	5830420C07Rik	F-box and WD-40 domain protein 11 (Fbxw1	1.00E-04	1.42	1.00E-04	1.78	2.47E-01	1.79
A_51_P292823	4930594C11Rik	Mannoside acetylglucosaminyltransferase 4,	3.55E-02	-1.18	1.64E-06	-2.23	2.29E-01	1.13
A_51_P239654	Nr4a1,Hmr,N10,TR3,Gf	nuclear receptor subfamily 4, group A, meml	6.54E-18	-1.50	9.69E-08	-1.83	6.66E-02	-1.35
A_51_P196695	Il7r,CD127,MGC107557	interleukin 7 receptor	1.07E-01	1.35	1.80E-21	4.64	1.00E+00	1.00
A_52_P282631	Prpf39,Srcs1,MGC3707	PRP39 pre-mRNA processing factor 39 homo	9.50E-11	1.70	7.89E-01	1.11	2.53E-01	1.77
A_51_P272106	Cirbp,Cirp,R74941	cold inducible RNA binding protein	5.09E-01	1.10	3.44E-30	1.95	1.06E-02	-1.18
A_52_P170685	NAP102507-1	Unknown	5.06E-01	1.10	6.67E-11	-24.21	4.48E-02	4.19
A_51_P235945	Hp,HP-1	haptoglobin	4.21E-01	1.12	0.00E+00	-5.17	6.46E-01	1.28
A_51_P165087	Snai1,Sna,AI194338	snail homolog 1 (Drosophila)	2.91E-09	-1.51	9.25E-01	1.01	2.60E-01	1.07
A_52_P679152	Clk1,STY	CDC-like kinase	3.25E-24	-1.57	4.30E-01	-1.10	4.75E-01	-1.04
A_51_P225781	3830422K02Rik,MGC10	RIKEN cDNA 3830422K02 gene	4.15E-01	-1.05	0.00E+00	-3.14	2.74E-03	-1.19
A_52_P779005	Gbas,AV006093,Nipsna	Glioblastoma amplified sequence (Gbas), mR	2.29E-12	1.62	1.90E-04	2.64	2.04E-01	1.15
A_52_P1188576	Gria2,Glur2,GluR-B,Glu	Glutamate receptor, ionotropic, AMPA2 (alpl	3.10E-04	1.46	4.40E-04	1.66	4.10E-02	1.21
A_51_P328054	Nell2,mel91,R75516,A3	Nel-like 2 homolog (chicken) (Nell2), mRNA	2.27E-09	1.69	9.34E-01	-1.03	7.76E-02	1.55
A_51_P180314	Mup1,Up-1,Ltn-1,Mup-	Major urinary protein 1, mRNA (cDNA clone	1.12E-01	1.83	0.00E+00	-163.81	1.10E-04	35.30
A_51_P507429	Olfr1156,MOR174-3	Mus musculus olfactory receptor MOR174-3	9.08E-01	-1.02	4.00E-05	-1.95	5.02E-01	-1.23
A_51_P240801	2610307O08Rik	RIKEN cDNA 2610307O08 gene, mRNA (cDN/	1.12E-02	-1.12	3.90E-04	-1.58	6.17E-01	1.03
[illegible]	[illegible]	[illegible]	[illegible]	[illegible]	[illegible]	[illegible]	[illegible]	[illegible]

A_51_P435858	TC1070739	MUP3_MOUSE Major urinary protein 3 precu	4.01E-01	-1.22	1.28E-06	-4.54	6.55E-03	6.79
A_52_P378975	Slc1a3,Gmt1,Eaat1,GLA	Solute carrier family 1 (glial high affinity gluta	3.14E-19	1.51	2.45E-01	1.11	6.19E-02	1.10
A_52_P263620	2900027M19Rik	Transmembrane protein 28 (Tmem28), mRN.	3.58E-10	1.51	1.87E-01	-1.08	3.46E-01	1.15
A_52_P478072	Ppp2r2b,SCA12,MGC11	Protein phosphatase 2 (formerly 2A), regulat	6.00E-05	1.90	6.36E-03	1.65	6.99E-01	1.12
A_52_P690983	Camk4,CaMKIV,AI6667	Calcium/calmodulin-dependent protein kinas	8.69E-07	1.54	8.87E-01	-1.06	2.88E-01	1.25
A_52_P337948	Tnik,AI451411,1500031	MKIAA0551 protein	4.00E-05	1.41	1.20E-04	1.57	1.46E-01	1.05
A_51_P304314	AK087320	Mus musculus 0 day neonate lung cDNA, RIK	1.54E-11	1.73	9.61E-01	-1.02	2.76E-02	1.16
A_52_P357445	Fgd4,Frabp,ZFYVE6,933	RIKEN cDNA 9330209B17 gene	1.90E-04	-1.20	2.26E-01	1.59	4.52E-01	-1.07
A_51_P324992	Tnc,TN,Hxb,Ten,TN-C,A	Tenascin C (Tnc), mRNA	2.51E-01	1.14	2.00E-05	-2.98	6.56E-01	-1.09
A_52_P340187	Pon2,AI481612,MGC68	Paraoxonase 2, mRNA (cDNA clone MGC:469	4.56E-03	1.25	3.00E-05	1.83	5.33E-01	1.04
A_51_P254354	V1rc5	Mus musculus vomeronasal 1 receptor, C5 (\	7.00E-05	-2.78	1.50E-04	-3.05	9.83E-01	-1.01
A_52_P8743	AI854408,6330418D12	Expressed sequence AI854408, mRNA (cDNA	7.40E-04	1.34	2.90E-04	1.93	5.23E-01	1.12
A_51_P510466	Pldn,pa,pallid,Stx13bp1	pallidin	3.53E-01	1.05	1.80E-04	1.60	3.60E-01	-1.06
A_51_P306839	1810029B16Rik	RIKEN cDNA 1810029B16 gene	9.37E-02	1.14	4.22E-11	1.99	6.57E-01	-1.04
A_52_P58283	Kcna5,Kv1.5,MGC2524	potassium voltage-gated channel, shaker-rel	8.88E-02	1.10	4.70E-04	1.54	4.24E-01	1.09
A_52_P376119	AK050562	Mus musculus adult pancreas islet cells cDN	1.00E-01	1.15	3.60E-04	2.50	2.31E-01	1.10
A_51_P182106	Unc5cl,BB155270,2510	Unc-5 homolog C (C. elegans)-like (Unc5cl), n	6.82E-01	-1.09	6.24E-10	-1.63	3.95E-01	-1.27
A_51_P114462	Ccl17,TARC,ABCD-2,Scy	chemokine (C-C motif) ligand 17	8.50E-01	1.03	1.70E-35	2.22	9.24E-02	-1.18
A_52_P286360	Otc,Sf,spf,AI265390	ornithine transcarbamylase	4.43E-01	1.52	1.11E-10	-6.92	1.01E-07	9.10
A_51_P323987	B530002L08	hypothetical protein B530002L08	6.13E-01	-1.11	6.92E-10	2.43	2.83E-01	-1.13
A_51_P482908	Arrdc4,2410003C09Rik	RIKEN cDNA 2410003C09 gene	3.36E-01	1.14	8.63E-18	1.72	1.00E-05	-1.23
A_51_P257718	AK052949	Mus musculus 15 days embryo head cDNA, F	7.50E-06	1.60	1.00E-04	2.10	7.37E-02	1.17
A_52_P252872	Rasa1,Gap,Rasa,RasGAI	RAS p21 protein activator 1 (Rasa1), mRNA	3.82E-06	-2.52	8.42E-01	-1.08	6.26E-01	-1.15
A_52_P387292	Pde7b	CAMP specific phosphodiesterase 7B (PDE7B	2.50E-04	1.42	6.00E-04	-1.55	4.17E-01	1.16
A_51_P512820	Dera,DEOC,2010002D2	RIKEN cDNA 2500002K03 gene	4.77E-01	-1.07	1.93E-09	1.55	9.21E-01	-1.01
A_52_P224801	Fbn1,Tsk,Fib-1,AI53646	fibrillin 1	2.32E-01	1.11	1.64E-13	1.61	5.11E-01	-1.03
A_51_P504624	Slc24a6,NCKX6,AF2612	cDNA sequence AF261233	1.83E-02	-1.09	2.00E-05	-1.67	9.66E-03	1.16
A_51_P505653	Mcp,CD46	Membrane cofactor protein (Mcp), mRNA	3.00E-05	1.32	6.90E-02	2.14	3.30E-01	1.12
A_52_P12855	Ankrd43,RP23-188H3.6	hypothetical protein A830006N08	3.50E-04	1.26	2.10E-15	1.57	5.96E-02	1.16
A_51_P263496	Utrn,DRP,Dmdl,AA5895	Utrophin	1.32E-09	1.43	1.05E-03	-1.56	3.79E-01	1.17
A_51_P494812	AK038312	16 days neonate thymus cDNA, RIKEN full-le	1.73E-11	-1.60	1.44E-01	-1.38	4.06E-01	1.07
A_52_P1155674	Spred2,C79158	Spred-2	3.21E-06	-1.54	7.50E-04	-1.49	6.44E-01	-1.08
A_51_P260683	Rgs1,BL34	regulator of G-protein signaling 1	1.35E-26	2.64	3.10E-04	3.63	3.06E-01	1.16
A_52_P483974	Dleu2,Alt1,Leu2,AI1972	Dleu2 mRNA, partial sequence, alternatively	5.05E-01	-1.11	1.16E-35	-1.56	7.37E-01	1.02
A_52_P923591	Grm7,Gpr1g,Gprc1g,m	PREDICTED: Mus musculus glutamate recept	1.75E-38	1.72	1.71E-02	1.40	4.49E-02	1.34
A_52_P80770	1700023B02Rik,Cir,Cicr	RIKEN cDNA 1700023B02 gene	9.57E-01	-1.00	5.10E-09	1.53	5.81E-01	1.03
A_52_P169412	Camk2d,KIAA4163,MG	Calcium/calmodulin-dependent protein kinas	2.15E-01	1.14	9.97E-09	3.17	2.01E-01	1.08
A_51_P413088	Apob,apob-48,AI31505	Mus musculus apolipoprotein B (Apob), mRN	1.98E-02	-1.24	3.00E-05	-2.20	6.40E-04	1.19
A_51_P416910	Slc36a4,PAT4,6330573	solute carrier family 36 (proton/amino acid s	5.92E-01	1.05	9.50E-13	1.74	2.59E-02	-1.19
A_51_P258721	Tpsg1,TMT,Prss31	tryptase gamma 1	3.40E-04	-2.96	1.00E+00	1.00	1.00E+00	1.00
A_52_P298237	Brwd1,Wdr9,5330419I	Bromodomain and WD repeat domain contai	2.85E-08	1.55	1.00E+00	1.00	3.80E-01	1.21
A_52_P963461	Ipo11,Ranbp11,AI3146	Importin 11, mRNA (cDNA clone MGC:39010	1.87E-02	1.30	8.21E-11	2.13	1.00E+00	1.00
A_52_P156796	LOC240038	similar to hypothetical MGC29357 protein	4.52E-01	1.06	2.00E-05	1.53	4.85E-01	-1.11
A_52_P453276	Mid1,Fxy,61B3-R,Trim1	Midline 1 (Mid1), mRNA	7.44E-01	1.05	2.32E-08	-1.57	3.97E-06	1.35
A_52_P492411	TC1024936	AJ278769 gephyrin {Mus musculus}, partial (	1.80E-01	-1.08	3.32E-07	-1.54	3.65E-01	-1.08
A_51_P109508	Havcr2,Tim3,Timd3,MG	hepatitis A virus cellular receptor 2	9.04E-01	-1.01	5.01E-36	2.06	3.89E-01	-1.03
A_51_P115229	V1rg9	Mus musculus vomeronasal 1 receptor, G9 (\	3.67E-01	-1.20	8.00E-05	-2.09	5.73E-01	1.12
A_52_P771857	AK090164	Mus musculus 1 month neonate cerebellum	2.17E-01	1.09	4.43E-14	1.56	8.86E-03	-1.15
A_51_P140641	BC014699,MGC25911,I	cDNA sequence BC014699	4.36E-01	1.03	7.14E-08	1.55	3.90E-01	-1.07
A_51_P331841	4933415L06Rik	RIKEN cDNA 4933415L06 gene	5.58E-01	-1.04	5.21E-25	1.56	2.99E-02	-1.16
A_52_P571832	Hist2h2aa2,H2a-614	Histone 2, H2aa1 (Hist2h2aa1), mRNA	1.23E-07	1.52	5.59E-12	1.84	3.76E-02	-1.17
A_52_P54169	Zbtb20,HOF,DPZF,Oda8	Zinc finger and BTB domain containing 20, m	9.77E-30	1.74	1.07E-01	1.27	4.27E-01	1.10
A_52_P304031	Ppp2r2b,SCA12,MGC11	Protein phosphatase 2 (formerly 2A), regulat	3.00E-05	2.38	1.00E+00	1.00	1.00E+00	1.00
A_51_P472932	TC986321	Unknown	4.06E-01	1.04	4.13E-07	-2.96	7.52E-01	1.02
A_51_P510156	Lcn2,24p3,NGAL,AW21	lipocalin 2	8.00E-05	2.01	2.44E-03	1.27	6.33E-01	-1.05
A_52_P73028	Map4k5,KHS,GCKR,MA	Mitogen-activated protein kinase kinase kina	5.27E-02	1.12	3.28E-06	1.63	3.74E-02	1.14
A_51_P515612	Dgkb,DGK,90kda,DAGK	diacylglycerol kinase, beta	1.90E-04	1.55	1.96E-01	1.33	1.13E-01	-1.30
A_52_P39314	AK087670	Mus musculus 2 days pregnant adult female	3.27E-07	1.74	6.53E-01	-1.24	4.03E-01	1.39
A_52_P645862	Agtr1,AT1,AG2S,AT1a,A	angiotensin receptor 1	9.84E-01	-1.00	6.19E-10	1.54	1.56E-01	1.16
A_51_P496905	Cfi	complement component factor i	3.28E-01	1.20	6.71E-40	-10.01	5.70E-04	10.33
A_52_P30273	Ahd1,Ahd-1,Ssdh1,Aldh	Mus musculus 12 days embryo spinal ganglic	6.00E-05	1.87	1.00E+00	1.00	1.00E+00	1.00
A_52_P488409	Exosc3,Rrp40,AI593501	Mus musculus adult male testis cDNA, RIKEN	4.14E-01	1.13	5.92E-09	1.57	1.67E-01	1.14
A_52_P296026	AK080429	Mus musculus 7 days neonate cerebellum cD	2.19E-15	7.27	1.00E+00	1.00	1.00E+00	1.00
A_51_P326555	1700093K21Rik	RIKEN cDNA 1700093K21 gene (1700093K21	3.75E-01	1.41	1.40E-04	2.26	1.62E-01	-1.21
A_51_P407849	4930502C15Rik	Homeodomain interacting protein kinase 2, r	6.81E-02	1.21	2.34E-06	1.90	5.75E-01	1.11
A_52_P566129	Slc16a14,1110004H10R	Mus musculus solute carrier family 16 (monc	3.99E-12	1.61	2.11E-01	1.28	7.54E-02	2.16
A_51_P180747	Ctla2a,Ctla-2a	cytotoxic T lymphocyte-associated protein 2	1.39E-06	1.56	1.65E-12	1.82	6.54E-03	-1.16
A_52_P207436	Zmym6,Zfp258,AI5932	Zinc finger protein 258, mRNA (cDNA clone I	1.10E-04	1.41	8.00E-05	1.69	2.11E-02	1.31
A_51_P267622	Gjb2,Cx26,Cnx26,Gjb-2	gap junction membrane channel protein beta	2.98E-01	-1.07	6.43E-09	-1.86	5.66E-01	-1.05
A_52_P65351	Sema3c,SemE,Semae,1	Sema domain, immunoglobulin domain (Ig), s	1.70E-04	-1.69	5.32E-01	-1.08	2.85E-01	-1.12
A_51_P517608	2010106E10Rik	RIKEN cDNA 2010106E10 gene	2.00E-04	-1.80	1.00E+00	1.00	6.67E-01	1.22
A_52_P384822	Taok2,PSK,PSK1,TAO1,T	PREDICTED: TAO kinase 2 [Mus musculus], m	5.10E-02	1.17	3.22E-06	1.51	8.13E-01	-1.01
A_51_P417747	Kap,AW146435	kidney androgen regulated protein	5.91E-01	1.06	3.23E-24	-3.48	1.30E-02	1.31
A_52_P139182	TC979638	AF302077 neprilysin-like peptidase gamma {I	4.60E-06	1.79	5.09E-02	2.13	5.42E-01	1.09
A_52_P955515	AK039229	Mus musculus adult male spinal cord cDNA, I	1.15E-21	2.88	9.17E-01	-1.04	3.71E-01	1.53
A_52_P1148772	Igk-V8,8-24,B55L,B128L	Immunoglobulin light chain variable region (I	2.47E-02	-1.16	4.00E-05	-1.86	3.09E-01	1.14
A_52_P127024	D130072O21Rik,C8652	RIKEN cDNA D130072O21 gene, mRNA (cDN.	1.10E-04	1.16	8.79E-08	1.79	3.32E-01	1.07
[illegible]	[illegible]	[illegible]	[illegible]	[illegible]	[illegible]	[illegible]	[illegible]	[illegible]

A_52_P931190	1810013L24Rik,R74903	PREDICTED: Mus musculus RIKEN cDNA 181(	1.33E-01	-1.09	1.00E-05	1.53	5.17E-01	-1.05
A_52_P1116138	AK051319	12 days embryo spinal ganglion cDNA, RIKEN	2.55E-10	-1.55	1.25E-03	1.60	2.18E-01	1.08
A_52_P655371	Klhl20,Kleip,AI504637,k	Kelch-like 20 (Drosophila) (Klhl20), mRNA	3.03E-32	1.78	9.80E-01	-1.01	9.45E-01	1.03
A_51_P483829	C030040K24Rik	Polyadenylate-binding protein-interacting pr	2.34E-23	2.05	5.70E-04	2.30	2.10E-01	1.22
A_52_P322119	9330169L03Rik	Mus musculus RIKEN cDNA 9330169L03 gene	1.69E-01	1.15	4.04E-06	2.31	6.93E-01	-1.05
A_52_P164524	Pcsk1,PC1,PC3,Nec1,SP	proprotein convertase subtilisin/kexin type 1	9.96E-01	1.00	7.13E-10	1.70	7.03E-03	-1.16
A_51_P262757	2310076L09Rik,PAT-1,A	RIKEN cDNA 2310076L09 gene	1.72E-01	1.14	6.20E-17	1.87	5.43E-01	1.07
A_51_P299339	Klf15,CKLF,KKLF,AV048	Kruppel-like factor 15	4.32E-07	1.20	6.58E-15	1.69	9.58E-01	-1.00
A_52_P195706	1700024J04Rik	Adult male testis cDNA, RIKEN full-length enr	9.80E-01	1.00	8.52E-06	1.84	3.81E-01	1.11
A_51_P396978	Kcnip4,Calp,KChIP4,Cal	calsenilin-like protein	7.96E-01	-1.02	1.80E-04	1.53	1.13E-02	-1.08
A_52_P70064	Ng23,G7d,2810436B06	Ng23 protein	4.57E-01	1.04	1.53E-12	1.77	8.88E-01	-1.01
A_52_P7554	Lama4	Mus musculus laminin, alpha 4 (Lama4), mRN	4.68E-01	1.06	4.65E-12	-1.53	6.16E-01	1.04
A_52_P68942	Crk7,Pksc,CrkRS,AI6465	RIKEN cDNA 1810022J16 gene	3.19E-07	1.56	5.56E-03	2.55	4.16E-01	1.13
A_52_P622877	Zic3,Bn	Zinc finger protein of the cerebellum 3 (Zic3)	4.83E-01	-1.05	9.71E-08	1.53	4.77E-01	1.04
A_52_P656254	5430401F13Rik	PREDICTED: hypothetical protein LOC71392	6.12E-01	1.18	6.19E-14	-2.26	3.42E-01	1.77
A_51_P407417	Adamts9,AW743315,18	PREDICTED: Mus musculus a disintegrin-like	2.00E-05	1.27	1.12E-01	1.52	8.03E-01	1.03
A_52_P821	Fli1,Sic1,EWSR2,Fli-1,SI	Friend leukemia integration 1 (Fli1), mRNA	7.02E-01	1.02	6.00E-05	-1.59	3.19E-01	-1.06
A_52_P38499	A830025P08Rik	PREDICTED: similar to Contactin 5 precursor	3.21E-07	1.76	3.19E-12	2.16	8.13E-01	1.06
A_51_P178772	AU018778,MGC18894	hypothetical protein MGC18894	1.00E+00	1.00	3.30E-01	-1.74	5.00E-05	8.50
A_51_P307872	Cyp2j5	cytochrome P450, family 2, subfamily j, polyp	8.94E-02	-1.26	9.89E-07	-3.09	1.95E-01	1.95
A_51_P296456	Ankrd11,Gm176,24101	Ankyrin repeat domain 11, mRNA (cDNA clon	1.47E-07	1.60	5.61E-02	-2.11	4.74E-01	1.15
A_52_P1082755	2900018K06Rik	P21 (CDKN1A)-activated kinase 7, mRNA (cD	1.77E-12	1.53	1.23E-08	-1.37	3.92E-01	1.24
A_52_P531325	Rps6,MGC102571,MGC	Mus musculus adult male hippocampus cDNA	9.31E-01	-1.02	3.89E-27	2.60	2.94E-02	-1.15
A_52_P435009	Gm1533	PREDICTED: hypothetical protein XP_358204	3.41E-01	1.30	8.51E-31	3.10	9.00E-05	-1.65
A_52_P601446	Trim27,Rfp,AW538890	Mus musculus ES cells cDNA, RIKEN full-lengt	3.25E-28	2.60	1.99E-08	3.30	5.51E-01	-1.05
A_52_P494289	Pam	Peptidylglycine alpha-amidating monooxyger	2.05E-03	1.34	8.97E-12	1.71	1.15E-01	-1.13
A_51_P393193	LOC13909,Es31	esterase 31-like	7.22E-01	-1.08	3.34E-22	-29.92	7.23E-07	13.27
A_51_P486188	Pabpc2,PABP,PABP+,Pa	poly A binding protein, cytoplasmic 2	3.98E-02	-1.20	1.72E-08	-2.53	2.20E-04	1.26
A_52_P875947	Kcnh7,erg3,Kv11.3,933	Potassium channel protein (erg3 gene)	1.60E-04	1.87	3.07E-03	1.61	9.28E-01	-1.03
A_51_P261713	Slco1c1,Oatp2,OATP-F,	solute carrier organic anion transporter fami	7.81E-01	-1.01	1.79E-11	-1.52	4.15E-02	-1.07
A_52_P1027847	Xrcc5,Ku80,Ku86,AI314	X-ray repair complementing defective repair	3.19E-07	1.54	4.78E-01	1.41	2.30E-01	1.22
A_52_P240078	2810403A07Rik,AI2563	RIKEN cDNA 2810403A07 gene (2810403A07	1.74E-22	1.58	1.99E-02	1.73	4.78E-03	1.12
A_52_P739568	AK082480	PREDICTED: Mus musculus similar to NPAS3	3.20E-04	1.28	1.82E-06	-1.57	4.54E-01	1.14
A_52_P499902	Sgk3,Cisk,2510015P22F	Serum/glucocorticoid regulated kinase 3 (Sgk	3.60E-02	1.17	2.98E-36	2.06	3.67E-01	1.08
A_52_P1108033	Pbx1,Pbx-1,D230003C0	Pre B-cell leukemia transcription factor 1, mF	1.76E-08	1.57	7.84E-01	-1.03	4.37E-02	1.30
A_52_P62617	LOC433237	hypothetical gene supported by AK028012	2.34E-01	1.08	4.67E-11	1.52	6.56E-01	-1.05
A_52_P596236	D19Ertd737e,2310065I	DNA segment, Chr 19, ERATO Doi 737, expre	4.00E-05	-2.32	1.00E+00	1.00	8.72E-01	1.06
A_51_P461015	Cntnap2,Caspr2,mKIAA	Contactin associated protein-like 2, transcrip	1.50E-04	1.70	1.00E+00	1.00	1.00E+00	1.00
A_52_P597277	NAP102624-1	Unknown	6.48E-02	1.11	5.34E-07	1.55	4.25E-01	-1.05
A_51_P176365	Gimap5,D630024P16,E	RIKEN cDNA E230026N22 gene	5.39E-10	1.32	1.87E-13	1.65	8.03E-02	-1.16
A_51_P285229	FHOS2,KIAA1695,mKIA	formin-family protein FHOS2	6.47E-43	-1.71	6.27E-01	-1.07	2.03E-02	-1.13
A_51_P305008	9530028L01Rik	Pleckstrin homology domain containing, fam	2.00E-04	1.65	8.66E-01	-1.06	7.82E-01	1.10
A_51_P333518	Sh3rf2,RNF158,AI4520	RIKEN cDNA 9130023G24 gene	1.09E-02	1.19	2.26E-06	-1.59	5.06E-01	1.07
A_52_P398730	AA792892	expressed sequence AA792892	3.80E-01	-1.13	8.35E-09	-1.57	1.38E-01	1.17
A_52_P947394	Odz2,Odz3,Ten-m2,mK	ODZ2 (Odz2)	2.40E-09	1.93	9.58E-01	1.02	1.04E-01	1.54
A_51_P289640	1110033M05Rik,53-E6,	RIKEN cDNA 1110033M05 gene, mRNA (cDN	3.33E-06	1.79	1.45E-01	-2.11	7.07E-01	1.10
A_52_P91329	5730410I19Rik,AA5894	RIKEN cDNA 5730410I19 gene	9.79E-01	-1.00	4.80E-04	1.54	2.35E-02	-1.13
A_51_P181891	2600011E07Rik	RIKEN cDNA 2600011E07 gene, mRNA (cDNA	8.10E-04	-1.51	1.00E-05	-1.74	9.07E-03	-1.24
A_51_P123744	Lrfn5,AI427653,AI6048	MKIAA4208 protein	1.88E-02	1.19	7.32E-10	1.88	1.22E-01	-1.12
A_51_P183701	A_51_P183701	Unknown	8.55E-01	-1.02	7.00E-05	-1.52	7.21E-01	1.04
A_52_P526513	E230016M11Rik	Growth arrest and DNA-damage-inducible 45	2.88E-06	1.24	1.18E-16	1.57	2.19E-01	-1.12
A_51_P498882	Cyp2c37	cytochrome P450, family 2. subfamily c, poly	5.22E-02	-1.18	2.31E-15	-10.61	2.88E-10	4.59
A_51_P173114	Pcdh21,Prcad,mKIAA17	photoreceptor cadherin	9.75E-01	1.00	7.00E-05	1.75	3.80E-03	-1.23
A_51_P455338	Ear11	eosinophil-associated ribonuclease 11	3.11E-01	1.28	2.70E-04	-1.69	1.07E-02	-1.55
A_52_P142981	Trappc6b,C79212,5830	Mus musculus 16 days neonate cerebellum c	9.33E-01	1.01	2.22E-06	1.82	2.57E-01	1.11
A_52_P253769	Zcwpw2,4930430K04Ri	PREDICTED: zinc finger, CW type with PWWF	3.26E-01	1.25	2.10E-04	2.80	3.71E-01	-1.63
A_52_P867730	Tubd1,Tubd,4930550G	Tubulin, delta 1, mRNA (cDNA clone MGC:31	9.02E-02	-1.17	4.43E-09	-1.64	2.21E-01	1.13
A_51_P138429	Btc	betacellulin, epidermal growth factor family	4.65E-02	-1.29	7.83E-08	-1.95	2.52E-01	1.13
A_52_P947906	2900024P20Rik,KIAA09	RIKEN cDNA 2900024P20 gene, mRNA (cDNA	1.19E-09	3.98	1.00E+00	1.00	1.90E-01	1.48
A_51_P367866	Egr1,egr,TIS8,Zenk,Egr-	early growth response 1	2.78E-02	-1.16	1.10E-26	-2.40	3.20E-01	-1.22
A_52_P327923	Meox1,Mox1,Mox-1,AI	mesenchyme homeobox 1	6.51E-01	1.03	1.40E-04	-1.70	6.71E-01	1.11
A_52_P550769	Pftk1,mKIAA0834	PFTAIRE protein kinase 1 (Pftk1), mRNA	7.33E-01	1.02	3.00E-05	1.58	1.37E-01	1.29
A_52_P426634	1700018M17Rik	ROD1 regulator of differentiation 1 (S. pomb	1.60E-03	1.33	2.60E-07	1.69	4.10E-01	1.16
A_52_P1116291	Sh3yl1,Ray,YSC84,AI31	Mus musculus adult retina cDNA, RIKEN full-l	1.90E-04	-1.73	1.00E+00	1.00	7.27E-01	1.08
A_52_P507745	Cadps2,Cpd2,A230044	Ca2+-dependent activator protein for secreti	1.07E-06	1.88	8.36E-01	1.08	8.02E-01	1.06
A_52_P1084368	Ttc19,AI505442,20102	Tetratricopeptide repeat domain 19, mRNA (	1.75E-01	-1.11	4.72E-08	-1.77	3.14E-01	1.20
A_52_P137778	A530053G22Rik	RIKEN cDNA A530053G22 gene	1.30E-01	1.18	2.10E-18	2.30	5.23E-01	1.13
A_52_P827357	Phr1,Pam,R75243,AU0	Pam, highwire, rpm 1 (Phr1), mRNA	7.00E-05	1.22	8.04E-03	1.55	6.26E-01	1.05
A_52_P416752	AK085146	Mus musculus 13 days embryo lung cDNA, R	6.85E-30	1.63	1.89E-01	1.16	3.40E-04	1.31
A_52_P427405	A130099P19Rik	LIM and senescent cell antigen-like domains	2.10E-04	-1.20	3.91E-01	-1.69	3.60E-01	1.08
A_52_P229439	NAP046044-1	Unknown	4.00E-05	1.46	2.15E-01	1.57	1.83E-01	1.11
A_52_P291428	1700001E04Rik	RIKEN cDNA 1700001E04 gene	1.23E-01	1.08	6.00E-05	1.57	5.52E-01	-1.03
A_52_P280360	Rbm3,2600016C11Rik	RNA binding motif protein 3	1.93E-10	-2.92	1.62E-02	-2.33	5.87E-01	-1.08
A_52_P231410	Polr3e,Sin,RPC5	Polymerase (RNA) III (DNA directed) polypep	2.61E-15	1.94	6.55E-11	1.35	8.10E-07	-1.40
A_51_P488243	Erbb3,Her3,C76256,Erb	V-erb-b2 erythroblastic leukemia viral oncog	2.16E-01	-1.27	2.01E-07	-5.61	2.11E-01	-1.44
A_51_P495780	S3-12,mKIAA1881	plasma membrane associated protein, S3-12	2.09E-02	-1.17	1.40E-45	4.67	4.00E-04	1.30
A_52_P203560	Fzd10,Fz-10	frizzled homolog 10 (Drosophila)	3.67E-02	-1.16	3.40E-10	-1.80	9.43E-01	1.00
[illegible]	[illegible]	[illegible]	[illegible]	[illegible]	[illegible]	[illegible]	[illegible]	[illegible]

A_52_P987766	AK037330	Mus musculus 16 days neonate thymus cDN	7.00E-05	1.62	1.16E-01	1.95	6.86E-01	1.10
A_52_P328492	TC994428	X83413 U88 {Human herpesvirus 6}, partial (	4.79E-01	1.06	2.60E-04	1.53	3.25E-02	-1.12
A_52_P11581	1110019B24Rik	PREDICTED: Mus musculus zinc finger protei	2.57E-01	-1.07	1.64E-09	1.56	4.17E-01	1.12
A_51_P403636	Smad7,Madh7	MAD homolog 7 (Drosophila)	6.97E-01	1.02	2.03E-06	1.57	5.64E-01	-1.05
A_51_P181354	5330430B06Rik	Mus musculus adult male pituitary gland cDN	3.42E-24	2.05	3.04E-01	-1.66	1.75E-01	-1.43
A_52_P129183	9430064G09Rik	MKIAA0458 protein	2.85E-23	1.66	6.83E-01	1.26	7.49E-01	1.04
A_51_P140803	Slco1b2,OATP2,Oatp4,l	solute carrier organic anion transporter fami	1.65E-01	1.36	2.00E-05	-3.11	7.79E-02	3.24
A_51_P394172	Es1,Ee-1,Es-1,Es-4,Es-N	esterase 1	7.48E-01	-1.14	2.83E-29	-11.83	1.25E-08	8.52
A_52_P155302	Ankib1,C80642,AW494	PREDICTED: Mus musculus ankyrin repeat ar	3.85E-01	-1.12	2.90E-04	1.74	1.61E-01	-1.13
A_51_P127858	Dnase1l2,MGC118464,	deoxyribonuclease 1-like 2	8.07E-06	-1.43	2.40E-04	-1.68	2.35E-01	-1.09
A_52_P699109	1700065D16Rik	Protein kinase ATR (Atr)	5.94E-06	2.46	1.00E+00	1.00	2.34E-01	1.71
A_52_P400163	Ndufs4,C1-18k	NADH dehydrogenase (ubiquinone) Fe-S prot	4.00E-05	1.55	1.00E+00	1.00	5.69E-01	1.04
A_51_P142196	H19	H19 fetal liver mRNA, mRNA (cDNA clone MC	3.24E-01	-1.11	1.70E-04	-1.96	4.83E-01	1.20
A_52_P578790	Dok3,Dokl,AI450713	expressed sequence AI450713	5.57E-01	-1.08	1.61E-11	-1.58	1.89E-01	-1.07
A_51_P267754	Icam2,CD102,Ly-60,Icar	intercellular adhesion molecule 2	7.00E-01	1.02	3.50E-12	-1.84	6.85E-01	-1.03
A_52_P432949	Erbb3,Her3,C76256,Erb	V-erb-b2 erythroblastic leukemia viral oncog	5.03E-01	-1.05	4.04E-22	-2.30	8.51E-01	-1.03
A_52_P517011	9530091C08Rik	RIKEN cDNA 9530091C08 gene	3.00E-05	2.06	8.13E-01	-1.09	8.20E-01	-1.05
A_52_P501447	LOC278757	similar to hypothetical protein 6720451E15	7.76E-01	1.01	4.00E-05	1.54	7.64E-01	-1.03
A_52_P243152	Disp1,DispA,1190008H	RIKEN cDNA 1190008H24 gene	5.26E-03	1.21	1.41E-21	1.51	1.45E-01	-1.09
A_51_P463562	Mpa2,Gbp4,Mag-2,Mpa	macrophage activation 2	7.25E-03	-1.37	4.71E-08	-3.21	3.56E-03	-1.25
A_52_P149200	1300003B13Rik	RIKEN cDNA 1300003B13 gene	6.29E-01	1.02	7.00E-05	1.54	3.12E-01	-1.12
A_52_P602098	Za20d1,CEZANNE,Trabi	Zinc finger, A20 domain containing 1 (Za20d	5.53E-17	1.71	7.04E-01	1.11	8.75E-01	1.04
A_51_P298023	Ugt2a3,2010321J07Rik	RIKEN cDNA 2010321J07 gene	3.10E-01	-2.07	5.00E-05	-4.05	4.00E-05	17.86
A_52_P1067916	Gli3,Xt,Bph,Pdn,add,AI	GLI-Kruppel family member GLI3 (Gli3), mRN	6.47E-10	1.68	5.38E-02	-2.34	5.11E-02	1.55
A_52_P406449	TC988784	Unknown	3.34E-01	1.12	4.36E-13	1.59	1.35E-03	-1.41
A_51_P233707	AI851076,AL022898,M	Expressed sequence AI851076 (AI851076), m	2.32E-01	1.19	5.00E-05	1.52	6.88E-01	1.03
A_52_P365770	BC026657,Skt,6620402	CDNA sequence BC026657, mRNA (cDNA clo	2.00E-05	2.14	1.00E+00	1.00	5.33E-01	1.19
A_51_P245796	Ddit4,dig2,REDD1,Rtp8	RIKEN cDNA 5830413E08 gene	7.60E-26	1.57	7.26E-03	1.48	5.54E-01	-1.04
A_51_P408082	Apoa1,Sep2,Alp-1,Ltw-	apolipoprotein A-I	4.49E-01	1.07	2.69E-12	-4.82	1.04E-01	2.30
A_52_P441070	Apob,apob-48,AI31505	Mus musculus apolipoprotein B (Apob), mRN	6.54E-01	-1.25	0.00E+00	-28.61	8.77E-08	13.66
A_51_P397783	Anubl1,2810002D23Rik	Mus musculus RIKEN cDNA 2810002D23 gen	4.04E-01	1.07	1.44E-24	2.15	9.39E-01	1.01
A_52_P35861	Rxrip110,RIP110,94300	Retinoid X receptor interacting protein 110,	2.30E-04	1.71	3.15E-01	1.95	9.33E-02	-1.53
A_51_P349961	Gc,DBP	Group specific component, mRNA (cDNA clo	4.90E-01	-1.10	6.38E-14	-9.09	6.78E-03	5.54
A_52_P222775	Map3k6,MAPKKK6	mitogen-activated protein kinase kinase kina	1.07E-03	1.25	0.00E+00	2.80	6.29E-01	-1.04
A_52_P574306	Apoc3,Apoc	apolipoprotein C-III	4.12E-01	1.05	6.00E-05	-1.64	1.26E-02	1.53
A_52_P141112	Csnk1g3,3300002K07Ri	RIKEN cDNA 3300002K07 gene	6.61E-01	1.02	1.04E-06	1.62	6.34E-01	-1.03
A_51_P378336	Coq3,C77934,4732433	coenzyme Q3 homolog, methyltransferase (y	7.73E-01	-1.02	1.57E-10	1.61	5.85E-01	-1.03
A_52_P662562	NAP122883-1	Unknown	2.54E-06	-1.32	4.46E-02	1.61	1.85E-01	1.26
A_51_P213910	AK078689	Adult male eyeball cDNA, RIKEN full-length e	5.87E-13	2.09	6.47E-01	1.24	7.81E-01	-1.11
A_52_P858929	9330112M16	hypothetical protein 9330112M16	4.67E-12	2.50	5.61E-01	1.34	1.00E+00	1.00
A_51_P241368	Ptbp2,Ptb2,nPTB,brPTB	Polypyrimidine tract binding protein 2, mRN	9.34E-13	1.65	7.45E-01	1.15	3.65E-01	1.08
A_51_P314547	AK019935	Mus musculus adult male pituitary gland cDN	3.88E-06	1.75	1.00E+00	1.00	6.69E-02	1.59
A_52_P981004	Vps13d,BC037490	PREDICTED: Mus musculus vacuolar protein	1.03E-09	-2.22	4.00E-04	-3.13	1.44E-01	2.26
A_52_P68440	TC1030512	CT2B_MOUSE CTLA-2-beta protein precursor	5.93E-30	1.89	1.68E-06	1.93	5.09E-01	-1.07
A_51_P335888	9430087N24Rik	Mus musculus 12 days embryo embryonic bo	8.65E-01	1.02	8.81E-07	-1.98	5.38E-01	1.18
A_52_P347270	NAP028505-1	Unknown	4.38E-02	-1.30	1.00E-04	-1.63	1.77E-01	1.15
A_52_P435426	Dusp11,AI481497,2010	dual specificity phosphatase 11 (RNA/RNP co	3.95E-02	-1.19	1.40E-09	-1.60	5.32E-01	1.03
A_52_P477489	Eef1d,AL023999,17000	Eukaryotic translation elongation factor 1 de	9.24E-01	-1.02	9.00E-05	-1.57	1.74E-01	1.06
A_52_P598495	Serpina1e,PI5,Dom5,Sp	serine (or cysteine) proteinase inhibitor, clad	7.09E-01	1.04	1.40E-04	-4.10	6.32E-03	3.60
A_52_P101252	D630045D17Rik	RIKEN cDNA D630045D17 gene	7.91E-28	1.79	4.20E-04	1.47	5.68E-02	1.21
A_51_P262340	Rbm3,2600016C11Rik	RNA binding motif protein 3, mRNA (cDNA cl	7.29E-01	-1.08	2.97E-37	1.96	5.00E-05	-1.86
A_52_P400106	Car5b,CAVB,CarVb,733	Carbonic anhydrase 5b, mitochondrial, mRN	2.00E-05	3.23	7.20E-01	1.17	4.07E-01	-1.36
A_52_P372843	Fusip1,Nssr,TASR,NSSR	FUS interacting protein (serine-arginine rich)	0.00E+00	3.01	6.16E-03	1.53	1.21E-03	1.25
A_52_P686785	Xlkd1,Lyve-1,1200012G	extra cellular link domain-containing 1	2.99E-01	1.13	8.65E-06	1.53	3.73E-02	1.22
A_52_P185643	AK045887	Adult male corpora quadrigemina cDNA, RIK	1.50E-04	-1.94	1.42E-01	-1.33	8.71E-01	1.01
A_52_P500337	Nap1l3,MB20	nucleosome assembly protein 1-like 3	3.22E-01	-1.05	1.30E-04	1.52	3.78E-01	-1.08
A_52_P1077539	St3gal5,3S-T,[a]2,Siat9	ST3 beta-galactoside alpha-2,3-sialyltransfer	2.50E-04	1.23	9.46E-03	2.09	2.43E-02	1.25
A_52_P493882	2810403D21Rik,AU017	15 days embryo head cDNA, RIKEN full-lengt	3.65E-01	-1.09	1.56E-07	1.51	2.86E-09	-1.22
A_52_P493322	D330038O06Rik	hypothetical protein D330038O06	2.04E-02	-1.15	1.20E-04	1.73	4.02E-02	-1.13
A_52_P771575	5430420P03Rik,Plmp	PREDICTED: Mus musculus RIKEN cDNA 543	1.89E-15	1.79	5.56E-01	1.35	3.26E-01	1.28
A_52_P947485	AK046241	Mus musculus adult male corpora quadrigem	4.00E-05	1.77	4.03E-01	-1.38	5.35E-01	1.38
A_52_P1123509	AK033876	Mus musculus adult male diencephalon cDN	2.28E-11	1.53	1.00E+00	1.00	8.67E-01	-1.07
A_52_P105206	Cdadc1,AI449174,AI46	Cytidine and dCMP deaminase domain conta	7.88E-01	-1.03	4.00E-05	1.96	7.10E-01	-1.04
A_51_P349652	Lao1,AW990848	L-amino acid oxidase 1	7.81E-02	1.30	1.27E-22	8.32	1.37E-03	1.75
A_52_P365732	Cntn2,Tax,TAG-1,D130	Contactin 2 (Cntn2), transcript variant 1, mR	1.13E-01	-1.10	1.26E-11	-1.80	1.05E-03	-1.16
A_52_P610113	Cdh19	Mus musculus similar to cadherin 19, type 2	2.98E-03	-1.19	1.50E-04	1.68	1.61E-01	-1.11
A_51_P361650	Saa1,Saa2,Saa-1	serum amyloid A 1	3.24E-01	-1.10	4.21E-24	-4.17	1.67E-02	2.63
A_52_P413515	A730095J18Rik	Zinc finger protein, subfamily 1A, 2 (Helios) (	1.52E-02	-1.16	4.16E-10	1.51	4.28E-01	-1.05
A_51_P240036	Serpina3k,RP54,Spi2,M	serine (or cysteine) proteinase inhibitor, clad	8.74E-01	1.04	0.00E+00	-10.60	6.96E-07	7.18
A_51_P247114	H2-Bl,H2-B1	histocompatibility 2, blastocyst	2.40E-03	1.33	8.20E-19	1.80	3.10E-01	-1.08
A_52_P528600	Hhex,Hex,Prh,Prhx	hematopoietically expressed homeobox	3.00E-05	-2.14	1.16E-01	-1.45	2.85E-01	-1.13
A_52_P223646	Cabyr,CBP86,FSP-2,MG	calcium-binding tyrosine-(Y)-phosphorylatio	3.30E-02	1.12	4.39E-14	1.77	7.69E-01	1.02
A_51_P242006	6430590I03Rik	PREDICTED: similar to melanoma antigen fan	7.16E-01	1.02	6.11E-07	1.52	1.40E-04	1.18
A_52_P67643	Frap1,FRAP,flat,mTOR,	FK506 binding protein 12-rapamycin associa	8.17E-01	-1.03	5.97E-06	2.34	4.01E-02	-1.18
A_51_P212427	Lama4	Mus musculus laminin, alpha 4 (Lama4), mR	5.19E-01	1.05	3.00E-05	-1.52	3.50E-01	-1.04
A_52_P315766	Cyp2j5	cytochrome P450, family 2, subfamily j, poly	4.30E-02	-2.00	2.16E-08	-3.87	3.00E-05	23.88
A_52_P556908	Dlx6	Dlx6 mRNA, 3' end	9.82E-01	-1.00	2.94E-06	1.57	2.44E-01	-1.05
[illegible]	[illegible]	[illegible]	[illegible]	[illegible]	[illegible]	[illegible]	[illegible]	[illegible]

A_51_P266274	AK077975	Mus musculus 13 days embryo male testis cD	3.65E-10	1.51	5.63E-01	1.22	3.88E-01	1.21
A_52_P192071	Cflar,Cash,Flip,MRIT,CL	CASP8 and FADD-like apoptosis regulator	5.89E-02	1.10	2.60E-04	1.54	2.43E-01	-1.07
A_52_P924138	ORF34,D930001I21Rik	Hypothetical protein C130073P14, mRNA (cD	1.50E-07	1.51	3.24E-01	1.38	1.46E-02	1.22
A_52_P188656	Lonrf3,Rnf127,4932412	Mus musculus 8 days embryo whole body cD	1.92E-14	1.71	2.89E-01	1.72	3.49E-01	1.16
A_51_P499776	Denr,1500003K04Rik	density-regulated protein	8.53E-01	-1.02	2.39E-22	1.59	4.20E-02	-1.16
A_52_P787779	AK080443	Mus musculus 7 days neonate cerebellum cD	1.63E-01	-1.39	1.50E-04	-1.59	3.17E-01	1.16
A_52_P336259	Mll3,KIAA1506,mKIAA1	Mus musculus similar to myeloid [XM_35557	2.10E-04	1.19	1.39E-06	1.66	6.03E-01	1.06
A_51_P444290	Slamf8,Blame,SBBI42,5	SLAM family member 8, mRNA (cDNA clone I	8.00E-05	-1.42	2.16E-01	-1.81	3.99E-01	-1.11
A_52_P31632	B930018B01	hypothetical protein B930018B01	9.42E-01	-1.01	8.14E-20	1.74	9.09E-01	1.01
A_52_P1044323	2810022L02Rik,AA9871	RIKEN cDNA 2810022L02 gene (2810022L02I	7.00E-05	1.43	3.28E-02	-1.71	1.41E-01	1.25
A_52_P467528	E130008O17Rik	R3H domain (binds single-stranded nucleic a	3.39E-29	-1.57	2.70E-04	-1.27	9.21E-01	1.01
A_51_P460786	Zfp532,mKIAA1629,C53	Zinc finger protein 532, mRNA (cDNA clone N	1.48E-28	1.82	1.00E+00	1.00	4.77E-01	1.45
A_52_P21951	A930002H24Rik	PREDICTED: RIKEN cDNA A930002H24 gene		2.68E-12	1.66	3.19E-02	1.49	2.78E-01	1.12
A_52_P430577	NAP042151-1	Unknown	3.70E-04	-1.53	9.50E-01	-1.01	3.43E-01	1.11
A_52_P678281	6030404K05Rik	RIKEN cDNA 6030404K05 gene	1.06E-02	1.19	2.00E-04	2.96	3.02E-01	-1.18
A_52_P134195	Ceacam10,Bgp3,Cea10	CEA-related cell adhesion molecule 10	4.15E-01	-1.06	4.00E-05	1.51	7.82E-02	-1.32
A_51_P477121	Pmaip1,Noxa	phorbol-12-myristate-13-acetate-induced pr	3.10E-04	1.40	1.44E-15	2.22	4.75E-01	1.04
A_52_P201154	Trmu,Trmt1,AI314320,	TRNA 5-methylaminomethyl-2-thiouridylate	6.00E-05	1.88	3.00E-05	1.77	7.44E-01	-1.04
A_52_P79441	BC033596	Mus musculus cDNA sequence BC033596 (B	1.11E-01	1.22	4.00E-05	3.17	4.09E-01	1.30
A_51_P436813	5430405H02Rik,26103	TGFB-induced factor 2 (Tgif2), mRNA	5.18E-08	1.58	8.71E-02	1.37	9.85E-01	-1.00
A_52_P260366	Gpm6b,M6B,Gpm6,AI5	Glycoprotein m6b, mRNA (cDNA clone MGC:	3.30E-04	1.50	1.83E-02	2.31	2.90E-04	1.24
A_52_P413851	Xrn1,exo,Dhm2,mXrn1	5'-3' exoribonuclease 1 (Xrn1), mRNA	1.28E-02	1.14	6.64E-14	1.64	8.78E-02	1.13
A_51_P331570	Trib3,Nipk,SINK,Trb3,Ifl	induced in fatty liver dystrophy 2	2.20E-04	1.52	8.02E-01	1.02	2.80E-03	1.22
A_52_P1179800	AK032313	Mus musculus adult male olfactory brain cD	1.08E-01	1.18	4.77E-06	2.13	5.56E-02	1.41
A_52_P330524	B930011P16Rik,Gm137	Mus musculus RIKEN cDNA B930011P16 gen	2.32E-10	1.56	9.85E-01	-1.00	8.99E-01	1.01
A_51_P419801	Bach2,MGC117590	BTB and CNC homology 2 (Bach2), mRNA	2.84E-01	-1.07	6.10E-08	-1.79	4.90E-01	1.07
A_52_P97670	D530031C13Rik	RIKEN cDNA D530031C13 gene	1.30E-04	1.24	1.99E-11	1.54	5.99E-01	1.09
A_51_P110301	C3,ASP,Plp,AI255234	complement component 3	2.69E-01	-1.08	0.00E+00	-3.53	4.90E-01	1.39
A_52_P439874	Arfgap3,Arfgap1,06100	Mus musculus adult male cecum cDNA, RIKE	8.38E-01	1.03	1.00E-05	-1.81	8.85E-01	1.03
A_52_P89884	Gm784,MGC100196,M	Gene model 784, (NCBI) (Gm784), mRNA	3.64E-03	-1.20	5.00E-05	-1.74	4.71E-01	1.12
A_52_P135198	4833444G19Rik	RIKEN cDNA 4833444G19 gene	2.30E-04	1.85	1.00E+00	1.00	2.51E-01	1.77
A_52_P282000	Enah,Mena,WBP8,Ndp	Mus musculus adult male colon cDNA, RIKEN	6.00E-05	1.30	9.31E-06	1.86	3.22E-01	1.12
A_51_P186421	5730409E15Rik,A6300	RIKEN cDNA 5730409E15 gene, mRNA (cDNA	3.11E-01	1.06	3.90E-04	1.58	1.69E-01	-1.13
A_52_P550124	1700112J16Rik	Adult male testis cDNA, RIKEN full-length en	8.68E-01	1.02	2.80E-04	1.53	6.35E-02	-1.19
A_52_P698754	Zfp146,OZF	Zinc finger protein 146 (Zfp146), mRNA	4.26E-11	1.66	6.41E-01	1.24	6.00E-01	1.09
A_51_P262699	Cpb2,CPR,Cpu,TAFI,AI2	Carboxypeptidase B2 (plasma) (Cpb2), mRNA	6.77E-01	-1.11	3.25E-23	-7.21	9.00E-05	35.66
A_51_P489522	Ctla2b,Ctla-2b,AW5455	Cytotoxic T lymphocyte-associated protein 2	7.29E-06	1.64	7.20E-14	1.94	6.90E-04	-1.17
A_52_P236072	Mtf1,MTF-1	metal response element binding transcriptio	3.25E-01	1.10	3.69E-10	1.51	2.77E-01	-1.07
A_51_P478783	Dennd2d,MGC6915,AI4	DENN/MADD domain containing 2D, mRNA (	2.30E-04	-3.02	8.46E-01	1.10	9.38E-01	1.02
A_52_P404329	Saa4,Saa-4,Saa-5	serum amyloid A 4	8.00E-01	-1.08	5.29E-03	-2.98	4.19E-06	5.56
A_52_P416367	Asah2,AI585898	Mus musculus 3 days neonate thymus cDNA,	1.40E-04	1.78	1.00E+00	1.00	3.46E-02	1.61
A_51_P506532	Olfr1022,MOR196-1	Mus musculus olfactory receptor MOR196-1	3.69E-01	-1.40	2.02E-13	-2.35	9.46E-01	1.05
A_52_P139439	A_52_P139439	Unknown	7.45E-01	-1.04	8.78E-21	-1.92	6.77E-01	-1.02
A_52_P82023	NAP054202-1	Unknown	1.24E-01	1.12	1.14E-11	-1.55	2.29E-01	1.10
A_51_P157362	Uhmk1,KIS,Kist,AA6735	kinase interacting with leukemia-associated	3.27E-16	-1.77	8.53E-01	-1.08	9.38E-01	1.02
A_52_P264924	Ctsw,lymphopain	cathepsin W	2.82E-01	1.06	2.19E-07	-1.61	3.52E-01	-1.11
A_52_P257919	9830001H06Rik	MKIAA0889 protein	1.47E-06	1.30	2.93E-11	1.63	4.65E-01	1.06
A_51_P458067	P4ha1,P4ha,AL022634	procollagen-proline, 2-oxoglutarate 4-dioxyg	3.98E-01	1.08	3.36E-09	-1.53	8.02E-01	-1.01
A_52_P389274	Sfi1,mKIAA0542,23100	Sfi1 homolog, spindle assembly associated (y	2.00E-05	-1.51	9.40E-02	-1.33	4.10E-01	-1.06
A_52_P678056	2610027C15Rik,MGC3	RIKEN cDNA 2610027C15 gene	4.16E-01	-1.07	3.00E-05	-1.88	7.50E-04	1.65
A_52_P412585	Ear1	eosinophil-associated ribonuclease 1	2.10E-01	1.54	1.30E-04	-1.88	6.30E-03	-1.81
A_52_P684037	Ncam1,CD56,Ncam,E-N	neural cell adhesion molecule 1	4.14E-01	-1.05	2.78E-09	1.52	2.61E-03	-1.16
A_52_P779997	AA674995	Transcribed locus	1.60E-03	1.28	4.00E-05	2.32	3.94E-01	1.09
A_52_P967353	Als2cr13	Mus musculus RIKEN cDNA 2810425F24 gen	1.15E-01	1.12	7.78E-12	1.51	4.24E-01	-1.04
A_51_P274465	Sycp3,Cor1,Scp3	synaptonemal complex protein 3	2.21E-02	1.21	3.30E-06	1.64	5.30E-01	1.20
A_51_P202541	Polr3e,Sin,RPC5	sex-lethal interactor homolog (Drosophila)	2.72E-29	1.64	2.66E-24	2.66	3.41E-01	-1.10
A_52_P1091857	Tex12,5730518K06Rik	Testis protein TEX12 (Tex12)	3.80E-08	1.41	2.70E-04	2.63	8.91E-01	1.02
A_51_P375453	Wnt9a,Wnt14	Wingless-type MMTV integration site 9A (Wr	5.95E-02	-1.12	1.16E-10	-1.51	3.58E-02	1.16
A_51_P304002	BC026657,Skt,6620402	CDNA sequence BC026657, mRNA (cDNA clo	4.12E-06	1.65	7.20E-01	1.22	9.57E-02	1.21
A_52_P1195769	L1Md-Tf30	Mus musculus 12 days embryo embryonic bo	4.23E-02	-1.13	4.76E-20	-1.99	1.88E-01	1.13
A_51_P153132	Kcne2,MiRP1,AW04827	potassium voltage-gated channel, Isk-related	1.19E-02	-1.21	4.86E-28	-1.56	1.61E-03	-1.29
A_52_P1100772	Plec1,PCN,EBS1,PLTN,A	B6-derived CD11 +ve dendritic cells cDNA, RI	5.23E-13	1.47	1.32E-03	1.59	6.30E-01	1.03
A_52_P443676	9330119M13Rik	RIKEN cDNA 9330119M13 gene	4.81E-18	1.88	8.33E-02	1.36	2.79E-01	1.15
A_52_P460584	Tnfrsf25,DR3,TR3,Wsl,D	tumor necrosis factor receptor superfamily, r	1.00E-05	-2.02	1.67E-03	-1.83	7.07E-01	-1.05
A_52_P44914	MGC25972	similar to cytochrome P450, 4a10	7.72E-01	-1.03	1.18E-08	-1.99	3.87E-02	1.74
A_52_P637812	Prrg4,TMG4,MGC1175	RIKEN cDNA 9930111I18 gene	1.40E-01	-1.10	1.30E-04	-1.78	1.32E-01	1.54
A_51_P259930	Apoa5,RAP3,Apoav,130	apolipoprotein A-V	6.88E-01	-1.07	8.83E-03	-3.16	2.25E-06	3.98
A_52_P153479	Nsun6,NOPD1,4933403	NOL1/NOP2/Sun domain family 6 (Nsun6), m	2.00E-05	1.92	2.31E-02	2.11	3.73E-01	1.17
A_52_P188309	Gpatc3,MGC106307,D	G patch domain containing 3, mRNA (cDNA c	7.92E-03	1.14	1.40E-04	1.58	1.89E-01	-1.08
A_52_P599478	ENSMUST0000005033	Unknown	9.38E-02	-1.11	6.01E-07	-1.60	9.22E-01	-1.01
A_51_P154840	1700080G18Rik	Nuclear respiratory factor 1, mRNA (cDNA cl	5.47E-06	1.25	2.16E-15	1.54	8.88E-01	1.01
A_52_P40257	Cd59a,Cd59,AA987121,	CD59a antigen	6.52E-01	-1.02	6.53E-06	1.68	6.82E-02	-1.17
A_51_P355753	Hic1,HIC-1,AA408311	hypermethylated in cancer 1	5.01E-03	-1.28	5.94E-09	-1.92	9.81E-01	1.00
A_52_P860077	Rai16,G430067P06Rik	Retinoic acid induced 16, mRNA (cDNA clone	2.00E-07	1.43	2.69E-03	1.86	5.49E-01	-1.05
A_51_P321886	Cklfsf3,BNAS2,AI41389	chemokine-like factor super family 3	3.48E-01	1.04	3.78E-29	2.20	2.43E-01	1.06
A_52_P354682	Elovl7,AI840082,91300	RIKEN cDNA 9130013K24 gene	6.11E-01	1.04	5.34E-16	-1.51	1.01E-03	-1.15
A_51_P432288	Rnf183,5830442J12Rik	RIKEN cDNA 5830442J12 gene	1.60E-01	-1.58	9.00E-05	-1.71	6.49E-01	-1.17
[illegible]	[illegible]	[illegible]	[illegible]	[illegible]	[illegible]	[illegible]	[illegible]	[illegible]

A_51_P507669	Rn18s	M.musculus mRNA for external transcribed s	3.30E-08	-2.16	1.96E-03	-1.22	2.16E-09	2.75
A_52_P164570	Hsd17b12,Kik1,KIK-I,AI1	Mus musculus 10, 11 days embryo whole bo	8.93E-19	-1.58	4.80E-04	-1.49	6.82E-03	-1.37
A_51_P394474	Pde6b,r,rd,rd1,Pdeb,rd	phosphodiesterase 6B, cGMP, rod receptor,	4.40E-01	1.11	2.60E-04	-2.21	6.30E-01	1.26
A_52_P250433	B230303O12Rik	Mus musculus adult male corpora quadrigem	8.51E-08	1.71	4.00E-05	1.66	6.77E-01	1.04
A_52_P279184	Srp54,54kDa	signal recognition particle 54	9.71E-01	1.00	1.28E-07	1.91	3.42E-02	-1.13
A_52_P647488	Zfml,Np220,mNP220,AI	Zinc finger, matrin-like, mRNA (cDNA clone II	4.12E-13	1.69	2.90E-04	1.57	2.28E-01	-1.08
A_51_P460739	Amph,MGC49429	Amphiphysin, mRNA (cDNA clone IMAGE:535	1.60E-06	1.65	6.27E-01	-1.25	1.06E-01	-1.78
A_52_P571006	Cyp3a44	cytochrome P450, family 3, subfamily a, poly	1.04E-01	-1.17	0.00E+00	-25.41	1.58E-08	7.83
A_52_P371541	BY667523	Transcribed locus	3.64E-01	1.04	0.00E+00	3.80	2.51E-01	1.24
A_52_P964088	Odz3,Odz1,Ten-m3,mK	ODZ3 (Odz3)	7.28E-21	2.10	8.81E-01	-1.03	1.53E-02	1.27
A_52_P313713	9230101K24Rik	RIKEN cDNA 9230101K24 gene	4.22E-22	-1.77	1.31E-01	-1.37	3.00E-05	-1.15
A_52_P31625	A430079E08	hypothetical protein A430079E08	3.11E-01	-1.09	8.63E-10	-1.89	9.63E-02	1.10
A_51_P166648	4931408A02Rik,MGC11	C21orf63 protein (C21orf63)	9.92E-01	-1.00	1.46E-26	1.57	6.87E-01	-1.03
A_52_P94721	Cacna2d1,Cacna2,Cchl2	Calcium channel, voltage-dependent, alpha2	2.02E-10	2.42	1.00E+00	1.00	1.00E+00	1.00
A_51_P113825	9430091E24Rik	PREDICTED: hypothetical protein XP_489247	7.95E-01	-1.06	2.15E-06	-2.75	8.61E-01	1.10
A_52_P590756	Slc25a35,1810012H11R	Solute carrier family 25, member 35 (Slc25a3	1.81E-03	-1.25	4.00E-05	-1.70	1.44E-01	1.08
A_51_P217273	4933429H19Rik	Translocation protein 1, mRNA (cDNA clone I	5.25E-03	1.24	8.18E-06	2.11	3.42E-01	-1.14
A_52_P159490	Ppp1r9a,BB181831,mK	protein phosphatase 1, regulatory (inhibitor)	8.40E-04	1.34	2.00E-04	1.66	5.16E-01	1.06
A_51_P341852	Usp53,Sp6,Phxr3,AA93	Ubiquitin specific peptidase 53, mRNA (cDNA	5.20E-04	1.43	1.06E-18	2.29	1.88E-02	1.13
A_51_P498093	Hes1,Hry	hairy and enhancer of split 1 (Drosophila)	9.54E-01	1.00	1.80E-14	1.52	2.26E-02	-1.10
A_52_P67463	2810013C04Rik,281004	PREDICTED: hypothetical protein LOC69170	3.30E-06	-1.52	1.36E-02	-1.27	1.53E-01	-1.08
A_52_P644490	Spata6,Hash,Mash,AI79	Kinesin-related protein HASH (Hash)	2.00E-05	1.44	3.15E-02	1.52	2.79E-01	1.15
A_51_P458262	Ccl19,ELC,CKb11,MIP3E	chemokine (C-C motif) ligand 19	3.89E-02	1.15	1.23E-19	1.52	1.26E-01	-1.09
A_51_P138238	Olfr1348,MOR103-9	olfactory receptor MOR103-9	6.47E-01	-1.06	5.42E-08	-1.83	5.85E-01	1.14
A_52_P723501	AK080195	PREDICTED: Mus musculus similar to triosepl	2.68E-02	-1.19	8.38E-07	-2.30	1.30E-04	1.23
A_51_P120295	Cyp2d26,1300006E06R	cytochrome P450, family 2, subfamily d, poly	3.17E-01	1.09	3.06E-17	-2.32	6.14E-02	2.34
A_52_P628127	BU936742	Adult male testis cDNA, RIKEN full-length enr	4.26E-01	-1.04	2.91E-07	2.57	7.82E-01	1.02
A_52_P858226	2810452K05Rik	RIKEN cDNA 2510006D16 gene (2510006D16	1.02E-19	1.80	2.75E-14	2.04	3.57E-01	1.07
A_52_P1164289	Nck2,Grb4,NCKbeta	Non-catalytic region of tyrosine kinase adapt	2.76E-01	1.08	2.09E-09	1.63	8.51E-01	-1.04
A_52_P522048	4930555G01Rik	RIKEN cDNA 4930555G01 gene	1.69E-01	1.11	2.00E-05	1.58	2.74E-01	-1.07
A_51_P471900	Stk3,MST,Mst3,mess1,	Serine/threonine kinase 3 (Ste20, yeast hom	1.61E-14	1.44	5.00E-04	1.50	8.72E-02	1.14
A_51_P222264	BC026657,Skt,6620402	CDNA sequence BC026657, mRNA (cDNA clo	4.25E-14	1.76	1.00E+00	1.00	7.24E-01	1.07
A_51_P117711	Bat2d,Bat2d1,mKIAA10	BAT2 domain containing 1, mRNA (cDNA clor	5.34E-01	1.08	3.60E-04	-5.37	3.00E-01	1.24
A_52_P97229	Trpm3,MLSN2,LTRPC3,	Transient receptor potential cation channel,	5.90E-24	1.61	5.00E-02	1.26	9.15E-01	1.02
A_51_P162649	Upf3b,UPF3X,RENT3B,	UPF3 regulator of nonsense transcripts hom	4.34E-01	1.05	3.70E-04	1.53	3.94E-01	-1.05
A_51_P178806	AK045389	Adult male corpora quadrigemina cDNA, RIKI	1.33E-07	1.66	3.00E-05	1.49	4.20E-01	1.13
A_51_P482868	V1rc25	vomeronasal 1 receptor, C25	1.03E-01	-1.24	1.72E-03	-1.78	4.00E-05	1.31
A_51_P239984	Exo1,Msa	exonuclease 1	1.16E-07	-1.82	8.50E-01	-1.05	5.12E-01	-1.11
A_51_P180629	Cdc42ep1,CEP1,Borg5,I	CDC42 effector protein (Rho GTPase binding	7.11E-01	-1.01	1.98E-11	-1.68	2.63E-01	1.10
A_51_P387868	1700010L19Rik	PREDICTED: hypothetical protein LOC71840	2.79E-01	1.06	4.93E-36	1.89	5.74E-01	1.05
A_52_P124352	Ifrd1,PC4,Ifnl,Tis7	Interferon-related developmental regulator 1	7.93E-09	1.76	5.97E-19	2.20	7.39E-01	-1.05
A_52_P995993	Papd4,AI649009,80304	PAP associated domain containing 4 (Papd4)	6.81E-03	1.20	1.70E-04	1.63	2.79E-01	-1.17
A_51_P301603	AI854517	PREDICTED: hypothetical protein LOC101694	2.15E-01	1.18	2.80E-17	1.92	2.67E-01	-1.06
A_51_P480796	4930547N16Rik,AI5036	Pro-melanin-concentrating hormone (Pmch),	9.17E-01	-1.02	2.00E-05	3.21	7.98E-01	-1.10
A_52_P234544	NAP027406-1	Unknown	9.48E-01	1.00	1.20E-04	1.52	6.95E-01	-1.03
A_51_P451377	Kng1,Kng,MGC123437	kininogen	1.00E+00	1.00	1.50E-34	-12.64	2.91E-08	10.46
A_52_P476904	Chd6,5430439G14Rik,6	CDNA clone IMAGE:6819153	1.03E-07	-1.37	1.57E-02	-1.98	5.86E-01	-1.06
A_52_P338651	Pitpnm2,NIR3,Rdgb2,RI	retinal degeneration B2 homolog (Drosophila	4.68E-01	1.06	1.20E-11	1.74	1.40E-01	-1.09
A_52_P554720	Slc17a3,Npt4,AW26172	expressed sequence AW261723	8.23E-01	1.10	7.14E-06	-4.41	1.00E+00	1.00
A_51_P125009	Pvrl3,AA407785,AU016	poliovirus receptor-related 3	5.01E-01	1.04	5.10E-07	-1.60	8.66E-01	1.02
A_52_P30006	Adamts19,AU015154,4	a disintegrin-like and metalloprotease (repro	3.35E-01	-1.06	3.17E-12	2.00	2.21E-10	-1.35
A_52_P747012	C230040D10Rik,Gm110	Premature mRNA for mKIAA0865 protein	4.72E-07	2.76	1.05E-02	2.66	4.90E-01	1.71
A_51_P105755	2610041B18Rik	RIKEN cDNA 2610041B18 gene	2.00E-05	-1.36	1.27E-02	-1.54	9.85E-02	-1.23
A_52_P114282	MGC38735	hypothetical LOC494468	6.38E-01	1.04	4.88E-11	1.51	4.93E-01	-1.11
A_52_P1162808	2310026I22Rik	Mus musculus adult male tongue cDNA, RIKE	3.38E-02	-1.22	2.41E-06	-2.37	9.00E-05	1.27
A_52_P593110	Ptbp2,Ptb2,nPTB,brPTB	Polypyrimidine tract binding protein 2, mRN	4.42E-03	1.25	1.07E-07	2.30	1.19E-01	-1.14
A_51_P342387	Apoh,B2GPI,beta2-GPI,	apolipoprotein H	1.04E-01	-1.12	0.00E+00	-2.79	5.57E-02	1.14
A_52_P900367	AK080565	7 days neonate cerebellum cDNA, RIKEN full-	7.69E-01	-1.03	2.00E-04	1.52	8.42E-01	1.02
A_51_P111796	AW146020,GCF2,A130	Expressed sequence AW146020 (AW146020	3.58E-02	1.18	6.85E-11	1.50	5.99E-01	1.04
A_52_P589735	Acp1,Acp-1,LMW-PTP,	Acid phosphatase 1, soluble, mRNA (cDNA cl	2.62E-02	1.16	6.20E-11	1.52	3.75E-02	-1.08
A_52_P1059487	6330510M09Rik	Potassium voltage-gated channel, Shal-relate	1.72E-01	1.09	7.00E-05	1.94	1.12E-01	-1.09
A_52_P683525	9330169L03Rik	Mus musculus RIKEN cDNA 9330169L03 gen	6.31E-07	1.90	6.73E-01	-1.18	8.86E-01	1.04
A_52_P632412	Spfh2,C87251,BC03633	cDNA sequence BC036333	3.90E-01	1.06	3.30E-09	1.51	9.56E-01	-1.00
A_52_P1116115	Grm3,Gprc1c,mGluR3,C	Glutamate receptor, metabotropic 3, mRNA	7.89E-03	-1.48	4.16E-07	-1.66	3.47E-01	1.16
A_51_P432011	AK015109	Mus musculus adult male testis cDNA, RIKEN	1.46E-08	1.76	5.20E-03	1.70	5.05E-01	1.14
A_52_P1036640	Dgki,C130010K08Rik	PREDICTED: RIKEN cDNA C130010K08 gene [	4.42E-06	1.56	5.66E-10	1.32	1.87E-01	1.18
A_51_P102257	E030037J05Rik	PREDICTED: Mus musculus RIKEN cDNA E030	4.44E-06	1.86	6.19E-02	1.99	5.68E-01	1.15
A_51_P118237	Sgk,Sgk1	serum/glucocorticoid regulated kinase	0.00E+00	1.73	7.85E-12	3.47	6.98E-01	1.04
A_51_P422113	Cdh2,CDHN,Ncad,N-cad	Cadherin 2, mRNA (cDNA clone MGC:36119 I	3.00E-09	1.77	1.00E+00	1.00	8.47E-01	-1.07
A_52_P395381	Zfp282,HUB1,C81429,A	Zinc finger protein 282 (Zfp282), mRNA	2.00E-04	1.58	7.21E-01	1.16	6.88E-01	1.19
A_51_P108812	S100a5,S100D9	S100 calcium binding protein A5	4.49E-01	1.45	1.01E-31	4.71	6.09E-02	-2.25
A_52_P203948	Pla2g4e,C230096D22,2	Pla2g4e mRNA for cytosolic phospholipase A	9.34E-01	-1.01	9.39E-24	2.05	5.16E-02	-1.13
A_52_P443547	D330012F22Rik	RIKEN cDNA D330012F22 gene, mRNA (cDNA	5.70E-08	1.31	2.14E-06	2.22	1.09E-01	-1.12
A_52_P161365	Clock,mKIAA0334,5330	circadian locomoter output cycles kaput	9.59E-40	1.61	1.18E-02	1.94	2.25E-01	1.08
A_51_P123745	BC011467,MGC37865	CDNA sequence BC011467, mRNA (cDNA clo	1.57E-01	1.08	1.71E-17	2.19	4.09E-01	1.05
A_52_P423810	Mt1,MT-I,Mt-1	Metallothionein 1, mRNA (cDNA clone MGC:	3.14E-14	1.54	8.08E-12	1.62	5.29E-01	-1.06
A_52_P318673	Saa2,Saa1,Saa-2,AW11	Serum amyloid A 2, mRNA (cDNA clone MGC	3.38E-01	1.04	1.13E-26	-2.37	8.96E-01	1.01
[illegible]	[illegible]	[illegible]	[illegible]	[illegible]	[illegible]	[illegible]	[illegible]	[illegible]

A_51_P464270	B230206N24Rik,63305(	RIKEN cDNA B230206N24 gene	2.05E-07	-1.56	1.27E-01	-1.27	7.90E-01	-1.02
A_51_P365351	Dhdh,AA591799,13000	RIKEN cDNA 1300018L09 gene	4.04E-02	-1.16	3.17E-06	-2.31	4.26E-01	1.08
A_51_P143495	E130103I17Rik	RIKEN cDNA E130103I17 gene, mRNA (cDNA	4.74E-01	1.12	6.06E-15	2.22	5.32E-01	1.10
A_51_P173043	Egr2,Egr-2,Zfp-6,Krox2(	early growth response 2	1.65E-11	-2.24	8.91E-36	-6.41	5.45E-03	-2.48
A_51_P489406	Jmjd1a,TGSA,Tsga,Jmjd	Jumonji domain containing 1A, mRNA (cDNA	1.12E-22	1.95	8.90E-04	2.27	9.78E-01	1.00
A_52_P110581	A930011G23Rik	PREDICTED: RIKEN cDNA A930011G23 gene		1.56E-11	1.54	7.75E-01	-1.06	1.32E-01	1.26
A_52_P699762	6130401L20Rik	PREDICTED: hypothetical protein LOC75740		3.06E-01	1.07	3.37E-15	1.73	1.95E-02	-1.24
A_51_P279454	AK039163	Mus musculus adult male hypothalamus cDN	7.47E-06	2.72	2.00E-05	3.28	1.00E+00	1.00
A_52_P467563	Atp8a1,APLT,Atp3a2,Cl	ATPase, aminophospholipid transporter (APL	6.20E-07	1.89	4.43E-01	-1.37	4.73E-01	1.47
A_51_P272672	AK041319	Mus musculus adult male aorta and vein cDN	1.35E-18	1.61	3.00E-05	2.00	5.71E-01	-1.08
A_52_P288284	Hif3a,Ipas,MOP7	hypoxia inducible factor 3, alpha subunit	6.02E-19	1.58	9.38E-19	2.70	2.54E-01	1.21
A_52_P220275	Gpr34	G protein-coupled receptor 34	3.08E-01	-1.08	2.25E-06	-1.50	2.99E-01	-1.06
A_52_P624155	TC1038455	ENPL_MOUSE Endoplasmin precursor (Endo		2.00E-05	-1.54	5.00E-05	-1.59	3.35E-01	-1.13
A_51_P295681	AK084888	13 days embryo lung cDNA, RIKEN full-length	4.13E-03	1.35	1.09E-07	1.51	8.35E-02	-1.15
A_52_P564663	TC1013806	Unknown	5.00E-05	1.54	2.90E-04	1.88	1.24E-01	-1.12
A_51_P362176	Fgg,AI256424,3010002I	fibrinogen, gamma polypeptide	5.95E-01	-1.10	4.19E-14	-41.58	1.76E-01	3.09
A_52_P555603	Apbb2,TR2L,Zfra,Rirl1,2	Amyloid beta (A4) precursor protein-binding,	1.43E-08	1.60	7.92E-01	1.10	1.35E-01	1.21
A_52_P677276	4833441J24Rik,AI4500!	RIKEN cDNA 4833441J24 gene (4833441J24F	2.73E-01	-1.15	5.55E-06	-1.57	6.24E-01	1.03
A_51_P309874	BB531021	0 day neonate lung cDNA, RIKEN full-length e	3.39E-25	1.63	2.07E-01	-1.19	6.61E-01	1.11
A_52_P699236	Angpt2,Ang2,Agpt2,An	Angiopoietin 2, mRNA (cDNA clone MGC:252	8.71E-02	-1.34	1.65E-03	-1.76	2.00E-05	1.31
A_51_P212012	8430419K02Rik	16 days embryo lung cDNA, RIKEN full-length	0.00E+00	2.65	2.41E-07	2.90	1.97E-02	1.26
A_52_P1152469	Arid1b,Ardi1b,AI83695!	MKIAA1235 protein	3.88E-26	1.55	2.47E-01	-1.05	2.29E-01	1.15
A_52_P265584	1810011O10Rik,AW121	Mus musculus adult male heart cDNA, RIKEN	2.47E-10	1.69	1.48E-16	2.66	4.23E-01	1.05
A_52_P521042	BC029214,MGC36831,[	cDNA sequence BC029214	1.59E-02	1.19	8.06E-20	1.50	3.55E-01	-1.06
A_51_P352924	Fem1b,mKIAA0396	feminization 1 homolog b (C. elegans)	6.54E-03	1.30	4.44E-08	1.61	3.71E-01	-1.04
A_52_P665404	Htf9c	Mus musculus 8 days embryo whole body cD	1.10E-04	-1.61	8.01E-01	-1.10	9.13E-01	1.02
A_52_P523532	Oxa1l,AI894287,18100	Oxidase assembly 1-like, mRNA (cDNA clone	7.98E-02	1.15	7.00E-05	1.56	9.74E-01	1.00
A_51_P407227	Gimap6,Ian6,FLJ00102,	immune associated nucleotide 6	1.20E-04	1.28	3.80E-13	1.54	2.43E-07	-1.41
A_51_P472659	1200004M23Rik,AU01	RIKEN cDNA 1200004M23 gene	9.42E-02	-1.07	1.55E-09	-1.66	5.99E-02	-1.08
A_51_P364529	Capn7,PalBH,AU02231!	Calpain 7 (Capn7), mRNA	2.00E-05	1.45	8.29E-02	-2.75	7.58E-01	1.09
A_52_P1172633	4930405O22Rik	PREDICTED: Mus musculus similar to actin (L	3.00E-05	1.64	6.04E-01	-1.16	3.27E-01	1.32
A_52_P137710	Zkscan1,KOX18,C87323	Zinc finger with KRAB and SCAN domains 1 (Z	9.31E-01	-1.01	2.70E-04	3.03	5.90E-01	-1.03
A_51_P215896	Tsc22d4,Tilz2,Thg-1pit,	B6-derived CD11 +ve dendritic cells cDNA, RI	5.24E-01	1.08	5.24E-10	-1.72	2.34E-01	1.19
A_52_P17746	NAP102790-1	Unknown	9.45E-01	-1.00	2.00E-05	1.57	4.87E-01	-1.07
A_52_P491718	Mbtps2,AI662535,MGC	Membrane-bound transcription factor peptic	1.18E-12	1.47	1.68E-02	1.73	1.49E-02	1.17
A_51_P489367	Cyp3a25,AI327008	cytochrome P450, family 3, subfamily a, poly	6.31E-01	-1.23	1.42E-01	-1.51	5.32E-07	14.65
A_51_P374900	P2ry13,GPCR1,GPR94,G	G protein-coupled receptor 86	3.70E-02	-1.12	2.51E-07	-2.02	1.19E-01	1.05
A_52_P134874	D130054N24Rik	RIKEN cDNA D130054N24 gene, mRNA (cDN	3.21E-09	1.53	8.37E-01	1.11	3.43E-01	-1.26
A_51_P276993	Gpr84,EX33	G protein-coupled receptor 84	1.70E-01	-1.27	6.85E-06	-2.15	1.09E-02	1.25
A_52_P580984	NAP053619-1	Unknown	6.44E-08	1.71	7.54E-01	-1.05	7.00E-01	-1.04
A_51_P244303	Smc1l2,SMC1beta	SMC (structural maintenace of chromosome:	6.18E-01	-1.07	2.90E-04	2.09	6.58E-01	-1.18
A_51_P275808	Trim59,Mrf1,TSBF1,231	RIKEN cDNA 2310035M22 gene	1.70E-04	-1.40	3.52E-03	-1.74	8.91E-02	-1.23
A_52_P1028096	AK046956	Mus musculus 10 days neonate cerebellum c	2.00E-21	1.57	1.50E-01	-2.15	3.24E-01	1.17
A_51_P212191	Txnl6,RdCVF,MGC2760	RIKEN cDNA A930031O08 gene	7.33E-01	1.17	5.07E-06	-3.77	6.86E-01	-1.17
A_51_P344932	Tnfaip6,Tsg6,TSG-6,Tnf	Tumor necrosis factor alpha induced protein	3.57E-08	-1.63	2.55E-01	1.15	1.03E-02	-1.20
A_52_P513196	1700065A05Rik,AA420	Mus musculus adult male diencephalon cDN	8.84E-03	1.35	4.81E-07	2.47	3.73E-01	1.12
A_51_P481325	Ier2,ch1,pip92,AI31723	immediate early response 2	1.81E-24	-1.55	2.69E-08	-1.92	1.05E-01	-1.15
A_52_P1061446	Baz1a,Acf1,Gtl5,cbp14	Bromodomain adjacent to zinc finger domair	3.25E-01	-1.09	2.12E-06	-1.74	6.83E-01	-1.03
A_51_P155514	Prl,AV290867	prolactin	2.60E-03	-1.91	5.53E-01	-1.75	0.00E+00	-32.91
A_52_P499711	Dusp11,AI481497,2010	dual specificity phosphatase 11 (RNA/RNP cc	1.79E-03	-1.16	7.38E-09	-1.65	9.86E-01	1.00
A_52_P835749	Eif4e,If4e,eIF-4E,Eif4e-		Eukaryotic translation initiation factor 4E, mF	7.03E-11	-1.54	7.12E-01	1.09	1.15E-01	-1.16
A_52_P491684	9630041I06Rik	Calcium/calmodulin-dependent protein kinas	6.04E-02	-1.29	3.00E-05	-1.94	2.90E-01	1.12
A_52_P690909	Gm1752,Gm611,MGC1	Mus musculus adult male diencephalon cDN	3.69E-02	-1.15	5.00E-05	1.63	2.24E-02	-1.23
A_52_P207654	TC992986	AJ539223 erythroid differentiation regulator	5.94E-01	1.07	5.19E-08	-1.64	4.00E-05	1.29
A_52_P177922	Ankrd11,Gm176,24101	Ankyrin repeat domain 11, mRNA (cDNA clor	6.94E-02	1.16	8.15E-07	1.71	9.21E-01	1.01
A_52_P485417	Hmgb2,Hmg2,HMG-2,C	PREDICTED: similar to high mobility group pr	1.57E-01	1.11	1.67E-09	2.00	7.78E-01	1.02
A_52_P304136	Kcns2	K+ voltage-gated channel, subfamily S, 2	8.13E-01	-1.03	4.15E-18	-1.79	7.82E-01	-1.03
A_52_P273169	Sh3d19,Kryn,AW0117!	SH3 domain protein D19	5.43E-01	1.04	2.00E-05	-6.23	9.29E-01	-1.02
A_52_P284535	Ftmt,MtF,Fth3,493044	RIKEN cDNA 4930447C24 gene	9.88E-01	-1.00	1.00E-05	-1.54	1.62E-01	-1.09
A_51_P199778	AK034273	Mus musculus adult male diencephalon cDN	3.31E-02	1.11	0.00E+00	1.73	2.25E-02	-1.12
A_52_P1075797	A330032P22Rik	PREDICTED: hypothetical protein XP_488543	1.31E-03	1.38	6.79E-08	1.78	3.85E-01	1.07
A_51_P205008	Lst1,B144	Mus musculus leukocyte specific transcript 1	1.95E-01	1.05	2.41E-11	-2.14	2.94E-01	-1.07
A_51_P152211	Zfp386,Kzf1,KIAA4205,i	zinc finger protein 386 (Kruppel-like)	1.16E-01	1.10	7.00E-05	1.59	2.01E-01	-1.09
A_52_P348256	Mup1,Up-1,Ltn-1,Mup-	Major urinary protein 1, mRNA (cDNA clone	5.25E-01	1.09	1.40E-14	-10.48	1.73E-01	1.83
A_52_P779691	AK084291	12 days embryo eyeball cDNA, RIKEN full-len	8.43E-10	1.34	5.32E-07	-1.60	6.20E-01	1.10
A_51_P412508	Irx4	Iroquois related homeobox 4 (Drosophila)	5.12E-01	-1.06	1.00E-05	-1.56	4.10E-01	1.12
A_52_P21574	Pdlim7,Enigma,AV0079	PDZ and LIM domain 7 (Pdlim7), mRNA	1.83E-01	-1.06	3.31E-06	-1.53	6.25E-01	-1.03
A_51_P398791	Dmd,mdx,pke,Dp427,D	Dystrophin, muscular dystrophy (Dmd), mRN	2.00E-05	1.47	3.56E-01	-1.75	4.64E-01	-1.07
A_52_P411305	Tsc1,hamartin,mKIAA0	Mus musculus 2 days neonate thymus thymi	5.36E-10	1.55	5.69E-11	1.51	2.21E-01	1.14
A_52_P207303	Bmp3,9130206H07,953	bone morphogenetic protein 3	1.37E-01	-1.13	1.54E-14	-1.97	2.39E-01	1.09
A_52_P135000	AK081948	PREDICTED: Mus musculus similar to ribosor	7.17E-13	-1.93	7.96E-01	-1.06	2.76E-01	-1.07
A_52_P683432	D12Ertd551e,CHR2SYT,	DNA segment, Chr 12, ERATO Doi 551, expre	2.55E-01	-1.10	8.00E-05	1.58	2.87E-01	1.12
A_52_P422088	Ptbp2,Ptb2,nPTB,brPTB	Polypyrimidine tract binding protein 2, mRN	3.29E-11	1.80	6.64E-01	1.08	3.93E-01	1.11
A_52_P134317	Purg,4930486B15Rik	purine-rich element binding protein G	1.41E-02	-1.36	7.61E-19	-1.80	2.26E-01	-1.09
A_52_P651	Mrc2,uPARAP,Endo180	Mannose receptor, C type 2 (Mrc2), mRNA	8.63E-01	1.02	2.80E-04	2.36	7.27E-01	1.07
A_51_P115655	Iqcd,AI662646,493343	RIKEN cDNA 4933433C09 gene	5.03E-01	-1.13	3.50E-07	-1.57	3.90E-01	1.38
A_52_P174456	Rufy2,Denn,LZ-FYVE,ZF	RUN and FYVE domain-containing 2	1.42E-02	1.26	4.00E-05	1.93	3.32E-01	-1.09
[illegible]	[illegible]	[illegible]	[illegible]	[illegible]	[illegible]	[illegible]	[illegible]	[illegible]

A_51_P484976	AK046202	Mus musculus adult male corpora quadrigem	1.02E-02	-1.24	9.26E-10	-1.89	2.14E-01	1.12
A_51_P497741	4930434E21Rik	Mus musculus RIKEN cDNA 4930434E21 gen	3.12E-01	-1.17	7.00E-05	1.68	9.73E-01	1.00
A_52_P891479	6430702L21Rik,AI8503	PREDICTED: RIKEN cDNA 6430702L21 [Mus r	5.30E-11	1.73	8.12E-01	1.15	1.00E+00	1.00
A_52_P467948	2810485I05Rik,C78303	RIKEN cDNA 2810485I05 gene	9.35E-01	-1.01	2.34E-12	1.81	7.89E-01	-1.02
A_52_P430927	Zfyve26,Gm893,KIAA03	Zinc finger, FYVE domain containing 26, mRN	2.32E-01	1.10	3.30E-04	1.50	2.82E-01	-1.11
A_51_P419840	Trhde,MGC40831,9330	TRH-degrading enzyme (Trhde), mRNA	2.66E-14	1.54	9.60E-01	1.02	7.51E-03	1.23
A_51_P230634	Phactr3,H17739,SCAPIN	MKIAA4224 protein	1.19E-11	2.12	4.04E-14	5.08	7.54E-01	1.06
A_52_P1092263	AK048920	PREDICTED: Mus musculus hypothetical prot	6.99E-13	1.52	1.56E-01	-1.07	7.31E-01	1.03
A_52_P448027	1700022C02Rik,492151	RIKEN cDNA 1700022C02 gene	4.47E-01	-1.08	3.84E-06	1.59	8.40E-04	-1.16
A_51_P377557	D11Ertd636e,0610010	DNA segment, Chr 11, ERATO Doi 636, expre	6.15E-06	1.30	5.28E-31	-2.30	9.00E-05	1.31
A_51_P383689	Fkbp5,Dit1,51kDa,FKBP	FK506 binding protein 5	3.82E-01	1.06	0.00E+00	2.90	7.03E-02	1.17
A_52_P26386	LOC433801	similar to RIKEN cDNA 6330416L07 gene	3.68E-02	1.14	6.24E-10	1.62	2.94E-01	-1.15
A_51_P306215	3100002H09Rik	13 days embryo head cDNA, RIKEN full-lengt	8.00E-05	1.63	1.58E-03	-1.21	1.28E-01	-1.16
A_51_P355301	Cyp3a11,Pcn,Cyp3a,IIIA	cytochrome P450, family 3, subfamily a, poly	6.34E-01	1.06	4.21E-15	-3.14	3.88E-02	-1.14
A_52_P167125	Ppp3cb,Calnb,CnAbeta,	Calmodulin dependent phosphatase catalytic	7.92E-08	1.51	7.86E-01	1.19	4.90E-01	1.15
A_51_P338803	Pigt,CGI-06,4930534E1	Mus musculus RIKEN cDNA 4930534E15 gen	6.60E-10	-1.72	1.98E-01	1.11	4.70E-02	1.46
A_52_P508131	Capn11,calpain,proteas	PREDICTED: Mus musculus calpain 11 (Capn1	5.40E-07	-7.28	1.00E+00	1.00	5.61E-01	1.27
A_51_P503138	Rab31,AI415285,17000	RAB31, member RAS oncogene family (Rab31	6.79E-15	1.73	5.26E-01	1.39	1.59E-01	1.11
A_52_P193424	4930562C03Rik,MGC10	PREDICTED: Mus musculus RIKEN cDNA 4930	7.63E-01	1.02	5.19E-07	1.93	1.19E-01	-1.07
A_52_P72831	Tro,Tnn,Trol,Maged3,M	Mus musculus trophinin (Tro), mRNA [NM_2	4.71E-07	-1.63	8.44E-03	1.26	6.72E-10	1.50
A_52_P510038	Zfp81,Zfp78,KRAB13,Hs	Mus musculus adult female vagina cDNA, RIK	2.41E-12	1.34	1.15E-03	1.57	5.31E-01	-1.07
A_51_P279437	Mfsd2,1700018O18Rik	Major facilitator superfamily domain contain	4.16E-02	1.07	5.22E-28	1.83	3.26E-01	1.06
A_51_P515639	Csf3r,Cd114,Csfgr,G-CS	colony stimulating factor 3 receptor (granulo	2.65E-02	1.14	1.19E-08	-1.54	6.72E-01	1.02
A_51_P267063	Ugt3a2,AI313915,MGC	expressed sequence AI313915	1.94E-01	-1.11	7.00E-05	-1.54	1.52E-02	1.50
A_52_P578732	Ccr5,AM4-7,CD195,Cml	chemokine (C-C motif) receptor 5	4.40E-04	-1.17	7.13E-07	-2.13	1.27E-03	-1.14
A_52_P436085	NAP054776-1	Unknown	1.81E-02	1.26	2.66E-06	-2.69	1.55E-01	1.62
A_52_P826666	Rap2ip,Rpip8	Rap2 interacting protein (Rap2ip), mRNA	4.20E-04	-1.44	2.87E-01	-1.52	9.97E-01	-1.00
A_52_P345229	Foxb1,C43,Mf3,Twh,Fkl	forkhead box B1	6.03E-07	-1.31	1.57E-06	-1.64	1.92E-01	-1.12
A_51_P333309	Sh3bgrl,1190008F14Rik	SH3-binding domain glutamic acid-rich prote	1.33E-06	1.59	7.56E-01	1.14	4.04E-01	1.71
A_51_P235816	Tsc22d3,DIP,Gilz,Dsip1,	TSC22 domain family 3, mRNA (cDNA clone M	1.46E-01	1.08	1.06E-25	1.84	2.74E-01	-1.09
A_52_P19894	Fnbp1l,TOCA1,AW5482	RIKEN cDNA 2610318I01 gene	1.85E-01	-1.06	1.55E-07	1.59	6.03E-01	1.06
A_52_P1132648	Nrxn1,mKIAA0578,170	Neurexin I (Nrxn1), mRNA	3.36E-06	1.53	7.10E-02	1.42	7.09E-01	-1.06
A_51_P218196	Msx1,msh,Hox7,Hox-7,	homeo box, msh-like 1	2.76E-11	-1.57	4.25E-03	-1.33	4.39E-01	-1.07
A_52_P492189	TC1084647	Unknown	8.42E-08	1.40	2.99E-01	1.63	3.70E-01	1.19
A_52_P755486	Trim33,Tif1g,AI413936,	Tripartite motif protein TRIM33 (Trim33)	5.58E-14	1.60	1.00E+00	1.00	6.28E-01	1.25
A_51_P284946	Rnd3,Arhe,Rhoe,AI661	ras homolog gene family, member E	5.40E-04	-1.22	3.94E-15	-1.59	3.90E-01	-1.07
A_52_P608322	Maff	v-maf musculoaponeurotic fibrosarcoma onc	1.07E-16	2.14	1.75E-33	2.65	2.10E-01	-1.11
A_52_P1132414	Prkca,Pkca	Protein kinase C, alpha (Prkca), mRNA	1.63E-11	1.53	8.94E-01	1.02	1.54E-01	1.21
A_52_P679536	Cdc42bpa,DMPK-like,A	Mus musculus adult retina cDNA, RIKEN full-	1.55E-09	1.60	1.00E+00	1.00	5.61E-01	1.15
A_52_P341569	B230343H07Rik	Adult male corpora quadrigemina cDNA, RIKI	2.54E-01	-1.06	4.72E-12	1.52	3.62E-03	-1.18
A_52_P1164184	Kcnd2,Kv4.2,R75121,AI	Potassium voltage-gated channel, Shal-relate	1.00E-05	1.66	1.00E+00	1.00	5.52E-01	1.11
A_52_P272348	TC990838	BC031479 Als2 protein {Mus musculus}, part	2.19E-03	-1.36	1.15E-08	-1.58	7.62E-01	1.02
A_51_P389377	Rnf148,Greul3,4933432	Ca2+-dependent activator protein for secreti	1.07E-06	1.61	1.00E+00	1.00	8.98E-01	1.04
A_52_P99810	Cx3cr1	chemokine (C-X3-C) receptor 1	3.56E-01	1.07	1.00E-04	-1.52	3.74E-01	1.07
A_51_P191046	2410007B07Rik	ES cells cDNA, RIKEN full-length enriched libr	2.79E-02	-1.23	8.24E-02	-1.69	7.96E-06	1.28
A_51_P200731	Kcnma1,Slo,Slo1,mSlo,I	Large conductance calcium activated potassi	1.69E-12	1.53	3.68E-02	-1.14	1.51E-01	1.24
A_52_P2015	Gabarapl2,Gef2,GATE-1	GABA(A) receptor-associated protein like 2	5.28E-01	1.12	3.20E-16	1.59	1.60E-01	-1.22
A_51_P482051	Cyp3a16	cytochrome P450, family 3, subfamily a, poly	7.21E-01	-1.14	0.00E+00	-21.18	4.22E-07	9.09
A_52_P307739	Sox2,lcc,ysb,Sox-2	SRY-box containing gene 2	5.00E-05	-1.23	4.66E-21	-1.55	5.93E-01	1.05
A_51_P301007	Spata19,AV047838,170	RIKEN cDNA 1700001L23 gene	1.91E-01	1.14	1.98E-09	1.51	9.85E-01	1.00
A_52_P295955	Exosc9,p5,p6,RRP45,Pn	Mus musculus 12 days embryo spinal ganglic	1.51E-32	1.88	1.09E-07	2.44	6.74E-02	1.17
A_52_P55772	Tbxa2r,TP,MGC107665	thromboxane A2 receptor	7.22E-01	-1.03	4.79E-08	-1.80	7.80E-01	1.02
A_52_P232507	Mup3,MUP15,Mup-3,M	Mus musculus major urinary protein 3 (Mup3	8.55E-01	-1.08	0.00E+00	-16.59	8.66E-08	14.03
A_51_P212308	Cxadr,CAR,MCAR,AU01	coxsackievirus and adenovirus receptor	1.00E-05	1.56	1.47E-01	1.54	9.07E-03	1.16
A_52_P317258	Grip1,eb,KIAA4223,mKI	Glutamate receptor interacting protein 1 (Gr	1.44E-09	1.78	1.52E-01	1.50	6.01E-01	1.16
A_52_P1075671	A730017C20Rik	RIKEN cDNA A730017C20 gene, mRNA (cDN/	2.10E-23	1.70	1.40E-01	-1.65	6.51E-02	1.10
A_52_P365491	0710005M24Rik	RIKEN cDNA 0710005M24 gene, mRNA (cDN	8.17E-01	1.08	8.08E-13	2.88	1.00E+00	1.00
A_51_P115147	Pdzk3,Gm82,KIAA0300,	MKIAA0300 protein	1.98E-03	1.32	4.00E-05	1.98	1.19E-02	1.34
A_52_P548680	TC1050945	U55772 p170 phosphatidylinositol 3-kinase {	8.84E-01	-1.01	2.86E-15	1.66	9.21E-01	1.00
A_52_P47748	D6Ertd160e	DNA segment, Chr 6, ERATO Doi 160, expres	3.02E-12	2.38	1.00E+00	1.00	8.79E-02	1.50
A_52_P390844	Olfr740,MOR106-4	Mus musculus olfactory receptor MOR106-4	3.27E-01	-1.22	5.51E-11	-1.91	8.93E-01	-1.04
A_51_P386539	Rnf125,4930553F04Rik	RIKEN cDNA 4930553F04 gene	9.89E-11	1.40	2.00E-05	1.69	3.54E-01	-1.09
A_51_P365859	Ankrd43,RP23-188H3.6	hypothetical protein A830006N08	3.42E-06	1.25	3.29E-22	1.51	2.01E-01	1.10
A_51_P368586	Ablim1,Limab1,abLIM-L	Mus musculus actin-binding double zinc fing	1.48E-03	1.11	1.20E-04	-1.75	9.32E-01	-1.01
A_52_P296095	Ptprk,AI853699	Mus musculus 2 days neonate sympathetic g	3.45E-01	1.07	2.62E-06	1.84	4.58E-01	-1.07
A_52_P405556	Vipr2,Vip2,VPAC2,VPAC	Bmh1 transcript	5.00E-05	1.62	1.00E+00	1.00	5.48E-01	1.17
A_52_P1027961	B230311A21	hypothetical protein B230311A21	1.62E-02	-1.26	1.25E-15	-2.38	5.43E-01	-1.04
A_52_P244974	Cab39,MO25,39kDa,C7	Calcium binding protein 39, mRNA (cDNA clo	3.00E-05	1.57	1.11E-01	-1.95	3.88E-01	1.16
A_52_P723223	Phf20l1,CGI-72,E13011	PHD finger protein 20-like 1, mRNA (cDNA cl	1.05E-02	1.25	2.60E-04	1.86	6.99E-01	1.07
A_51_P308397	1700047K16Rik	Zinc finger protein 672, mRNA (cDNA clone M	1.23E-02	1.29	1.10E-10	1.95	7.40E-01	-1.03
A_52_P40695	Fut9,mFUT9,AI746471,	fucosyltransferase 9	6.00E-05	1.27	2.06E-02	1.52	8.48E-03	-1.13
A_51_P298688	5330408N05Rik,KIAA1	RIKEN cDNA 5330408N05 gene, mRNA (cDN/	2.73E-01	-1.08	4.13E-15	-7.77	8.87E-01	1.03
A_51_P517878	Nkx2-9,Nkx2.9,tinman,I	NK2 transcription factor related, locus 9 (Dro	1.22E-03	-1.52	3.15E-03	-1.89	1.60E-09	-1.33
A_52_P924229	Mertk,Eyk,Mer,Nyk	C-mer proto-oncogene tyrosine kinase (Mert	3.85E-13	1.64	1.70E-18	1.66	2.57E-01	1.08
A_51_P455090	Cps1,CPS,D1Ucla3,4732	Carbamoyl-phosphate synthetase 1, mRNA ((	3.33E-01	-1.07	5.87E-08	-8.24	8.16E-02	4.30
A_52_P553362	Mamdc1,Mdga2,67204	Adult male olfactory brain cDNA, RIKEN full-l	2.53E-02	1.24	5.42E-09	1.63	8.44E-01	-1.04
A_52_P679711	4930538K18Rik	RIKEN cDNA 4930538K18 gene (4930538K18	9.90E-01	-1.00	1.39E-12	1.60	2.00E-01	-1.08
[illegible]	[illegible]	[illegible]	[illegible]	[illegible]	[illegible]	[illegible]	[illegible]	[illegible]

A_52_P129140	A430088C08Rik	Polymerase (DNA directed), beta (Polb), mRN	1.64E-20	-1.56	7.19E-01	-1.08	4.04E-01	-1.12
A_51_P456721	Azgp1	alpha-2-glycoprotein 1, zinc	5.99E-01	-1.04	6.11E-39	-5.53	1.38E-01	1.49
A_51_P136390	Zfp352,2czf48	zinc finger protein 352	6.80E-01	-1.03	1.03E-08	-2.60	6.63E-01	-1.06
A_52_P691933	AK087708	Mus musculus 2 days pregnant adult female	2.78E-07	1.60	4.35E-03	-1.28	7.89E-01	1.03
A_51_P474701	Fbp1,Fbp2,Fbp-2,FBPas	fructose bisphosphatase 1	4.28E-01	1.07	6.18E-43	-3.35	7.76E-02	-1.23
A_52_P561161	D030011O10Rik,93301	RIKEN cDNA D030011O10 gene	9.69E-03	1.28	2.91E-09	1.65	2.58E-01	-1.06
A_51_P222125	AK053200	0 day neonate lung cDNA, RIKEN full-length	4.61E-15	1.59	1.00E+00	1.00	3.23E-01	1.14
A_52_P235929	Hipk1,Myak,1110062K	homeodomain interacting protein kinase 1	6.25E-01	1.04	6.19E-13	1.58	5.26E-01	-1.06
A_52_P1075623	AK037052	Mus musculus adult female vagina cDNA, RIK	5.39E-01	1.10	1.33E-12	1.87	3.19E-01	1.15
A_52_P923720	AK040434	Adult male corpora quadrigemina cDNA, RIKI	5.24E-07	1.55	1.02E-01	1.69	2.25E-01	1.16
A_51_P174961	F10,fX,Cf10	coagulation factor X	6.45E-02	2.17	9.37E-08	-3.28	7.46E-03	3.59
A_52_P54297	Rbm27,Psc1,AI043120,	MKIAA1311 protein	1.63E-01	1.07	1.00E-05	1.60	7.96E-01	-1.04
A_52_P89567	Rhob,Arh6,Arhb,AA017	ras homolog gene family, member B	1.05E-03	1.23	1.84E-12	1.54	9.91E-01	1.00
A_51_P161207	Rfx3,MRFX3,C230093O	regulatory factor X, 3 (influences HLA class II	3.62E-11	1.61	3.00E-02	1.42	8.73E-01	-1.01
A_52_P212025	Ror1,mRor1,Ntrkr1,281	Receptor tyrosine kinase-like orphan recepto	4.56E-01	1.06	2.37E-06	1.77	8.35E-01	1.02
A_52_P690871	Lphn3,LEC3,CIRL-3,Gm	Latrophilin 3, mRNA (cDNA clone IMAGE:305	1.00E-05	1.54	7.68E-01	1.03	2.33E-01	-1.13
A_51_P242446	Zfp31,MGC30798,C130	zinc finger protein 31	3.47E-12	-1.54	2.90E-01	-1.18	3.92E-02	-1.16
A_51_P376127	Aire,MGC123374,MGC	autoimmune regulator (autoimmune polyen	2.95E-01	-1.10	4.33E-13	2.88	1.87E-02	1.47
A_51_P412200	Klf9,Bteb1,BTEB-1,AA5	basic transcription element binding protein 1	2.97E-02	1.17	2.00E-05	1.94	1.64E-01	1.09
A_51_P417074	3110043J09Rik,AI4308	RIKEN cDNA 3110043J09 gene	8.20E-01	1.01	2.61E-10	1.56	9.68E-01	-1.00
A_52_P302433	Plau,uPA,u-PA	plasminogen activator, urokinase	9.98E-02	-1.18	9.72E-09	-2.55	1.72E-02	1.18
A_52_P664853	Csf2rb1,Bc,Il3r,AIC2B,Il	colony stimulating factor 2 receptor, beta 1,	8.02E-02	-1.39	1.92E-14	-2.00	9.61E-01	-1.02
A_51_P438039	1700063I17Rik	RIKEN cDNA 1700063I17 gene	8.33E-01	1.09	2.93E-06	-5.29	6.01E-01	1.20
A_52_P471482	4631416L12Rik	RIKEN cDNA 4631416L12 gene	5.33E-01	1.04	2.60E-10	2.06	6.57E-01	1.04
A_52_P180692	A630055F16Rik	Mitochondrial ribosomal protein L27, mRNA	7.01E-09	1.73	2.48E-01	1.91	9.91E-01	-1.00
A_52_P938973	C030038I04Rik	Mus musculus adult male corpus striatum cD	1.87E-02	-1.27	2.32E-06	-2.36	1.88E-01	1.14
A_52_P669035	Clca1,Cacc	chloride channel calcium activated 1	5.13E-01	1.16	1.13E-11	1.99	2.36E-06	-1.31
A_52_P1020088	Cntnap2,Caspr2,mKIAA	Contactin associated protein-like 2, transcrip	3.35E-15	2.47	1.00E+00	1.00	1.00E+00	1.00
A_51_P153581	4933424F23Rik	PREDICTED: hypothetical protein LOC71083	3.54E-01	-1.09	2.60E-04	-1.51	7.35E-01	1.13
A_52_P11351	AK048052	Mus musculus 16 days embryo head cDNA, F	7.81E-19	1.68	3.69E-03	1.87	3.86E-01	1.20
A_51_P394115	Aadac,Aada,AI265437,5	arylacetamide deacetylase (esterase)	1.78E-01	1.39	1.84E-33	-11.31	3.00E-05	22.41
A_51_P191433	9530051E23Rik	Adult male urinary bladder cDNA, RIKEN full-	3.72E-01	1.06	3.96E-10	1.63	9.97E-01	1.00
A_52_P589112	Zcchc8,5730565F05Rik	RIKEN cDNA 5730565F05 gene	6.07E-02	1.12	2.00E-05	1.66	2.77E-01	-1.07
A_52_P560996	Tep1,Tp1,MGC91078	Transcribed locus, moderately similar to XP_	2.64E-01	-1.14	4.00E-05	1.88	8.61E-01	1.02
A_52_P139788	Adamtsl1,punctin-1,67	ADAMTS-like 1, mRNA (cDNA clone MGC:736	9.96E-03	1.24	9.84E-11	-1.69	9.18E-01	1.01
A_52_P575646	TC976480	ROD_HUMAN Heterogeneous nuclear ribonu	2.90E-17	-2.46	1.22E-02	-1.67	2.14E-01	-1.09
A_52_P116192	Aff4,Alf4,MCEF,Laf4l,AF	lymphoid nuclear protein related to AF4-like	5.00E-01	-1.04	4.93E-08	-1.54	6.26E-01	1.03
A_51_P336833	Fabp4,Ap2,Lbpl,ALBP/A	fatty acid binding protein 4, adipocyte	4.88E-01	1.08	2.30E-06	1.64	1.46E-01	-1.27
A_52_P129665	Fhl2,SLIM3,C76204	Four and a half LIM domains 2, mRNA (cDNA	4.55E-06	-1.87	5.55E-01	-1.24	2.09E-01	1.12
A_51_P402760	Pla2g4a,cPLA2,Pla2g4	phospholipase A2, group IVA (cytosolic, calci	2.71E-01	1.06	4.72E-12	-1.70	1.00E-01	-1.13
A_52_P71624	Olfr1410,MOR208-2,M	Mus musculus olfactory receptor MOR208-2	2.02E-01	-1.53	2.41E-08	-1.90	9.48E-01	1.04
A_52_P198435	Rasgrp3,Gm327,BC066	Mus musculus RAS, guanyl releasing protein	2.61E-02	-1.13	1.75E-08	-1.89	8.20E-02	-1.11
A_51_P487555	Cd7	CD7 antigen	6.79E-01	-1.03	2.10E-10	1.62	3.89E-02	1.19
A_52_P488572	Elmo1,CED-12,AI17300	engulfment and cell motility 1, ced-12 homol	1.54E-03	1.15	7.70E-13	1.90	6.60E-01	1.03
A_52_P90265	Ucp2,Slc25a8	uncoupling protein 2, mitochondrial	9.37E-02	1.12	8.01E-17	1.87	3.54E-01	-1.06
A_52_P239269	Xrn1,exo,Dhm2,mXrn1	5'-3' exoribonuclease 1 (Xrn1), mRNA	4.25E-02	-1.14	1.00E-04	-2.36	1.00E-04	1.24
A_52_P1180111	AI851790,TAFA2,Tafa-2	Expressed sequence AI851790 (AI851790), m	1.46E-30	2.35	6.35E-01	-1.20	3.79E-01	1.24
A_52_P622850	Hes5	hairy and enhancer of split 5 (Drosophila)	6.62E-19	-2.08	1.86E-01	-1.23	6.70E-01	-1.02
A_52_P46389	Dhx9,RHA,Ddx9,NDHII,I	DEAH (Asp-Glu-Ala-His) box polypeptide 9 (D	1.70E-07	1.50	7.40E-01	-1.15	9.67E-01	1.02
A_52_P342860	Es1,Ee-1,Es-1,Es-4,Es-N	esterase 1	5.54E-01	-1.16	0.00E+00	-37.91	2.38E-07	16.25
A_52_P683957	Arcn1,MGC27546,MGC	archain 1	5.69E-01	1.04	7.00E-05	1.77	7.50E-04	-1.25
A_51_P172502	Cxcl12,PBSF,Sdf1,TLSF,	chemokine (C-X-C motif) ligand 12	1.07E-02	-1.16	0.00E+00	-2.26	3.82E-01	1.05
A_52_P518613	Adam23,MDC3	Mus musculus 9 days embryo whole body cD	1.98E-11	2.10	1.00E+00	1.00	1.00E+00	1.00
A_51_P117369	Phf8,mKIAA1111,98301	RIKEN cDNA 9830141C09 gene	2.26E-07	1.65	6.08E-01	1.16	2.83E-01	1.15
A_52_P375918	1500010J02Rik,RP23-1	Mus musculus adult male thymus cDNA, RIKI	2.00E-05	1.94	1.00E+00	1.00	4.24E-01	1.37
A_52_P461777	Pcdh9,Gm297,Gm1213	PREDICTED: similar to protocadherin 9 [Mus	3.36E-03	1.45	6.90E-16	2.00	3.21E-01	-1.19
A_52_P13389	Myct1,Mtmc1,AI22594	myc target 1	2.67E-06	-1.58	1.24E-02	-2.06	1.94E-01	1.14
A_52_P1140316	AK048885	Mus musculus 0 day neonate cerebellum cDI	4.70E-04	1.61	1.00E+00	1.00	4.26E-01	1.26
A_52_P128906	2210018M03Rik	RIKEN cDNA 2210018M03 gene, mRNA (cDN	8.86E-03	1.23	1.31E-08	1.60	4.32E-01	-1.04
A_52_P1044190	Lphn3,LEC3,CIRL-3,Gm	Latrophilin 3, mRNA (cDNA clone IMAGE:305	1.27E-06	1.92	4.48E-01	1.35	4.68E-01	1.28
A_52_P573255	Cdc42ep1,CEP1,Borg5,I	CDC42 effector protein (Rho GTPase binding	4.32E-01	-1.05	2.02E-13	-1.90	2.27E-02	1.08
A_51_P381060	Pilrb,Fdact,FDFACT,Pilrl	paired immunoglobin-like type 2 receptor be	9.93E-02	1.32	3.97E-08	2.28	8.26E-03	-1.43
A_52_P536000	Rnf44,AI854545,mKIAA	Ring finger protein 44, mRNA (cDNA clone M	1.12E-01	1.16	6.09E-08	1.55	7.07E-02	1.24
A_51_P406846	9530066K23Rik,RP23-2	RIKEN cDNA 9530066K23 gene	6.22E-07	-1.38	2.53E-02	-1.54	1.61E-01	-1.11
A_51_P159284	D030060M11Rik,AV30	RIKEN cDNA D030060M11 gene	5.44E-01	-1.03	5.92E-10	-1.91	5.95E-01	1.03
A_52_P371401	Sbf2,Mtmr13,mMTMH	Myotubularin related protein 13, mRNA (cDN	1.07E-08	1.49	4.28E-02	1.81	1.71E-01	1.09
A_52_P412362	Olfr788,MOR111-4	Mus musculus olfactory receptor MOR111-4	3.28E-01	-1.07	5.08E-07	-1.73	7.41E-01	1.03
A_52_P770923	Centb2,ACAP2,CNT-B2,	PREDICTED: Mus musculus centaurin, beta 2	1.33E-23	1.59	9.41E-02	1.31	1.30E-01	1.21
A_52_P923671	Odz2,Odz3,Ten-m2,mK	ODZ2 (Odz2)	2.00E-05	1.69	8.91E-01	1.03	4.82E-01	1.14
A_51_P293982	AI840980,MGC30448	expressed sequence AI840980	4.18E-01	-1.07	3.93E-14	-1.81	5.13E-01	-1.06
A_52_P427265	Anapc1,Apc1,Mcpr,tsg	Mus musculus 0 day neonate eyeball cDNA, I	5.48E-17	1.68	5.35E-01	1.20	5.16E-01	1.09
A_52_P160057	2610042L04Rik	RIKEN cDNA 2610042L04 gene (2610042L04I	1.61E-01	1.10	7.70E-15	1.59	6.69E-01	-1.02
A_51_P127695	Greb1,AF180470,AU02	gene regulated by estrogen in breast cancer	3.12E-01	1.10	2.90E-04	2.12	9.68E-01	-1.01
A_51_P213359	Has2	hyaluronan synthase 2	5.63E-14	-1.68	9.12E-01	1.03	1.60E-03	-1.29
A_51_P502614	Dusp6,MKP3,MKP-3,PY	dual specificity phosphatase 6	5.09E-02	-1.12	4.51E-27	-1.59	3.85E-01	-1.12
A_51_P212782	Il1b,Il-1b,IL-1beta	interleukin 1 beta	1.99E-01	1.14	1.80E-04	-1.66	1.24E-01	1.10
A_52_P204035	4933412A08Rik	DNA segment, Chr 19, ERATO Doi 737, expre	1.00E+00	1.00	2.20E-09	-8.17	1.00E+00	1.00
[illegible]	[illegible]	[illegible]	[illegible]	[illegible]	[illegible]	[illegible]	[illegible]	[illegible]

A_52_P1107923	Garnl3,AW120551	GTPase activating RANGAP domain-like 3 (Ga	1.43E-22	1.63	5.16E-03	1.44	7.74E-06	1.24
A_52_P495829	Pax7A	Mus musculus transcriptional factor PAX7A (	3.34E-01	-1.53	5.93E-01	-1.26	3.18E-06	2.46
A_52_P302371	Kcng3,KV6.3,Kv10.1a,K\	potassium voltage-gated channel, subfamily	5.46E-02	-1.14	4.66E-08	-2.45	3.88E-01	1.13
A_51_P460332	Apoc4,Acl	apolipoprotein C-IV	6.02E-01	-1.22	3.61E-14	-5.74	2.00E-05	4.15
A_51_P289423	NAP057047-1	Unknown	8.71E-01	-1.01	3.83E-08	1.71	8.50E-04	-1.19
A_52_P615096	Acox1,AOX,AI042784,D	acyl-Coenzyme A oxidase 1, palmitoyl	4.37E-01	1.07	8.00E-05	1.54	2.77E-02	-1.21
A_52_P212473	C730049O14Rik	0 day neonate thymus cDNA, RIKEN full-leng	1.97E-06	1.37	2.05E-23	1.81	8.51E-01	1.02
A_52_P374193	TC1002442	AB016424 rbm3 {Mus musculus}, partial (95	4.68E-08	-1.55	1.98E-02	-1.40	8.98E-01	1.01
A_51_P503494	Arc,Arc3.1,C86064,arg 3	activity regulated cytoskeletal-associated pro	4.21E-31	-2.06	6.78E-08	-2.36	2.56E-01	-1.19
A_51_P153124	Emcn,Muc14,AI315669	endomucin	1.81E-01	-1.06	6.04E-23	-1.62	6.74E-01	-1.02
A_51_P357182	Mapk8,JNK,JNK1,Prkm8	mitogen activated protein kinase 8	2.02E-01	1.05	7.00E-05	1.64	1.85E-01	1.10
A_52_P208710	Ubap2l,C77168,Nice-4,I	Ubiquitin associated protein 2-like (Ubap2l),	8.92E-41	-1.99	3.55E-01	-1.27	2.93E-01	-1.13
A_52_P375312	Amica1,AMICA,Crea7,G	Dendritic-cell specific protein Crea7 (Crea7)	3.30E-01	1.07	8.82E-06	1.54	8.02E-01	1.02
A_51_P227392	Rhou,Arhu,G28K,WRCH	ras homolog gene family, member U	1.44E-01	1.09	3.34E-06	2.00	3.07E-01	1.05
A_52_P747383	A330084C13Rik,Gm956	PREDICTED: Mus musculus RIKEN cDNA A330	1.18E-08	1.39	5.00E-05	1.57	9.77E-01	-1.00
A_52_P60194	C4bp,AI195242	complement component 4 binding protein	3.81E-01	-1.16	5.12E-39	-4.28	4.83E-03	3.48
A_52_P5823	B230312I18Rik,mszf70,	RIKEN cDNA B230312I18 gene, mRNA (cDNA	5.00E-05	1.34	1.15E-02	1.60	2.16E-01	1.13
A_52_P297176	E230001N04Rik	RIKEN cDNA E230001N04 gene	5.95E-42	2.34	2.06E-09	2.68	1.61E-01	1.16
A_52_P387467	XM_143807	Mus musculus similar to alpha-2u-globulin IV	7.56E-01	-1.05	1.20E-22	-41.77	2.56E-01	2.82
A_52_P217875	H3f3b,H3.3B,9430068D	H3 histone, family 3B, mRNA (cDNA clone M	8.02E-07	1.45	8.16E-06	1.53	2.46E-01	-1.13
A_52_P273438	NAP108202-1	Unknown	3.45E-02	-1.23	7.78E-08	2.00	7.47E-02	-1.27
A_52_P249514	Ccl12,MCP-5,Scya12	chemokine (C-C motif) ligand 12	8.25E-01	1.02	3.43E-18	-1.83	4.15E-02	-1.11
A_52_P63343	TC1024234	AY221172 p67-like superoxide-generating NA	5.38E-01	1.10	2.46E-09	2.16	6.63E-02	-1.13
A_52_P54609	Eml1,EMAP,ELP79,EMA	PREDICTED: echinoderm microtubule associa	4.08E-02	1.24	2.32E-06	-1.51	4.80E-04	1.26
A_52_P625594	Becn1,MGC6843,49215	Mus musculus adult male olfactory brain cDN	8.73E-01	1.02	1.32E-10	2.20	3.74E-02	1.09
A_52_P260555	Vil1,Vil	villin	3.42E-02	1.11	1.17E-14	-1.60	8.30E-01	1.02
A_52_P250106	Ank3,AnkG,Ank-3,MGC	Ankyrin 3, epithelial (Ank3), transcript varian	2.05E-13	1.78	6.61E-01	-1.15	2.26E-01	1.29
A_51_P225427	Pkp2,AA516617,12000(	RIKEN cDNA 1200008D14 gene	5.93E-01	-1.03	7.98E-17	1.57	3.92E-01	1.05
A_51_P160592	4930488L21Rik,493045	Mus musculus adult male testis cDNA, RIKEN	9.07E-01	1.01	3.00E-05	1.51	2.57E-01	-1.07
A_51_P295118	Ppp1r9a,BB181831,mKI	Mus musculus 10, 11 days embryo whole bo	1.70E-04	1.35	1.52E-03	1.75	4.23E-01	-1.23
A_52_P707807	4833420G17Rik,C85193	RIKEN cDNA 4833420G17 gene, mRNA (cDNA	3.26E-03	1.25	4.87E-16	2.20	2.00E-01	-1.14
A_52_P292693	Hnrpdl,JKTBP,AA40743	heterogeneous nuclear ribonucleoprotein D-	3.03E-01	1.09	2.82E-09	1.57	4.05E-02	-1.19
A_51_P248230	Trpv3,AI644701,MGC12	transient receptor potential cation channel, s	6.22E-01	-1.25	3.30E-01	-1.58	3.70E-04	2.78
A_51_P240977	4930482G09Rik	Adult male testis cDNA, RIKEN full-length enr	2.30E-04	2.26	1.00E+00	1.00	2.62E-01	1.54
A_52_P536494	Mycn,Nmyc,N-myc,Nm	neuroblastoma myc-related oncogene 1	1.43E-43	-1.60	3.94E-07	-1.96	6.14E-01	-1.06
A_52_P282905	LOC382044	similar to Es1 protein	8.10E-01	-1.10	2.72E-01	-1.99	4.00E-05	6.02
A_52_P114340	NAP103495-1	Unknown	2.71E-01	-1.06	1.59E-08	1.52	4.86E-01	-1.05
A_51_P125986	Gan,gigaxonin,A330045	PREDICTED: similar to gigaxonin [Mus muscu	2.70E-02	1.15	9.00E-05	1.90	6.70E-01	1.05
A_52_P651425	Ncf1,NOXO2,Ncf-1,p47	neutrophil cytosolic factor 1	4.69E-01	1.03	1.34E-09	1.54	7.79E-01	-1.02
A_52_P787239	AK042793	7 days neonate cerebellum cDNA, RIKEN full-	2.21E-07	1.56	5.84E-02	1.25	7.90E-01	-1.04
A_51_P396417	8030450B20Rik	Mus musculus 15 days embryo male testis cD	8.00E-05	2.19	1.00E+00	1.00	1.00E+00	1.00
A_52_P1179775	Mtap2,MAP2,Mtap-2	Mus musculus adult male corpus striatum cD	8.37E-07	1.34	7.99E-03	2.02	9.66E-01	-1.01
A_51_P393668	Tm6sf2	transmembrane 6 superfamily member 2	4.17E-03	-1.23	1.82E-06	-1.54	7.56E-01	1.02
A_51_P489996	Dst,ah,dt,Bpag,BP230,E	dystonin	5.84E-20	1.52	6.19E-01	-1.08	9.26E-01	1.01
A_52_P1060359	Mrpl15,Rpml7,MRP-L7,	Mitochondrial ribosomal protein L15, mRNA	1.20E-04	1.57	3.31E-02	2.05	2.27E-01	1.29
A_52_P599264	Mdfic,Kdt1,Mdfid	kidney cell line derived transcript 1	1.99E-02	-1.19	2.02E-07	-1.53	5.92E-01	1.02
A_52_P874953	4833408G04Rik	Mus musculus 0 day neonate head cDNA, RIK	3.60E-09	1.71	1.07E-01	1.59	2.69E-01	1.28
A_51_P123879	Srd5a2l2,D330017N19F	steroid 5 alpha-reductase 2-like 2	1.75E-03	-1.79	8.32E-13	3.50	7.14E-03	-1.42
A_51_P193146	Ms4a6c,2200009H22Ri	membrane-spanning 4-domains, subfamily A	3.92E-06	1.20	5.50E-07	1.85	8.03E-01	1.02
A_52_P621357	TC1067460	S25058 Ig kappa chain - mouse, partial (86%)	1.57E-02	-1.31	4.08E-01	1.59	2.70E-04	-2.40
A_52_P443544	D330012F22Rik	Mus musculus RIKEN cDNA D330012F22 gen	7.85E-09	1.25	1.87E-08	1.62	8.41E-01	-1.03
A_51_P149112	Lphn3,LEC3,CIRL-3,Gm1	Latrophilin 3, mRNA (cDNA clone IMAGE:305	7.95E-07	1.68	8.96E-01	1.02	1.46E-02	1.63
A_51_P382331	4632413K17Rik,Os-9,AI	Mus musculus 0 day neonate skin cDNA, RIKI	7.00E-05	1.55	3.58E-22	2.40	9.93E-01	1.00
A_51_P518014	Slc36a3,PAT3,TRAMD2,	solute carrier family 36 (proton/amino acid s	4.11E-01	-1.40	1.74E-13	-7.76	9.31E-01	-1.05
A_52_P208703	Ubap2l,C77168,Nice-4,I	Ubiquitin associated protein 2-like (Ubap2l),	1.43E-23	-1.85	1.40E-01	-1.43	6.15E-03	-1.20
A_52_P549427	Mid1,Fxy,61B3-R,Trim1	Midline 1 (Mid1), mRNA	4.61E-01	1.13	1.16E-07	-1.60	1.57E-03	1.81
A_51_P470542	Apob,apob-48,AI31505	Mus musculus apolipoprotein B (Apob), mRN	8.79E-01	1.10	2.24E-38	-14.45	1.37E-08	9.22
A_52_P629895	Adh1,Adh-1,ADH-AA,Ac	alcohol dehydrogenase 1 (class I)	7.55E-01	1.06	8.84E-26	-2.81	3.09E-01	-1.06
A_52_P120842	Man1a2,PCR2,Man1b,A	mannosidase 1, beta	6.63E-02	1.07	1.59E-13	1.79	5.60E-01	1.09
A_51_P231184	Anpep,Apn,Cd13,Lap1,p	alanyl (membrane) aminopeptidase	5.37E-01	-1.03	1.58E-07	-1.57	1.11E-02	1.16
A_52_P1036791	AK080404	7 days neonate cerebellum cDNA, RIKEN full-	1.57E-09	1.88	5.00E-05	1.73	3.92E-01	1.10
A_52_P227746	NAP070876-1	Unknown	4.94E-02	1.17	2.38E-16	1.67	2.94E-02	-1.16
A_52_P337899	Fkbp5,Dit1,51kDa,FKBP	FK506 binding protein 5	3.11E-01	1.08	1.10E-37	2.59	5.83E-03	1.12
A_52_P547740	Usp8,Ubpy,AI574262,A	Ubiquitin specific peptidase 8, mRNA (cDNA (	4.75E-07	1.30	2.00E-05	1.52	4.33E-01	1.13
A_52_P151198	Trip11,AI450776,26105	Thyroid hormone receptor interactor 11, mR	3.01E-13	1.81	9.78E-01	-1.01	8.98E-02	-1.25
A_51_P229911	Adcy4,KIAA4004,mKIAA	adenylate cyclase 4	3.74E-01	1.05	5.24E-09	-1.79	1.02E-01	1.16
A_52_P537438	Mapk10,JNK3,Serk2,JNI	Mus musculus 0 day neonate cerebellum cDI	3.20E-09	1.73	4.11E-01	1.34	1.45E-01	1.12
A_52_P420504	0610041G09Rik	RIKEN cDNA 0610041G09 gene	2.42E-02	1.15	1.99E-08	-1.50	8.70E-04	1.26
A_52_P311300	1700080O16Rik,MGC13	PREDICTED: RIKEN cDNA 1700080O16 [Mus	7.96E-01	1.16	1.03E-01	-2.44	4.80E-04	4.51
A_52_P771744	Cdk8,MGC37111	Cyclin-dependent kinase 8 (Cdk8), transcript	6.00E-02	-1.28	1.31E-07	-1.82	2.21E-01	1.17
A_52_P987357	6720470G18Rik	PREDICTED: similar to hydroxyproline-rich gl	1.04E-06	1.34	5.30E-04	1.64	4.00E-01	1.10
A_51_P436521	Tmc2,CWEA2	transmembrane channel-like gene family 2	3.19E-01	-1.22	5.68E-17	-1.86	9.19E-01	-1.05
A_51_P256427	BC042720,C230061B10	MKIAA1843 protein	1.20E-02	1.13	4.55E-39	-1.52	6.22E-01	1.08
A_51_P107752	E430014L09Rik	16 days embryo lung cDNA, RIKEN full-length	2.21E-07	1.54	4.07E-02	1.19	8.96E-03	1.17
A_51_P273679	Dmgdh,AI787269,MGC	RIKEN cDNA 1200014D15 gene	9.91E-02	-1.19	3.00E-05	-1.62	2.63E-01	1.33
A_52_P96542	4930518F03Rik	RIKEN cDNA 4930518F03 gene	1.50E-04	1.21	1.00E-05	1.74	2.46E-01	-1.29
A_52_P481493	Fkbp5,Dit1,51kDa,FKBP	FK506 binding protein 5	3.62E-11	1.55	4.37E-08	3.07	7.68E-01	1.03
[illegible]	[illegible]	[illegible]	[illegible]	[illegible]	[illegible]	[illegible]	[illegible]	[illegible]

A_52_P517029	Tmeff2,4832418D20Rik	transmembrane protein with EGF-like and tw	8.54E-07	1.34	1.00E-05	1.50	1.22E-01	-1.18
A_52_P779355	Sec14l2,TAP,1300013M	SEC14-like 2 (S. cerevisiae) (Sec14l2), mRNA	6.00E-05	1.47	7.38E-07	3.52	8.34E-01	-1.08
A_52_P10732	Mtap6,STOP,F-STOP,28	Microtubule-associated protein 6 (Mtap6), m	5.13E-01	-1.05	8.58E-06	-1.52	9.56E-01	1.00
A_52_P883277	Ncald,AI848120,D15Ert	Neurocalcin delta, mRNA (cDNA clone MGC:3	9.56E-02	-1.20	1.35E-01	-1.53	4.20E-04	1.37
A_51_P108226	1100001G20Rik	RIKEN cDNA 1100001G20 gene	8.72E-01	1.04	0.00E+00	-5.66	7.33E-06	-2.64
A_51_P130374	Tek,Hyk,Tie2,Cd202b	endothelial-specific receptor tyrosine kinase	4.14E-01	1.03	2.88E-08	-1.77	8.88E-06	1.18
A_52_P600518	Tsc22d3,DIP,Gilz,Dsip1,	glucocorticoid-induced leucine zipper	7.63E-01	1.02	1.21E-11	1.52	9.34E-01	-1.01
A_51_P508959	E130201N16Rik,G90	RIKEN cDNA E130201N16 gene	7.03E-02	1.24	1.30E-04	1.70	8.34E-02	1.09
A_52_P963507	D14Ertd171e,CAST,643	DNA segment, Chr 14, ERATO Doi 171, expre	2.45E-12	1.72	7.05E-01	-1.06	2.06E-02	1.33
A_51_P232558	4933414I06Rik	Adult male testis cDNA, RIKEN full-length enr	7.49E-01	1.07	6.09E-15	2.97	1.38E-03	-1.23
A_51_P181599	LOC545728	similar to Speer1-ps1 protein	1.89E-01	1.11	2.73E-12	-4.41	6.73E-01	1.15
A_52_P980494	Ncald,AI848120,D15Ert	Neurocalcin delta, mRNA (cDNA clone MGC:3	1.19E-11	2.04	2.05E-01	1.15	8.00E-01	1.09
A_52_P360413	Ankra2,Ankra,AI450635	Ankyrin repeat, family A (RFXANK-like), 2, mF	4.00E-05	-1.52	8.18E-01	-1.06	6.34E-01	-1.03
A_52_P612636	Gja5,Cx40,Cnx40,Gja-5,	Zinc finger protein 697, mRNA (cDNA clone M	9.29E-01	-1.01	1.68E-16	1.73	6.75E-02	1.08
A_52_P324042	Serpina1b,PI2,Dom2,Sp	serine (or cysteine) proteinase inhibitor, clad	6.21E-01	-1.19	4.68E-21	-9.42	5.90E-04	4.61
A_52_P660648	Pla2g4a,cPLA2,Pla2g4	phospholipase A2, group IVA (cytosolic, calci	6.57E-01	1.03	1.84E-08	-1.66	1.75E-01	-1.09
A_51_P413782	Arhgef17,AI428794,AW	MKIAA0337 protein	2.47E-13	1.57	1.00E+00	1.00	3.05E-01	1.40
A_51_P312052	AK053398	Mus musculus 0 day neonate eyeball cDNA, I	2.58E-15	1.59	1.00E+00	1.00	2.84E-01	1.44
A_52_P100391	Nalp9a,D7Ertd565e,Nal	NALP9A	5.11E-02	-1.32	1.00E-05	-1.83	2.54E-01	1.17
A_52_P249965	Xdh,Xor,Xox1,Xox-1	xanthine dehydrogenase	9.30E-01	-1.01	2.11E-41	2.63	8.14E-01	-1.02
A_52_P482071	9330133O14Rik	RIKEN cDNA 9330133O14 gene	4.73E-08	1.31	6.00E-05	1.84	5.27E-01	-1.08
A_51_P455681	Cd3e,CD3,T3e,AI50478	CD3 antigen, epsilon polypeptide	2.42E-01	-1.26	5.41E-08	-1.57	9.87E-01	-1.01
A_52_P676510	Tgtp,Gtp2,Mg21	T-cell specific GTPase	7.32E-07	-1.53	3.62E-15	-1.85	5.20E-08	-1.29
A_51_P450007	5730585A16Rik	8 days embryo whole body cDNA, RIKEN full-	8.30E-02	-1.28	1.44E-07	-2.00	3.06E-01	1.14
A_51_P430423	Ada	adenosine deaminase	1.33E-11	1.81	0.00E+00	5.41	7.69E-01	1.05
A_51_P345393	Fas,lpr,APT1,CD95,APO	tumor necrosis factor receptor superfamily, r	9.20E-01	1.01	9.54E-25	1.58	9.09E-02	-1.14
A_51_P309988	Gprc5c,1110028I06Rik,	G protein-coupled receptor, family C, group !	3.02E-09	-1.61	5.01E-02	-1.31	1.90E-02	-1.17
A_51_P326826	Mela,Ag,env,gag,pol,80	melanoma antigen	5.32E-01	-1.07	8.35E-12	1.62	2.53E-01	-1.17
A_52_P666011	Tex27,TEG-27,AW5392	Testis expressed gene 27 (Tex27), mRNA	2.89E-07	1.72	5.33E-01	-1.32	1.00E+00	1.00
A_52_P537887	TC1059850	AF369902 MTO1 {Mus musculus}, partial (13	2.00E-05	1.24	3.00E-05	1.58	3.62E-01	-1.04
A_51_P108459	Gpr65,Dig1,TDAG8,Gpc	G-protein coupled receptor 25	5.36E-11	1.88	1.90E-04	2.57	4.33E-01	1.09
A_52_P5491	Frem2,my,nv1,603044C	Fras1 related extracellular matrix 2 (Frem2 ge	2.64E-01	-1.07	8.00E-05	-1.84	9.55E-01	1.00
A_52_P1164065	AK046743	Mus musculus adult male cortex cDNA, RIKEI	2.36E-11	1.69	2.85E-03	1.41	3.43E-01	1.10
A_51_P506309	Hrg,HPRG,HRGP,D16JH	histidine-rich glycoprotein	9.90E-01	1.00	1.92E-12	-1.89	7.36E-02	1.76
A_52_P875096	Ube2w,6130401J04Rik	RIKEN cDNA 6130401J04 gene, mRNA (cDNA	9.20E-01	1.01	4.36E-07	1.59	5.62E-02	1.85
A_52_P667157	NAP123939-1	Unknown	9.94E-16	-1.58	3.82E-02	1.14	5.55E-03	1.32
A_52_P360241	Prickle2,6230400G14Ri	Prickle-like 2 (Drosophila), mRNA (cDNA clon	9.70E-01	-1.00	1.20E-09	-7.86	1.28E-01	1.24
A_51_P318564	Wnk1,Prkwnk1,mKIAAC	Mus musculus protein kinase, lysine deficien	2.05E-01	1.10	4.36E-07	-1.68	5.93E-01	1.14
A_52_P78333	Lrrc29,FBL9,FBXL9,A63(	B16 F10Y cells cDNA, RIKEN full-length enrich	1.86E-02	1.22	1.69E-06	1.62	7.15E-01	-1.03
A_51_P204247	C8a,MGC29381	complement component 8, alpha polypeptid	4.00E-01	-1.74	5.50E-04	-4.09	9.93E-07	7.28
A_51_P364503	AK036001	Mus musculus 16 days neonate cerebellum c	5.00E-05	1.85	6.34E-01	1.25	7.29E-01	1.10
A_52_P651393	Epha10,A330090H18	hypothetical protein A330090H18	3.59E-02	1.18	4.47E-25	1.65	3.53E-01	-1.11
A_52_P1188470	Usp46,AI451644,11900	Ubiquitin specific peptidase 46 (Usp46), mRN	8.37E-01	-1.02	1.70E-01	-1.62	3.50E-04	1.17
A_52_P469306	Pax6,Dey,Sey,Pax-6,15(	paired box gene 6	7.52E-08	1.44	2.12E-01	1.72	9.65E-01	1.01
A_52_P883283	3110004L20Rik	RIKEN cDNA 3110004L20 gene, mRNA (cDNA	1.10E-01	-1.08	2.17E-08	-1.56	5.10E-01	1.09
A_52_P715852	AK079958	Adult male aorta and vein cDNA, RIKEN full-le	9.77E-01	1.00	2.55E-08	1.75	6.38E-01	1.05
A_51_P236303	Serpina1d,PI4,Dom4,Sp	serine (or cysteine) proteinase inhibitor, clad	4.00E-02	-1.11	6.43E-29	-16.57	2.65E-01	2.10
A_52_P12877	Hspa8,70kDa,Hsc70,Hs	heat shock protein 8	4.99E-02	1.15	2.40E-04	1.74	2.90E-08	-1.29
A_52_P77837	Nt5c3,p36,Nt5y,PN-1,P	5'-nucleotidase, cytosolic III	5.07E-02	1.10	2.71E-07	1.53	2.46E-01	-1.06
A_51_P312121	Xdh,Xor,Xox1,Xox-1	xanthine dehydrogenase	3.26E-01	1.05	3.12E-19	2.29	7.86E-01	1.03
A_52_P67570	Lass4,Trh1,2900019C1	Longevity assurance homolog 4 (S. cerevisiae	1.89E-13	-1.47	4.53E-01	-1.51	5.82E-01	1.09
A_51_P499940	Hmgb2,Hmg2,HMG-2,C	High mobility group box 2, mRNA (cDNA clon	1.00E-05	1.42	4.62E-11	2.04	5.12E-03	-1.15
A_52_P100926	Il1a,Il-1a	interleukin 1 alpha	5.28E-07	-1.66	5.09E-01	-1.34	7.70E-01	1.05
A_52_P220739	9630041I06Rik	Calcium/calmodulin-dependent protein kinas	2.24E-22	1.61	1.57E-01	1.26	8.41E-02	1.19
A_52_P730870	C230004F18Rik	RIKEN cDNA C230004F18 gene (C230004F18	2.67E-03	1.35	7.00E-05	1.62	8.50E-01	1.04
A_51_P509750	Gig1,C030026M03,C03(	glucocorticoid induced gene 1	2.64E-09	1.91	1.00E+00	1.00	6.65E-01	1.29
A_51_P243103	A630006J10Rik	Mus musculus 3 days neonate thymus cDNA,	6.09E-08	1.78	1.00E+00	1.00	1.00E+00	1.00
A_51_P400581	Anp32e,CPD1,LANPL,LA	acidic (leucine-rich) nuclear phosphoprotein	8.81E-01	1.01	5.09E-15	1.73	8.20E-04	-1.17
A_52_P557378	BC019561,MGC28728,/	hypothetical protein MGC28728	1.09E-02	1.17	7.37E-10	1.52	8.20E-04	-1.20
A_51_P517182	Hrasls3,C78643,Hrev10	HRAS like suppressor 3 (Hrasls3), mRNA	2.22E-01	-1.14	7.00E-05	1.97	3.42E-01	-1.12
A_52_P361066	2810426N06Rik,AI2563	RIKEN cDNA 2810426N06 gene	4.63E-01	1.04	2.00E-05	1.52	3.02E-01	-1.08
A_52_P269595	Ube2i,UBC9,Ubce9,Mm	Ubiquitin-conjugating enzyme E2I, mRNA (cD	5.65E-08	1.60	1.13E-01	1.51	3.66E-01	1.04
A_51_P327405	5830443L24Rik	RIKEN cDNA 5830443L24 gene	3.56E-02	-1.20	1.70E-04	-2.25	9.77E-02	-1.29
A_51_P209597	Afm,Alf,MGC118711,al	Afamin (Afm), mRNA	9.19E-01	-1.05	4.00E-05	-8.38	1.00E+00	1.00
A_51_P505974	AK038328	Mus musculus 16 days neonate thymus cDN/	0.00E+00	1.74	2.00E-05	-1.33	9.32E-01	-1.01
A_52_P247086	4930535B03Rik,AL0228	PREDICTED: hypothetical protein LOC75137	3.54E-01	1.17	2.21E-26	2.41	3.30E-04	-1.28
A_52_P997209	Myh9,Myhn1,C80049,D	Myosin, heavy polypeptide 9, non-muscle (M	1.53E-07	-1.39	3.68E-08	-1.53	1.34E-02	-1.10
A_52_P251690	Gvin1,VLIG,Iigs1,VLIG-1	RIKEN cDNA 9130002C22 gene	5.40E-02	-1.14	8.97E-08	-1.90	1.77E-01	-1.18
A_51_P189814	Cldn5,MBEC1,Tmvcf,AI	claudin 5	8.62E-02	-1.13	4.25E-11	-1.91	3.36E-01	-1.05
A_51_P179933	A_51_P179933	Unknown	5.09E-01	-1.10	5.88E-09	-1.67	8.59E-01	1.08
A_51_P188501	AK053596	PREDICTED: Mus musculus hypothetical prot	0.00E+00	1.87	7.76E-01	-1.03	9.69E-01	-1.01
A_52_P1147740	Cxadr,CAR,MCAR,AU01	Coxsackievirus and adenovirus receptor, mRI	1.60E-07	-1.26	1.16E-22	-1.83	2.89E-01	1.14
A_52_P178486	TC1037141	A48094 serum and glucocorticoid-regulated	2.00E-04	1.39	2.45E-01	1.65	2.45E-02	-1.20
A_52_P574668	Nt5e,NT,Nt5,eNT,CD73	5' nucleotidase, ecto	2.32E-01	1.10	2.88E-11	1.71	4.86E-01	-1.03
A_51_P164504	Apoc1	apolipoprotein C-I	1.53E-01	-1.13	2.62E-10	-1.98	5.53E-03	1.22
A_52_P883770	AK053244	Mus musculus 0 day neonate lung cDNA, RIK	6.23E-02	1.96	1.27E-07	3.61	2.62E-01	1.62
A_52_P843410	AK037788	Mus musculus 16 days neonate thymus cDN/	1.51E-01	-1.20	1.15E-01	-1.65	3.33E-06	1.29
[illegible]	[illegible]	[illegible]	[illegible]	[illegible]	[illegible]	[illegible]	[illegible]	[illegible]

A_52_P665604	Adarb1,RED1,ADAR2,A\	Adenosine deaminase, RNA-specific, B1 (Ada	1.00E-03	1.14	2.28E-09	1.52	6.87E-01	1.02
A_51_P230269	H2-Q10,Q10,Qa10,H-2(	histocompatibility 2, Q region locus 10	4.81E-01	-1.07	2.38E-36	-3.19	4.03E-02	1.85
A_52_P458141	D730040F13Rik	Mus musculus 0 day neonate kidney cDNA, F	7.08E-08	1.38	2.10E-01	-1.55	9.62E-01	-1.00
A_51_P269273	Acr,AI323726,MGC124(	preproacrosin	3.93E-07	1.16	8.98E-11	1.54	4.59E-01	-1.06
A_52_P387069	Trpm3,MLSN2,LTRPC3,/	Transient receptor potential cation channel,	3.00E-05	1.47	2.30E-01	1.59	1.51E-02	1.26
A_51_P158734	Ngb	neuroglobin	8.79E-02	-1.33	9.83E-10	-1.53	9.32E-01	1.04
A_52_P681727	Eif2ak1,Hri	Mus musculus adult retina cDNA, RIKEN full-l	4.50E-01	1.10	1.31E-06	1.80	3.22E-01	1.14
A_52_P247472	Txndc13,AI843224,AW(	Mus musculus 15 days embryo male testis cD	7.96E-01	1.03	4.06E-07	2.14	4.06E-01	1.10
A_52_P362917	Pfkfb3,iPFK-2,E330010I	6-phosphofructo-2-kinase/fructose-2,6-biph	4.15E-01	1.05	2.00E-04	2.85	1.31E-01	-1.22
A_51_P154933	Zdhhc7,Gramp2,AL024(	zinc finger, DHHC domain containing 7	1.02E-01	-1.11	2.53E-07	-1.55	3.76E-01	1.32
A_52_P499551	Cugbp2,ETR-3,Napor,C8	CUG triplet repeat, RNA binding protein 2, m	4.38E-11	1.51	8.05E-01	-1.03	2.62E-01	1.18
A_51_P448741	Tnfsf10,TL2,Ly81,Trail,A	tumor necrosis factor (ligand) superfamily, m	2.97E-01	-1.20	3.50E-04	-2.61	5.89E-01	-1.08
A_51_P177371	Prnd,Dpl,PrPLP,doppel,	prion protein dublet	9.97E-07	1.76	8.37E-11	2.25	3.71E-01	1.25
A_51_P258768	Paqr5,AV002411,BB115	Progestin and adipoQ receptor family membe	4.64E-01	1.03	4.37E-09	1.54	7.76E-01	1.04
A_52_P996015	AK032739	12 days embryo male wolffian duct includes	3.00E-04	3.94	1.00E+00	1.00	1.00E+00	1.00
A_52_P367755	Hist2h2aa2,H2a-614	Histone 2, H2aa1 (Hist2h2aa1), mRNA	9.00E-05	1.56	5.61E-10	2.01	8.10E-04	-1.30
A_52_P731018	Rapgef4,Epac2,KIAA404	CAMP-GEFII	1.14E-12	1.89	4.50E-04	1.74	9.38E-01	-1.02
A_52_P876850	Polr2a,220kDa,Rpo2-1	Polymerase (RNA) II (DNA directed) polypept	1.16E-20	1.48	1.04E-11	1.54	6.19E-03	1.15
A_52_P326502	Sox21,Sox25	SRY-box containing gene 21	1.83E-01	-1.08	8.49E-11	-1.74	9.23E-01	1.01
A_52_P433667	Vsx1,CHX10-like	visual system homeobox 1 homolog (zebrafis	2.61E-02	-1.29	1.43E-09	-1.91	6.49E-02	1.14
A_52_P730733	Pde4b,Dpde4,dunce,R7	C57BL/6J phosphodiesterase 4B (Pde4b)	2.69E-06	1.80	3.19E-06	2.54	5.19E-01	1.22
A_51_P108659	Pon1,Pon	paraoxonase 1	2.26E-01	-1.19	0.00E+00	-4.86	2.19E-01	1.97
A_52_P1020860	AA726875	Bone marrow macrophage cDNA, RIKEN full-	6.83E-01	-1.03	6.89E-07	-1.75	6.84E-01	-1.02
A_51_P173678	Slc10a6,Soat,C78479,8	RIKEN cDNA 8430417G17 gene	3.96E-01	1.47	4.00E-05	3.80	1.00E+00	1.00
A_51_P502859	Dgkh,D130015C16,593(	PREDICTED: similar to diacylglycerol kinase, e	6.62E-20	1.51	2.40E-01	1.18	2.71E-02	1.39
A_51_P159201	Junb	Jun-B oncogene	3.24E-10	-1.41	9.22E-07	-1.78	3.54E-02	-1.32
A_52_P583058	Hira,N28177,Tuple1,D1	Histone cell cycle regulation defective homol	2.00E-05	1.26	1.17E-01	1.96	5.79E-01	1.06
A_52_P76988	Sp2,mKIAA0048,49304	Sp2 transcription factor	4.10E-04	1.53	6.17E-01	1.26	6.46E-01	-1.10
A_52_P40504	2900052L18Rik	PREDICTED: hypothetical protein XP_488619	8.50E-07	1.53	2.22E-09	1.42	4.44E-02	1.16
A_52_P503208	XM_358717	Mus musculus hypothetical gene supported l	4.59E-01	1.06	8.97E-07	1.51	9.78E-01	-1.00
A_52_P443201	Gababrbp,Marlin-1,583	gamma-aminobutyric acid (GABA-B) receptor	5.12E-15	2.02	1.57E-02	1.49	6.86E-01	1.06
A_51_P138002	Zfp26,Zfp70,mkr-3,KRA	MRNA of mkr3 gene encoding zinc finger pro	5.50E-02	1.10	1.20E-04	1.66	3.73E-01	1.06
A_52_P103319	NAP038609-1	Unknown	0.00E+00	1.72	3.20E-04	1.44	9.85E-01	-1.00
A_52_P1131525	Pcbp3,AlphaCP-3	Poly(rC) binding protein 3 (Pcbp3), mRNA	3.98E-06	1.57	4.02E-01	1.15	6.16E-01	1.12
A_51_P300395	1700007K09Rik	RIKEN cDNA 1700007K09 gene (1700007K09	2.49E-01	1.17	5.02E-08	2.42	7.30E-01	-1.03
A_52_P4095	TC1087876	BC026525 Auh protein {Mus musculus}, part	5.39E-01	-1.08	4.80E-04	-1.71	9.25E-01	1.02
A_52_P311853	Ddit4l,REDD2,Smhs1,R1	RIKEN cDNA 1700037B15 gene	4.17E-03	-1.14	5.66E-12	-1.53	4.66E-01	1.05
A_51_P407146	Trip11,AI450776,26105	Thyroid hormone receptor interactor 11, mR	9.58E-02	1.14	5.85E-07	1.60	1.00E-01	1.15
A_51_P219266	Tmprss6,1300008A22R	RIKEN cDNA 1300008A22 gene	1.12E-02	1.35	7.29E-34	2.10	3.32E-02	1.25
A_52_P6092	D330050I23Rik,BF6428	Mus musculus RIKEN cDNA D330050I23 gene	7.50E-12	-1.47	3.00E-04	-1.53	9.45E-01	1.01
A_52_P420792	Txnip,THIF,VDUP1,mVD	Thioredoxin interacting protein, mRNA (cDN/	5.33E-13	1.89	3.51E-09	2.35	1.86E-01	-1.15
A_52_P466171	Ddx26,HDB,DICE1,Notc	DEAD/H (Asp-Glu-Ala-Asp/His) box polypepti	3.00E-04	-1.74	3.72E-01	-1.49	7.35E-01	1.08
A_51_P198114	Lincr,2010300P06Rik	lung-inducible neuralized-related C3HC4 RIN	3.10E-04	1.66	3.60E-01	1.33	7.90E-01	1.07
A_51_P454696	Mosc1,1300013F15Rik	MOCO sulphurase C-terminal domain contair	6.27E-01	-1.05	5.00E-05	-1.56	1.83E-01	1.56
A_52_P996186	Unc5c,rcm,Unc5h3,AI0	Unc-5 homolog C (C. elegans) (Unc5c), mRNA	1.09E-15	1.83	1.84E-02	1.67	4.42E-01	1.10
A_52_P779532	Usp24,C79851,AI41405	Ubiquitin specific peptidase 24, mRNA (cDNA	6.12E-09	1.64	1.00E+00	1.00	5.71E-01	1.14
A_52_P675052	Golgb1,AU042952,KIAA	Mus musculus golgi autoantigen, golgin subf	4.37E-02	-1.24	2.32E-03	-1.82	3.30E-04	1.20
A_51_P104687	Gh	growth hormone	7.19E-01	1.21	1.32E-01	3.06	6.32E-19	-3.67
A_52_P891406	Capn6	Calpain 6 (Capn6), mRNA	1.24E-06	1.68	4.64E-01	1.48	7.78E-01	-1.08
A_51_P204371	AK051810	12 days embryo eyeball cDNA, RIKEN full-len	7.19E-13	1.56	7.52E-01	1.15	2.09E-01	1.30
A_51_P266987	Odf2,AI848335,MMTES	outer dense fiber of sperm tails 2	7.29E-01	1.04	9.20E-08	1.51	4.55E-01	-1.05
A_51_P246653	Clec7a,BGR,beta-GR,Cl	C-type (calcium dependent, carbohydrate re	9.93E-01	-1.00	1.70E-04	-2.21	1.12E-02	-1.23
A_52_P586679	Six1,BB138287	sine oculis-related homeobox 1 homolog (Dr	1.94E-16	-1.71	7.86E-02	-1.29	4.18E-01	1.13
A_52_P528996	Mxd4,Mad4,2810410A(	Max dimerization protein 4, mRNA (cDNA clc	6.49E-10	1.60	1.00E-05	1.78	2.12E-01	-1.16
A_51_P422300	Ms4a6b,1810027D10Ri	membrane-spanning 4-domains, subfamily A	9.37E-09	1.36	2.58E-17	1.93	2.28E-01	-1.10
A_52_P640337	Gng5,G(y)5	Mus musculus guanine nucleotide binding pr	1.78E-12	-2.50	6.80E-01	1.16	8.85E-02	-1.26
A_51_P156394	1700010C24Rik	RIKEN cDNA 1700010C24 gene	7.73E-01	-1.03	2.49E-10	-1.75	3.26E-01	1.09
A_52_P188401	D930036F22Rik,mKIAA	RIKEN cDNA D930036F22 gene, mRNA (cDN/	9.00E-05	-1.44	6.60E-04	-1.62	4.81E-01	-1.15
A_52_P285100	Gcn1l1,GCN1L,AL02276	GCN1 general control of amino-acid synthesi	1.27E-25	1.55	9.01E-01	1.06	5.59E-01	1.05
A_52_P499082	Txndc1,AI465319,2810	thioredoxin domain containing 1	8.34E-01	-1.01	4.70E-04	1.70	2.84E-01	-1.08
A_51_P441103	Oxsr1,Osr1,AI462649,A	Oxidative-stress responsive 1, mRNA (cDNA c	4.20E-04	4.12	1.00E+00	1.00	1.00E+00	1.00
A_52_P504786	Mgea5,Hy5,AA408215,i	meningioma expressed antigen 5 (hyaluronic	5.00E-05	1.28	2.04E-03	1.66	9.17E-01	1.00
A_52_P293927	NAP052311-1	Unknown	6.11E-12	1.81	7.00E-05	2.52	3.40E-01	-1.11
A_52_P291	Adcy7,AA407758	Adenylate cyclase 7 (Adcy7), mRNA	1.60E-04	1.44	1.00E-05	1.68	3.35E-01	1.07
A_51_P229105	Cetn3,MmCEN3	centrin 3	6.77E-01	1.04	6.02E-16	1.57	4.12E-01	-1.07
A_52_P762853	2900027M19Rik	Transmembrane protein 28 (Tmem28), mRN.	1.39E-38	1.66	6.90E-01	1.28	3.74E-02	1.24
A_52_P412506	Mup5	major urinary protein 5	2.04E-01	-1.13	0.00E+00	-21.51	4.98E-01	1.61
A_51_P209782	Cyp2c44,AW107714,BC	Mus musculus cDNA sequence BC034834 (B(	2.52E-01	-1.07	2.93E-07	-1.72	1.52E-01	1.53
A_52_P415440	TC1029210	JC4159 ribosomal protein S12 - pig, partial (3	9.00E-04	1.28	3.60E-04	1.77	5.86E-01	-1.03
A_52_P298824	Slc7a11,sut,xCT,99300(	Solute carrier family 7 (cationic amino acid tr	1.68E-09	-1.64	2.76E-01	-1.20	6.68E-01	1.05
A_52_P320822	5730405G21Rik	RIKEN cDNA 5730405G21 gene	8.41E-45	1.57	3.94E-01	1.37	5.25E-01	1.08
A_51_P405476	Fcer1g,CD23,Fce1g,Ly-5	Fc receptor, IgE, high affinity I, gamma polyp	3.20E-01	1.07	8.78E-10	-1.51	5.77E-01	-1.08
A_51_P335112	Upf2	UPF2 regulator of nonsense transcripts homc	6.81E-01	-1.10	2.00E-05	-1.84	9.49E-01	-1.03
A_51_P176583	1700109H08Rik,MGC11	RIKEN cDNA 1700109H08 gene, mRNA (cDN/	4.79E-02	1.17	2.76E-11	1.73	8.62E-01	-1.02
A_51_P322933	Rpl27,MGC103134	Sperm associated antigen 9 (Spag9), transcri	4.89E-09	-1.74	7.50E-01	-1.06	1.63E-01	-1.12
A_51_P390038	Cpa6,9030616D13Rik	hypothetical protein 9030616D13	5.15E-01	1.16	4.00E-05	1.53	6.50E-06	-1.68
A_51_P250349	Slc1a1,EAAC1,EAAC2,E/	Solute carrier family 1 (neuronal/epithelial hi	9.62E-01	-1.00	3.00E-05	1.55	4.38E-01	-1.17
[illegible]	[illegible]	[illegible]	[illegible]	[illegible]	[illegible]	[illegible]	[illegible]	[illegible]

A_51_P166886	Saa2,Saa1,Saa-2,AW111	serum amyloid A 2	4.02E-01	-1.08	0.00E+00	-4.49	8.90E-02	2.05
A_51_P175580	Trp53inp1,SIP,Teap,SIP	transformation related protein 53 inducible r	1.76E-03	1.14	4.20E-45	2.48	1.96E-01	-1.16
A_52_P381665	Aff1,Af4,Rob,Mllt2h,AW	Mus musculus adult male thymus cDNA, RIKE	9.11E-01	-1.01	3.21E-09	1.84	7.73E-01	-1.02
A_52_P644320	Sema3e,Semah,AA4088	Sema domain, immunoglobulin domain (Ig), s	7.72E-09	2.75	1.00E+00	1.00	1.00E+00	1.00
A_51_P151722	BC053994	PREDICTED: Mus musculus similar to GD:RP1	7.62E-12	1.97	1.64E-01	2.01	2.30E-01	1.41
A_52_P972159	Fgf1,Fam,Fgfa,Dffrx,Fgf	Fibroblast growth factor 1, mRNA (cDNA clon	1.01E-13	1.40	3.54E-07	-1.53	3.01E-01	1.24
A_52_P448060	2210010C04Rik,AV0722	RIKEN cDNA 2210010C04 gene	4.99E-01	-1.04	5.15E-08	-1.98	1.26E-01	1.76
A_51_P264548	4933427E11Rik	RIKEN cDNA 4933427E11 gene	7.58E-01	1.04	1.79E-19	1.75	1.97E-01	1.13
A_52_P314608	Txndc10,AV259382,mK	Thioredoxin domain containing 10 (Txndc10)	2.70E-07	-1.91	2.55E-02	-2.76	6.70E-01	-1.16
A_51_P260789	Rbm11,A330018F01	RNA binding motif protein 11 (Rbm11), mRN	5.21E-01	1.07	4.21E-24	1.91	2.91E-01	-1.07
A_51_P316553	Kdr,Flk1,Ly73,Flk-1,Krd-	kinase insert domain protein receptor	8.31E-01	-1.01	7.25E-25	-2.39	5.18E-01	1.04
A_51_P197718	Nkx2-2,Nkx2.2,tinman,I	NK2 transcription factor related, locus 2 (Drc	9.81E-03	-1.18	8.50E-08	-1.61	3.85E-01	-1.03
A_52_P322514	Rfx3,MRFX3,C230093O	Regulatory factor X, 3 (influences HLA class II	0.00E+00	1.97	4.31E-03	1.37	3.74E-01	-1.07
A_52_P957260	Wdr79,BC021790,MGC	WD repeat domain 79 (Wdr79), mRNA	8.22E-10	1.92	1.86E-03	1.59	1.04E-01	1.11
A_51_P189082	Akr1c6,Akr1c1,Hsd17b5	aldo-keto reductase family 1, member C6	2.06E-01	-1.28	1.78E-13	-15.31	7.13E-02	4.63
A_52_P1123908	R3hdm1,R3hdm	R3H domain (binds single-stranded nucleic ac	5.40E-15	-1.66	6.21E-01	-1.10	5.42E-01	1.12
A_52_P194250	Ets1,Tpl1,Ets-1,MGC18!	E26 avian leukemia oncogene 1, 5' domain, r	1.63E-01	-1.09	6.96E-11	-1.59	2.89E-01	1.06
A_52_P67574	1700129I04Rik,D03006	RIKEN cDNA 1700129I04 gene (1700129I04R	7.66E-06	1.33	3.35E-21	2.62	1.39E-01	-1.24
A_52_P121703	LOC432625	hypothetical gene supported by AK076489	3.56E-01	1.09	3.23E-14	1.57	4.61E-01	-1.06
A_52_P34979	Epc1,2400007E14Rik,57	Enhancer of polycomb homolog 1 (Drosophil	1.41E-25	1.70	1.90E-06	2.53	1.67E-01	1.07
A_51_P462790	1700106N22Rik	PREDICTED: hypothetical protein LOC73582		1.45E-12	2.08	2.51E-02	1.80	9.50E-02	1.98
A_51_P380991	Krt1-4,Ha2,4733401E01	keratin complex 1, acidic, gene 4	2.51E-01	1.24	3.81E-07	2.67	7.27E-01	-1.17
A_52_P516817	NAP108366-1	Unknown	1.10E-04	1.82	1.00E+00	1.00	6.45E-01	1.20
A_51_P342773	TC950516	VIPS_MOUSE Vasoactive intestinal polypepti	2.79E-01	-1.05	6.62E-15	1.64	9.30E-02	-1.14
A_51_P291417	Thbd,TM,CD141,AI3855	thrombomodulin	1.86E-01	-1.15	4.58E-14	-1.65	5.91E-01	1.06
A_52_P266466	Snap91,91kDa,AP180,F	Synaptosomal-associated protein 91, mRNA	4.70E-08	1.79	1.13E-03	2.46	2.80E-01	1.23
A_52_P653456	TC1029926	BC035974 DEAD/H (Asp-Glu-Ala-Asp/His) box	2.52E-12	1.43	6.00E-05	2.16	4.43E-01	-1.04
A_51_P488649	9530067L11Rik	Mus musculus adult male urinary bladder cD	3.12E-02	1.69	1.20E-04	3.03	6.02E-01	1.29
A_52_P122654	Glp2r,GLP-2,9530092J0	glucagon-like peptide 2 receptor	3.23E-01	-1.08	1.43E-10	1.62	2.28E-03	1.33
A_51_P217218	Il6,Il-6	interleukin 6	2.07E-01	1.31	5.17E-19	2.22	3.80E-02	1.26
A_52_P197965	Erdr1,edr,MGC5764	erythroid differentiation regulator 1	5.19E-01	-1.13	1.19E-35	-1.63	2.44E-03	1.23
A_52_P196442	Prpf39,Srcs1,MGC3707	PRP39 pre-mRNA processing factor 39 homo	1.00E-05	1.59	8.36E-01	1.08	6.07E-01	1.06
A_51_P102438	Ugt2b37,0610033E06Ri	RIKEN cDNA 0610033E06 gene	9.80E-01	-1.01	1.09E-08	-6.25	3.21E-01	1.81
A_51_P144222	Otop2,4732464P15	otopetrin 2	4.96E-01	1.07	3.50E-04	1.63	2.67E-02	-1.29
A_52_P851352	Igfbp3,IGFBP-3,IGgfbp3	Insulin-like growth factor binding protein 3 (I	4.85E-06	1.68	7.28E-01	1.14	7.71E-01	1.12
A_51_P313779	Zbtb40,Gm571,BC0591	hypothetical protein C230087D24	4.12E-01	-1.05	0.00E+00	-1.76	1.13E-02	1.11
A_52_P50090	Sfrs5,MGC96781	splicing factor, arginine/serine-rich 5 (SRp40,	3.47E-03	1.25	7.87E-06	1.80	4.08E-01	-1.05
A_51_P394802	4632417K18Rik,AW413	expressed sequence AW413625	1.93E-01	-1.09	3.61E-09	-1.95	9.30E-01	-1.01
A_52_P480309	4930470G03Rik	RIKEN cDNA 4921509E05 gene (4921509E05	2.79E-06	1.36	1.19E-02	1.51	2.46E-01	1.11
A_51_P416647	Klk13,Egfbp2,mGk-13,E	kallikrein 13	4.63E-01	1.13	9.60E-13	-3.63	7.43E-01	-1.09
A_52_P207314	Htra4,B430206E18Rik	Mus musculus hypothetical protein B430206	1.41E-02	1.18	3.67E-09	2.20	1.15E-03	1.19
A_52_P531610	Agxt2l1,AI195447,1300	Alanine-glyoxylate aminotransferase 2-like 1	6.57E-02	1.11	1.16E-17	2.04	1.99E-01	-1.15
A_51_P351166	Myod1,MYF3,MyoD,My	myogenic differentiation 1	6.99E-01	-1.17	1.34E-21	-1.93	2.05E-11	2.86
A_52_P671495	Sorcs1,Sorcs	Mus musculus adult male pituitary gland cDN	4.91E-01	1.07	2.01E-19	1.64	2.54E-01	-1.13
A_51_P283456	Cyp2e1,Cyp2e	cytochrome P450, family 2, subfamily e, poly	2.27E-01	-1.92	0.00E+00	-17.41	2.23E-02	5.89
A_52_P582619	6330415G19Rik	PREDICTED: similar to Zinc finger protein 213	5.07E-01	1.04	4.98E-08	1.55	2.83E-01	-1.09
A_51_P428483	Fgb,2510049G14Rik	fibrinogen, B beta polypeptide	3.08E-01	1.29	0.00E+00	-10.98	1.42E-09	6.43
A_52_P661029	A730055C05Rik,RP23-6	RIKEN cDNA A730055C05 gene	8.49E-01	-1.02	1.00E-04	1.90	1.58E-02	-1.20
A_51_P321341	Sult1a1,PST,Stp,Stp1,AI	sulfotransferase family 1A, phenol-preferring	1.39E-01	1.09	1.02E-06	2.17	7.25E-08	1.41
A_51_P204329	Slc16a14,1110004H10R	Solute carrier family 16 (monocarboxylic acic	2.49E-10	1.66	1.12E-01	1.69	8.57E-01	1.03
A_52_P311941	Pcdh15,av,Ush1f,nmf19	Protocadherin 15 (Pcdh15), mRNA	9.60E-11	1.74	7.97E-01	-1.12	2.41E-01	1.21
A_51_P164817	Adamts2,PCINP,hPCPNI	10 days neonate skin cDNA, RIKEN full-length	3.13E-06	1.40	1.87E-08	1.96	1.00E+00	1.00
A_51_P270426	Egr4,NGFIC,NGF1-C,NG	early growth response 4	1.08E-02	-1.32	1.00E-05	-1.75	1.93E-01	-1.26
A_52_P36191	TC1010010	AF302077 neprilysin-like peptidase gamma {I	3.10E-13	-1.52	4.94E-01	-1.10	1.99E-01	1.29
A_52_P237927	Olfr937,MOR171-24	olfactory receptor MOR171-24	1.91E-01	2.17	2.50E-04	-2.74	1.00E+00	1.00
A_51_P517645	2310014F07Rik	Adult male tongue cDNA, RIKEN full-length e	2.45E-03	1.79	1.00E-04	2.32	4.79E-02	1.41
A_51_P251737	LOC574405	hypothetical protein LOC574405	3.29E-01	1.09	3.21E-09	1.56	3.49E-01	1.10
A_52_P293391	Tcam1,AI429105,49305	testicular cell adhesion molecule 1	2.07E-03	-1.15	1.38E-11	1.98	4.19E-01	1.08
A_51_P394512	Phf3,AI315311,mKIAA0	PHD finger protein 3, mRNA (cDNA clone IMA	7.32E-03	1.19	3.03E-09	1.68	4.23E-01	1.09
A_52_P598885	Slc16a4,AW146050,MG	solute carrier family 16 (monocarboxylic acid	9.01E-02	-1.12	3.11E-06	-1.77	3.12E-02	-1.14
A_51_P519992	Lgr7,Gm1018	Mus musculus 2 days pregnant adult female	1.00E-05	1.54	6.93E-01	1.05	6.00E-02	1.25
A_51_P235798	Zfp36l1,Brf1,ERF1,cMG	Zinc finger protein 36, C3H type-like 1, mRNA	6.70E-02	-1.15	8.48E-07	-1.55	3.10E-01	-1.12
A_51_P492830	Cenph,ENP,CENP-H,AU	centromere autoantigen H	5.64E-01	-1.07	4.93E-13	1.68	5.89E-03	-1.21
A_51_P495825	Apof,AI255964	apolipoprotein F	8.61E-01	-1.02	6.50E-16	-2.50	2.25E-01	-1.22
A_52_P1027808	B430316J06Rik	Putative membrane-associated guanylate kir	2.37E-10	1.43	6.98E-03	-1.79	2.44E-02	1.47
A_51_P441933	4931423N10Rik	PREDICTED: RIKEN cDNA 4931423N10 [Mus	9.62E-01	-1.01	5.89E-10	2.12	4.50E-03	-1.23
A_52_P368772	NAP028748-1	Unknown	9.15E-02	1.07	1.50E-04	1.60	7.61E-01	-1.01
A_52_P947954	Myst3,MOZ,Zfp220,150	PREDICTED: MYST histone acetyltransferase	2.57E-03	1.69	6.96E-06	1.92	5.52E-01	1.30
A_52_P86165	9430040K09Rik	Mortality factor 4 like 1, mRNA (cDNA clone	4.13E-09	1.40	5.72E-03	1.74	2.91E-02	1.34
A_52_P198824	4921505C17Rik,AVO3,A	RIKEN cDNA 4921505C17 gene, mRNA (cDNA	5.34E-08	1.35	3.53E-02	1.52	7.77E-01	1.03
A_52_P323621	XM_193319	Mus musculus similar to Methionine adenosy	3.95E-01	1.05	2.49E-07	1.70	3.35E-01	-1.09
A_52_P571691	6030443O07Rik,AU014	PREDICTED: hypothetical protein LOC226151	1.19E-17	1.50	4.46E-07	1.69	1.45E-01	1.09
A_52_P683336	AY074887,Mcpr1	cleft palate-related protein 1	2.80E-01	-1.25	6.38E-06	-1.72	4.51E-01	1.31
A_52_P160181	Impg1,SPACR,IMP150,A	interphotoreceptor matrix proteoglycan 1	7.31E-01	-1.14	4.10E-04	-3.71	1.00E+00	1.00
A_52_P248013	4833446K15Rik,AI6622	10 days neonate cortex cDNA, RIKEN full-len	1.35E-02	-1.10	5.00E-05	-1.54	1.97E-01	1.05
A_52_P415735	Zfp53,KRAZ1,zfas8,Zfp-	Zinc finger protein 118	5.05E-01	1.04	3.00E-05	1.55	1.19E-01	-1.10
A_52_P352246	Sgk,Sgk1	Mus musculus 7 days neonate cerebellum cD	2.12E-03	1.17	3.70E-04	1.89	9.49E-01	-1.01
[illegible]	[illegible]	[illegible]	[illegible]	[illegible]	[illegible]	[illegible]	[illegible]	[illegible]

A_52_P914882	4930406M16Rik	Adult male testis cDNA, RIKEN full-length enr	2.99E-02	-1.11	7.99E-13	-2.88	3.35E-01	1.12
A_51_P427530	Pgm1,Pgm2,Pgm-1,323	phosphoglucomutase 1	1.17E-01	1.12	2.88E-06	1.71	4.24E-01	-1.04
A_52_P1028636	AK086157	Mus musculus 15 days embryo head cDNA, R	1.70E-04	1.99	2.66E-12	4.89	3.97E-01	-1.68
A_52_P963766	AK054126	2 days pregnant adult female oviduct cDNA,	6.65E-15	1.88	1.00E+00	1.00	1.79E-02	1.85
A_51_P250590	Sbf2,Mtmr13,mMTMH1	Myotubularin related protein 13, mRNA (cDN	7.13E-12	1.82	1.50E-01	1.62	5.06E-01	1.08
A_51_P427624	Pgc,Upg1,Upg-1,MGC1	progastricsin (pepsinogen C)	3.90E-04	1.18	4.21E-03	1.67	2.26E-01	-1.35
A_52_P384100	Bdnf	brain derived neurotrophic factor	2.36E-01	-1.17	2.09E-06	-1.61	1.26E-01	-1.09
A_52_P577484	Sox9,AV220920,KIAA42	SRY-box containing gene 9	1.25E-01	-1.12	1.16E-06	-1.63	3.40E-01	1.07
A_52_P261625	NAP042239-1	Unknown	1.19E-11	1.31	1.03E-01	1.64	8.79E-01	1.01
A_52_P283119	BB530856	BB530856 RIKEN full-length enriched, 0 day r	1.24E-01	1.14	2.04E-19	1.70	5.63E-01	-1.05
A_52_P194531	TC1044100	Unknown	2.00E-05	1.26	1.06E-02	1.53	8.47E-01	1.02
A_52_P448994	Col4a1,Col4a-1	Procollagen, type IV, alpha 1, mRNA (cDNA c	5.80E-07	1.58	1.00E-05	1.55	5.59E-01	-1.07
A_51_P448127	2410004A20Rik,ECAT1,	RIKEN cDNA 2410004A20 gene	7.60E-06	-1.38	1.14E-02	-1.79	9.94E-01	1.00
A_52_P392638	Bin1,ALP-1,Amphl,SH3P	Bridging integrator 1 (Bin1), mRNA	8.14E-06	1.54	7.32E-01	1.15	7.77E-01	-1.08
A_51_P151214	C8b,AI595927,4930439	complement component 8, beta subunit	2.90E-01	-1.31	9.52E-02	-1.60	5.57E-06	4.25
A_51_P222324	Adrbk2,GRK3,Bark-2,Ac	RIKEN cDNA 4833444A01 gene	7.12E-01	1.02	4.00E-05	-2.37	6.90E-01	1.04
A_52_P284873	Slc7a6,LAT3,LAT-2,AI64	Mus musculus 16 days neonate thymus cDNA	1.06E-02	-1.15	3.70E-04	1.60	1.18E-01	1.12
A_51_P428555	Adh1,Adh-1,ADH-AA,Ac	alcohol dehydrogenase 1 (class I)	7.47E-01	1.10	0.00E+00	-2.93	3.89E-01	1.37
A_52_P380379	Ucp3,UCP-3,Slc25a9,AI	uncoupling protein 3, mitochondrial	6.60E-04	1.14	7.49E-16	1.76	3.34E-01	-1.05
A_51_P470793	Dcst1,A330106H01Rik	PREDICTED: hypothetical protein LOC77772		1.89E-01	-1.11	2.20E-06	-1.56	9.87E-01	-1.00
A_52_P422494	Cd300lf,CLM1,CLIM1,Cl	Polymeric immunoglobulin receptor 3 precur	1.39E-02	1.23	1.82E-06	2.71	6.75E-01	1.10
A_52_P123693	E130304I02Rik	0 day neonate eyeball cDNA, RIKEN full-lengt	3.64E-01	-1.26	2.14E-16	-1.98	4.31E-01	1.35
A_51_P487175	Acsm3,Sa,Sah	Mus musculus SA rat hypertension-associate	1.80E-01	-1.16	7.00E-05	2.40	2.92E-02	2.03
A_51_P275027	Dgkb,DGK,90kda,DAGK	Diacylglycerol kinase, beta, mRNA (cDNA clo	1.00E-05	1.66	9.36E-02	1.31	5.81E-01	1.34
A_52_P663492	Tmem16f,AA407480,A\	Transmembrane protein 16F, mRNA (cDNA c	6.59E-01	1.06	9.96E-06	2.44	3.41E-01	-1.06
A_51_P125648	1300015B04Rik	RIKEN cDNA 1300015B04 gene, mRNA (cDNA	3.30E-01	1.11	4.00E-09	1.83	1.08E-01	1.18
A_52_P338737	Phf14,AA623952,AV297	PHD finger protein 14 (Phf14), mRNA	5.71E-01	-1.08	4.00E-05	1.60	7.12E-01	1.09
A_51_P317357	Ptprk,AI853699	Protein tyrosine phosphatase, receptor type,	3.50E-04	1.36	1.40E-07	1.63	8.68E-02	1.13
A_52_P1075615	D18Ertd232e	Ring finger protein 125, mRNA (cDNA clone I	1.97E-07	1.80	7.76E-03	2.13	9.41E-01	-1.01
A_51_P286665	Rbl1,PRB1,p107,AW547	retinoblastoma-like 1 (p107)	9.54E-01	1.00	1.10E-04	-1.57	5.49E-01	1.04
A_52_P169212	1700072H12Rik	RAN binding protein 17 (Ranbp17), mRNA	3.82E-25	1.87	1.05E-10	2.30	2.22E-02	-1.28
A_51_P401828	Mug2	murinoglobulin 2	1.19E-01	1.26	0.00E+00	-23.98	7.00E-05	7.66
A_52_P787134	AK047305	Mus musculus 10 days neonate cerebellum c	1.69E-01	-1.44	2.48E-07	-3.47	9.51E-01	1.04
A_51_P144170	4933400E14Rik,Gm497	PREDICTED: hypothetical protein LOC71007		4.46E-01	-1.59	4.20E-04	-2.65	7.26E-01	1.17
A_51_P212211	9430031K09Rik	Echinoderm microtubule associated protein l	2.79E-02	-1.18	5.51E-07	-1.73	2.50E-01	1.11
A_51_P257151	Adipor2,ADCR2,Paqr2,A	Adiponectin receptor 2, mRNA (cDNA clone N	1.74E-09	1.41	1.46E-03	3.45	5.36E-01	1.04
A_52_P48681	Cldn1,AI596271	claudin 1	3.68E-01	1.06	6.23E-11	2.51	6.60E-04	-1.26
A_51_P429903	Ndph,ND,NDP	Norrie disease homolog	1.05E-03	-1.20	1.46E-09	-1.56	3.22E-01	-1.04
A_51_P131561	1600029D21Rik,PLET1,	RIKEN cDNA 1600029D21 gene	3.97E-01	1.12	2.69E-06	1.87	7.07E-01	-1.06
A_52_P721851	4921522E08Rik	Mus musculus adult male testis cDNA, RIKEN	6.75E-02	-1.22	2.60E-04	-1.97	2.50E-04	1.20
A_52_P979517	AK038959	12 days embryo eyeball cDNA, RIKEN full-len	2.00E-05	1.33	3.00E-05	-1.54	1.16E-01	1.21
A_51_P267278	Slc15a2,Pept2,C78862,S	solute carrier family 15 (H+/peptide transpor	1.21E-02	-1.41	3.81E-08	-1.52	8.28E-17	-1.42
A_51_P408689	BB618745	8 days embryo whole body cDNA, RIKEN full-	7.17E-01	-1.03	1.04E-13	1.66	1.48E-01	-1.12
A_52_P186033	Spn,Cd43,Ly48,Galgp,Ly	Sialophorin (Spn), mRNA	3.00E-05	1.72	1.88E-01	1.33	2.03E-01	-1.13
A_52_P1187714	Foxp2,CAG-16,D0Kist7,	Forkhead/winged-helix transcription factor 2	6.69E-01	-1.06	4.22E-10	-2.34	3.18E-01	1.31
A_52_P365781	BC027127,MGC38877,S	CDNA sequence BC027127 (BC027127), mRN	1.63E-01	1.24	1.03E-34	2.05	1.39E-01	-1.21
A_51_P403610	Olfr1339,MOR258-2	Mus musculus olfactory receptor MOR258-2	4.28E-01	-1.09	1.19E-08	-1.77	5.13E-01	1.10
A_52_P670188	Sdro,Rdhs,SDR-O,1810	orphan short chain dehydrogenase/reductas	3.45E-02	-1.27	4.64E-06	-1.74	1.93E-01	1.17
A_52_P540024	Lmo7,Gm914,C78582,F	PREDICTED: LIM domain only 7 [Mus muscul	3.53E-02	-1.30	1.16E-01	-1.69	2.03E-09	1.28
A_51_P515002	C130057N11Rik,AI6666	HnRNP-associated with lethal yellow (Raly), r	9.50E-01	1.00	6.68E-11	1.54	1.71E-01	-1.10
A_52_P487156	1700029I01Rik	Mus musculus RIKEN cDNA 1700029I01 gene	3.58E-01	1.05	1.20E-04	1.68	3.68E-01	-1.08
A_51_P424854	Ahsg,fetuin-A	alpha-2-HS-glycoprotein	3.24E-01	-1.27	0.00E+00	-16.18	1.67E-08	11.23
A_51_P314774	5430405G24Rik,AU018	RIKEN cDNA 5430405G24 gene, mRNA (cDNA	8.38E-03	1.25	1.49E-17	1.77	8.21E-01	-1.03
A_52_P347031	B430203M17Rik	RIKEN cDNA B430203M17 gene	1.04E-09	1.79	4.61E-01	1.46	9.67E-01	-1.01
A_52_P1173329	Zfp395,Gm910,BC0537	CDNA sequence BC027382, mRNA (cDNA clo	1.25E-07	-1.48	7.06E-08	-1.66	4.00E-05	1.17
A_52_P1140055	2610028H07Rik	RIKEN cDNA 2610028H07 gene, mRNA (cDNA	5.63E-22	1.53	1.70E-04	1.30	1.20E-04	1.61
A_52_P327146	4732447D17Rik	Mus musculus 13 days embryo male testis cD	6.39E-18	1.71	2.63E-02	1.25	2.00E-05	1.38
A_52_P349380	Chn2,Bch,ARHGAP3,17	Beta chimaerin (bch gene)	2.96E-11	1.63	2.13E-01	1.21	1.00E-05	1.37
A_51_P383822	Evi2b,Evi2,Evi-2,MGC28	ecotropic viral integration site 2B	3.59E-01	-1.10	1.00E-04	-1.76	3.28E-02	-1.20
A_51_P267836	Plg,Pg,AI649309	plasminogen	5.90E-01	1.16	1.38E-08	-4.18	7.52E-02	3.87
A_51_P505662	4933437F05Rik,MGC11	PREDICTED: hypothetical protein LOC71275		4.76E-02	-1.30	5.85E-08	-1.72	7.12E-01	1.05
A_51_P214087	Cebpa,Cebp,CBF-A	CCAAT/enhancer binding protein (C/EBP), alp	5.64E-01	1.04	7.00E-05	-2.01	3.02E-01	-1.07
A_51_P320146	AK046159	Mus musculus adult male corpora quadrigem	4.22E-10	1.66	1.00E+00	1.00	9.91E-01	-1.00
A_52_P483339	Aars,C76919,AI316495,	Mus musculus adult male bone cDNA, RIKEN	2.43E-06	1.32	1.55E-01	1.65	6.02E-01	1.08
A_51_P128973	Apoa2,Alp-2,Hdl-1,Apo	apolipoprotein A-II	3.70E-01	-1.08	0.00E+00	-3.86	7.76E-01	-1.03
A_52_P308800	Psme4,TEMO,AA40939	Proteasome (prosome, macropain) activator	5.12E-14	1.78	1.08E-02	3.26	9.43E-01	-1.01
A_52_P328440	LOC384021	similar to alpha-2u-globulin V precursor - mo	5.73E-01	-1.10	1.21E-14	-32.85	3.36E-03	6.79
A_52_P875290	AK030286	11 days pregnant adult female ovary and ute	6.20E-23	-1.82	4.36E-01	-1.35	8.46E-03	1.23
A_52_P550173	Slamf1,Slam,CD150,IPC	Type I membrane receptor SLAM1 (Slam)	7.57E-01	-1.06	2.80E-04	-2.62	9.63E-01	1.01
A_52_P731758	AK078294	Mus musculus adult male olfactory brain cDN	2.81E-07	1.27	8.36E-19	1.84	9.00E-01	1.01
A_52_P218792	3830406C13Rik,AV1679	RIKEN cDNA 3830406C13 gene	6.44E-01	-1.02	2.24E-10	1.57	5.01E-03	-1.12
A_52_P569327	Usp53,Sp6,Phxr3,AA93	Ubiquitin specific peptidase 53, mRNA (cDNA	1.85E-02	1.28	2.18E-06	1.78	6.91E-01	-1.03
A_52_P108223	AK084877	0 day neonate eyeball cDNA, RIKEN full-lengt	4.79E-06	1.38	1.77E-01	1.52	4.92E-01	1.10
A_52_P384209	Syne1,8B,CPG2,Myne1,	Synaptic nuclear envelope 1 (Syne1), transcri	2.53E-06	1.63	3.09E-01	1.47	1.70E-01	-1.63
A_51_P262701	Cpb2,CPR,Cpu,TAFI,AI2	carboxypeptidase B2 (plasma)	6.43E-01	-1.04	1.16E-24	-2.81	1.90E-01	1.62
A_52_P502083	E130014J05Rik	RIKEN cDNA E130014J05 gene	2.00E-05	1.46	2.70E-04	2.84	4.64E-01	1.05
A_52_P988461	Kif16b,C80253,C80902,	MKIAA1590 protein	1.62E-15	1.56	4.08E-01	-1.07	1.60E-01	1.18
[illegible]	[illegible]	[illegible]	[illegible]	[illegible]	[illegible]	[illegible]	[illegible]	[illegible]

A_52_P1108539	AK086969	Mus musculus 0 day neonate lung cDNA, RIK	3.60E-04	1.44	2.39E-02	2.05	5.10E-01	1.13
A_52_P916030	Pam	Peptidylglycine alpha-amidating monooxyge	2.93E-16	1.67	7.88E-01	1.05	4.36E-01	1.14
A_52_P265965	Trim36,haprin,D18Wsu	tripartite motif-containing 36	2.68E-02	-1.25	2.00E-05	-1.50	4.28E-02	-1.21
A_51_P129229	Ifi47,47kDa,Iigp4,Iipg4,I	interferon gamma inducible protein	1.64E-01	-1.14	1.10E-22	-2.00	7.50E-04	-1.18
A_51_P486121	Laf4	Mus musculus lymphoid nuclear protein rela	1.30E-04	1.66	8.33E-02	1.55	7.12E-01	-1.13
A_51_P287069	Serpinh1,J6,Cbp1,Cbp2,	serine (or cysteine) proteinase inhibitor, clad	3.60E-01	1.12	2.60E-06	-1.59	2.06E-02	1.12
A_51_P157112	Serpina3c,1A1,Kalbp,Kl	serine (or cysteine) proteinase inhibitor, clad	3.97E-01	-1.10	6.95E-11	-5.92	2.72E-03	2.99
A_52_P163105	D930030O05Rik	RIKEN cDNA D930030O05 gene	6.03E-06	2.21	4.59E-01	-1.53	8.80E-01	1.05
A_52_P679807	LOC383964	similar to ribosomal protein L21	4.84E-06	-1.83	3.62E-02	-1.19	9.02E-01	-1.01
A_51_P193573	Clk1,STY	CDC-like kinase	3.72E-13	-1.58	2.87E-02	-1.19	1.40E-01	-1.11
A_51_P405956	1700010A01Rik	Mus musculus adult male testis cDNA, RIKEN	1.33E-02	1.12	8.47E-26	1.54	1.07E-01	-1.20
A_52_P541752	Ddit4l,REDD2,Smhs1,R1	RIKEN cDNA 1700037B15 gene	7.35E-02	-1.08	7.76E-20	-1.50	2.79E-01	1.11
A_52_P1100015	AK038989	Mus musculus adult male hypothalamus cDN	3.26E-06	1.74	1.00E+00	1.00	1.04E-01	3.10
A_52_P627085	3830405G04Rik,AI5060	RIKEN cDNA 3830405G04 gene	2.85E-01	-1.07	1.02E-03	1.56	1.50E-04	1.18
A_51_P341203	Cyp3a41	cytochrome P450, family 3, subfamily a, poly	7.51E-01	-1.03	0.00E+00	-18.43	2.41E-01	2.13
A_52_P933175	Birc4,Aipa,Api3,IAP3,M	Baculoviral IAP repeat-containing 4 (Birc4), m	8.17E-01	-1.01	2.00E-05	1.63	7.98E-02	-1.09
A_52_P164136	Arrdc3,AI450344,MGC1	expressed sequence AI450344	5.00E-05	1.28	6.92E-08	1.54	7.61E-02	-1.16
A_51_P241068	Dkk2	dickkopf homolog 2 (Xenopus laevis)	9.51E-01	-1.01	1.20E-06	-1.92	5.89E-01	1.06
A_51_P316935	8430408G22Rik,Fseg,M	RIKEN cDNA 8430408G22 gene	4.41E-07	1.54	4.60E-04	2.30	1.10E-04	-1.58
A_51_P188381	C330018J07Rik	RIKEN cDNA C330018J07 gene	2.00E-05	1.41	2.91E-07	2.46	7.04E-01	-1.04
A_52_P1171783	AK046770	10 days neonate medulla oblongata cDNA, R	4.39E-13	1.55	4.67E-01	1.06	3.46E-01	1.18
A_52_P72737	1700066D14Rik	Nucleophosmin 1, mRNA (cDNA clone MGC:1	4.74E-02	-1.31	9.43E-08	-1.70	2.53E-01	1.15
A_52_P602406	NAP039513-1	Unknown	8.26E-01	1.03	2.00E-05	1.53	2.05E-02	-1.12
A_52_P1164335	Grm7,Gpr1g,Gprc1g,m(	PREDICTED: Mus musculus glutamate recept	7.60E-06	1.80	2.36E-02	2.17	5.44E-01	-1.17
A_52_P440265	9230110K08Rik	Adult male epididymis cDNA, RIKEN full-leng	6.95E-06	1.36	1.49E-29	2.13	9.83E-01	1.00
A_51_P344810	AK034668	12 days embryo embryonic body between di	2.73E-01	1.26	4.60E-04	2.24	6.08E-01	1.19
A_51_P349116	AK084405	Mus musculus 12 days embryo eyeball cDNA	1.00E-05	1.46	1.28E-08	-1.92	1.18E-01	1.24
A_52_P475229	Ep400,p400,mDomino,/	E1A binding protein p400	9.29E-02	1.11	1.00E-05	-1.96	6.98E-02	1.15
A_51_P459108	Insl6,RIF1	insulin-like 6	3.00E-05	1.31	2.82E-43	2.73	9.06E-01	-1.01
A_52_P106091	CB523073	UI-M-GK0-cet-m-24-0-UI.r1 NIH_BMAP_GK0	1.65E-08	-1.26	9.69E-02	-1.82	4.96E-03	-1.17
A_51_P302856	Ugt2b37,0610033E06Ri	RIKEN cDNA 0610033E06 gene	4.41E-01	1.14	8.26E-06	-2.60	5.19E-02	2.76
A_52_P630867	Abcc4	Mus musculus ATP-binding cassette, sub-fam	1.98E-03	1.15	4.54E-19	1.63	9.06E-01	1.01
A_52_P35881	4732460I02Rik	10 days neonate skin cDNA, RIKEN full-lengt	1.46E-01	1.09	1.37E-23	1.56	9.60E-02	1.06
A_52_P6070	4931408A02Rik,MGC11	C21orf63 protein (C21orf63)	3.06E-01	1.07	5.82E-35	1.69	9.53E-01	-1.00
A_51_P375969	Ces3,TGH	carboxylesterase 3	9.61E-02	-1.18	1.00E-05	-2.30	1.14E-02	4.38
A_51_P134142	Cyp2c70	cytochrome P450, family 2, subfamily c, poly	6.18E-01	-1.02	2.61E-31	-2.35	2.20E-01	1.27
A_52_P663040	D730045B01Rik	PREDICTED: Mus musculus RIKEN cDNA D73(	7.19E-08	1.54	1.50E-03	1.41	7.01E-02	1.17
A_52_P979803	Itgb8,4832412O06Rik	PREDICTED: integrin beta 8 [Mus musculus],	4.49E-06	1.38	4.95E-02	2.30	7.67E-01	1.09
A_52_P1036271	Fut9,mFUT9,AI746471,/	Fucosyltransferase 9 (Fut9), mRNA	1.63E-01	-1.08	6.46E-09	1.55	9.93E-01	-1.00
A_52_P119553	Zhx1,KIAA4149,mKIAA4	Zinc fingers and homeoboxes protein 1 (Zhx1	5.48E-01	-1.05	1.59E-13	1.75	9.57E-02	-1.16
A_52_P647069	G431002C21Rik,64305	RIKEN cDNA G431002C21 gene (G431002C2	3.77E-07	1.39	2.04E-01	1.59	5.62E-01	1.10
A_51_P193100	Gabrb3,Cp1,Gabrb-3,A	Gamma-aminobutyric acid (GABA-A) recepto	1.93E-17	1.70	5.54E-01	1.34	5.19E-01	1.16
A_51_P457187	Tnfrsf13c,Bcmd,Baffr,B	tumor necrosis factor receptor superfamily,	4.35E-01	1.14	1.26E-15	2.66	1.91E-02	-1.31
A_51_P305896	Cftr,Abcc7,AW495489	cystic fibrosis transmembrane conductance	5.03E-02	-1.13	2.04E-08	1.51	5.15E-01	1.07
A_51_P367980	Olfr1048,MOR187-2	olfactory receptor MOR187-2	4.71E-07	2.23	1.05E-09	-1.52	4.53E-01	1.34
A_52_P317524	Klhl15,MGC47138,633C	RIKEN cDNA 6330500C13 gene	3.69E-03	1.32	3.48E-09	2.15	2.00E-05	-1.20
A_52_P596375	Cckbr,CCK2R	cholecystokinin B receptor	2.04E-01	-1.07	7.50E-15	-1.63	4.40E-01	1.05
A_52_P723694	AK080458	7 days neonate cerebellum cDNA, RIKEN full-	2.69E-01	1.28	3.27E-22	-3.54	3.88E-01	-1.30
A_51_P157193	Rhoj,TCL,Arhj,TC10L,AV	ras homolog gene family, member J	6.10E-01	1.02	6.62E-07	1.69	5.40E-01	1.05
A_51_P343833	Traf1,4732496E14Rik	Tnf receptor-associated factor 1	5.69E-01	-1.04	5.00E-05	-1.51	9.39E-01	-1.01
A_52_P222052	D830013H23Rik	RIKEN cDNA D830013H23 gene	5.04E-07	1.81	2.43E-01	1.39	6.36E-01	1.08
A_52_P344978	NAP101564-1	Unknown	2.62E-01	1.09	4.25E-07	1.82	1.27E-01	-1.17
A_51_P507407	Tatdn3,1500010M24Ril	TatD DNase domain containing 3, mRNA (cD	1.60E-03	1.19	1.60E-04	1.51	3.93E-01	-1.07
A_52_P81533	F730002C09Rik	B6-derived CD11 +ve dendritic cells cDNA, R	5.92E-19	1.73	8.14E-01	1.03	2.06E-01	-1.08
A_51_P203955	Gbp2	guanylate nucleotide binding protein 2	2.77E-01	-1.10	2.11E-09	-1.74	7.36E-01	-1.02
A_52_P285194	Plekhk1,Mbf,RTKN2,B1	Membrane-bound factor MBF1 (Mbf)	1.60E-04	1.42	2.20E-04	2.83	9.82E-01	-1.00
A_52_P424778	Adra1a,[a]1a,Adra1c	adrenergic receptor, alpha 1a	1.01E-01	-1.20	2.30E-04	1.89	7.21E-03	-1.21
A_52_P518352	AK046930	10 days neonate cerebellum cDNA, RIKEN ful	5.00E-05	1.56	5.05E-01	1.24	3.03E-01	1.14
A_51_P351015	Lta,LT,Ltx,Tnfb,LT[a],LT-	lymphotoxin A	4.64E-02	1.12	2.98E-17	1.91	9.22E-01	-1.01
A_51_P185693	Slc2a2,Glut2,Glut-2,AI2	solute carrier family 2 (facilitated glucose tra	6.42E-01	-1.24	2.62E-03	-2.60	3.80E-04	10.08
A_51_P451292	Phactr3,H17739,SCAPIN	MKIAA4224 protein	1.26E-03	1.15	1.80E-07	1.62	1.95E-01	1.05
A_51_P445545	F730047E07Rik,Gm134	RIKEN cDNA F730047E07 gene, mRNA (cDNA	2.40E-04	1.95	9.05E-01	-1.04	1.33E-03	1.66
A_51_P297105	Ucp2,Slc25a8	uncoupling protein 2, mitochondrial	5.03E-02	1.13	8.50E-39	1.78	7.63E-01	1.02
A_52_P600946	0610010D24Rik,Daple,/	RIKEN cDNA 0610010D24 gene	1.03E-02	1.46	1.68E-11	1.65	6.60E-02	-1.15
A_52_P662098	Net1,Net1a,mNET1,AI6	neuroepithelial cell transforming gene 1	8.17E-06	1.26	1.83E-12	2.03	2.30E-01	1.08
A_51_P470079	Il1r2,CD121b,Il1r-2	interleukin 1 receptor, type II	2.45E-02	1.20	2.83E-08	1.61	3.37E-03	-1.26
A_52_P669922	Hamp1,Hamp,Hepc,He	hepcidin antimicrobial peptide	1.81E-01	-1.16	0.00E+00	-12.55	8.40E-08	4.54
A_51_P185593	Dmn,Syn,AI852401,MG	Mus musculus desmuslin (Dmn), transcript v	2.09E-01	1.07	2.01E-08	1.58	8.61E-02	-1.09
A_51_P156857	2010002N04Rik,cI-41,A	expressed sequence AI195350	2.33E-01	1.08	5.32E-20	2.48	5.86E-01	-1.05
A_51_P276715	Olfr1350,MOR222-3,M	olfactory receptor MOR222-3	7.52E-01	-1.18	5.19E-21	-1.76	3.82E-01	1.38
A_52_P51743	AK053800	Mus musculus 0 day neonate eyeball cDNA, I	2.62E-12	1.79	4.22E-01	1.36	6.95E-01	-1.07
A_52_P644613	Sult1b1	sulfotransferase family 1B, member 1	8.48E-02	-1.18	1.09E-06	-1.94	1.75E-01	1.10
A_52_P459509	6430517E21Rik,BB361C	RIKEN cDNA 6430517E21 gene, mRNA (cDNA	1.80E-04	2.50	1.00E+00	1.00	4.10E-01	1.56
A_52_P531838	9830166E18Rik	Early B-cell factor 1 (Ebf1), mRNA	3.00E-05	1.57	3.31E-01	1.84	7.44E-01	1.07
A_52_P38825	XM_133174	Mus musculus similar to lens epithelium-deri	1.48E-01	1.09	1.41E-06	1.74	3.45E-01	-1.06
A_51_P288618	Dusp11,AI481497,2010	dual specificity phosphatase 11 (RNA/RNP cc	1.71E-01	-1.15	5.64E-11	-1.91	5.89E-03	1.15
A_52_P1076091	AK051748	Mus musculus 12 days embryo spinal ganglic	2.45E-07	1.56	1.00E+00	1.00	9.07E-01	1.04
[illegible]	[illegible]	[illegible]	[illegible]	[illegible]	[illegible]	[illegible]	[illegible]	[illegible]

A_52_P59264	Sncaip,SYPH1,BB10459	Synuclein, alpha interacting protein (synphili	1.54E-01	-1.08	4.54E-20	-1.51	5.66E-02	1.07
A_51_P322090	Ovol2,MOVO,Ovo2,M-(	zinc finger protein 339	3.82E-01	1.07	6.68E-06	1.66	9.12E-01	-1.01
A_52_P405873	Hnt,R75390,6230410L2	neurotrimin	4.50E-10	1.92	1.00E+00	1.00	1.00E+00	1.00
A_51_P517051	2410008K03Rik,RP23-4	RIKEN cDNA 2410008K03 gene (2410008K03	1.07E-02	1.22	5.96E-13	1.68	8.75E-01	-1.01
A_52_P155554	Cdc42ep2,Cep2,Borg1,/	CDC42 effector protein (Rho GTPase binding)	3.85E-02	-1.14	3.04E-09	-1.51	9.20E-01	-1.01
A_52_P23379	AV216087,MGC19163	expressed sequence AV216087	1.01E-08	-1.65	2.00E-04	-1.82	4.87E-02	-1.27
A_51_P135491	Cflar,Cash,Flip,MRIT,CL	CASP8 and FADD-like apoptosis regulator	4.78E-02	1.10	1.13E-07	1.62	5.80E-01	-1.02
A_51_P193813	Fga,Fib	fibrinogen, alpha polypeptide	6.24E-01	-1.15	9.44E-03	-3.89	9.00E-05	13.02
A_52_P413940	Phc3,Edr3,HPH3,E0300	Polyhomeotic-like 3 (Drosophila) (Phc3), mRI	1.89E-07	-1.26	3.78E-01	-1.73	1.08E-01	1.12
A_52_P445746	Plod2,LH2,Plod-2,D530	Procollagen lysine, 2-oxoglutarate 5-dioxyge	7.02E-01	1.05	2.70E-04	1.72	2.73E-03	-1.22
A_52_P605783	AJ543404,EC1-V2R,EC1	EC1-V2R pheromone receptor	5.63E-01	-1.22	2.50E-04	-1.51	5.80E-01	-1.13
A_51_P268069	Six1,BB138287	sine oculis-related homeobox 1 homolog (Dr	8.34E-07	-1.63	1.82E-01	-1.37	2.00E-01	1.21
A_51_P183414	Guca2b,Ugn,Gcap2,AV(	guanylate cyclase activator 2b (retina)	8.63E-01	1.02	4.00E-05	-3.28	9.52E-01	-1.01
A_51_P262453	Ubr2,AI462103,AW540	Ubiquitin protein ligase E3 component n-rec	3.39E-01	1.11	2.97E-06	1.81	2.51E-01	-1.20
A_52_P867156	AK047784	Adult male corpus striatum cDNA, RIKEN full-	5.87E-18	1.58	4.37E-03	-1.28	2.54E-01	1.35
A_51_P438805	Txnip,THIF,VDUP1,mVD	Thioredoxin interacting protein, mRNA (cDNA	2.02E-19	2.03	3.69E-11	2.11	4.33E-01	1.06
A_52_P134762	Ifi203	Interferon activated gene 203 (Ifi203), mRNA	1.22E-10	-1.76	2.35E-02	-1.40	7.55E-03	-1.19
A_51_P509432	D030035A18Rik	DENN/MADD domain containing 1A (Dennd1	2.35E-08	1.53	9.29E-01	-1.02	2.75E-01	1.30
A_51_P358792	Zfp189,BB131811,C430	RIKEN cDNA C430015I23 gene	8.60E-03	1.23	5.05E-13	1.53	4.07E-01	-1.09
A_52_P27371	AK039022	Adult male spinal cord cDNA, RIKEN full-leng	6.63E-06	1.86	2.05E-01	1.35	2.03E-01	1.20
A_51_P100625	Apon,AI195275,D10Ucl	DNA segment, Chr 10, University of Californi	4.06E-01	1.49	1.29E-18	-8.88	1.00E-05	14.39
A_51_P275147	AK046329	Mus musculus adult male corpora quadrigem	1.13E-12	-1.72	7.60E-04	-1.77	7.85E-02	-1.26
A_51_P417651	Arrdc2,Ilad1,4632416I0	Arrestin domain containing 2, mRNA (cDNA c	3.24E-20	1.67	0.00E+00	2.60	1.49E-02	-1.13
A_51_P126620	ENSMUST00000062144	OLFACTORY RECEPTOR GA_X6K02T2PBJ9-63	9.49E-01	-1.01	1.00E-05	-2.55	8.79E-01	1.04
A_51_P502599	Cyp2c40,AI265721,AI6	cytochrome P450, family 2, subfamily c, poly	9.06E-01	-1.02	4.90E-23	-13.46	2.72E-03	5.81
A_52_P1083611	AK034974	Mus musculus 12 days embryo embryonic bo	1.93E-25	2.11	1.00E+00	1.00	5.40E-01	1.32
A_51_P109548	2510042O18Rik	PREDICTED: chromosome 10 open reading fr	4.29E-01	1.09	2.29E-12	1.87	5.47E-02	-1.09
A_52_P88916	AA438147	Mus musculus 16 days embryo lung cDNA, R	3.19E-02	1.29	9.15E-13	1.85	8.87E-01	1.01
A_52_P514279	Olfr318,MOR285-1,RP2	Mus musculus olfactory receptor MOR285-1	9.98E-01	1.00	4.80E-04	-2.45	6.43E-01	1.10
A_52_P811874	Erh,Mer,Prei1,MGC103	Enhancer of rudimentary homolog (Drosophi	3.10E-04	1.31	9.24E-02	1.64	8.20E-01	-1.02
A_51_P381409	Vil1,Vil	villin	8.15E-02	-1.12	4.19E-12	-1.67	3.47E-01	-1.07
A_52_P100381	LOC382639	similar to RP58 protein	8.94E-01	-1.02	3.40E-04	1.74	9.94E-01	1.00
A_52_P553820	NAP030172-1	Unknown	3.18E-01	-1.09	4.87E-14	1.81	1.49E-01	-1.16
A_52_P344354	4930535B03Rik,AL0228	PREDICTED: hypothetical protein LOC75137	1.43E-01	1.38	1.56E-07	3.62	4.95E-02	-1.28
A_51_P508491	Gm1653	Adult male testis cDNA, RIKEN full-length enr	8.00E-05	1.54	1.00E+00	1.00	1.37E-01	2.36
A_52_P843565	Odz3,Odz1,Ten-m3,mK	ODZ3 (Odz3)	9.94E-09	1.60	5.32E-01	-1.06	3.14E-01	1.18
A_52_P286098	Epb4.1l4b,Ehm2,AA589	erythrocyte protein band 4.1-like 4b	4.02E-03	1.34	5.43E-06	1.65	1.14E-01	-1.11
A_51_P107099	Osbpl8,ORP-8,AA53697	Oxysterol binding protein-like 8 (Osbpl8), tra	2.89E-02	-1.21	1.00E-05	-2.10	3.23E-01	1.10
A_52_P506930	Plcb1,Plcb,AI132408,m	phospholipase C, beta 1	1.63E-03	1.17	4.90E-04	-1.55	7.31E-01	-1.05
A_51_P432641	Cxcl10,C7,IP10,CRG-2,I	chemokine (C-X-C motif) ligand 10	6.00E-05	-1.43	7.28E-13	-1.90	8.35E-02	-1.21
A_51_P122170	Rdh7,CRAD2,AI194929	retinol dehydrogenase 7	3.72E-01	1.16	1.50E-04	-4.45	9.20E-04	3.38
A_52_P955808	Spon1,AI666765,AW45	Spondin 1, (f-spondin) extracellular matrix pr	1.18E-09	1.32	1.16E-02	1.66	2.21E-01	1.17
A_51_P437426	Lrrc33,Lrcc33,MGC368	RIKEN cDNA E430025L02 gene	1.21E-19	1.62	6.06E-18	2.03	3.89E-01	1.09
A_52_P255618	1810029B16Rik	RIKEN cDNA 1810029B16 gene, mRNA (cDNA	0.00E+00	2.07	3.65E-01	-1.69	1.35E-01	-1.26
A_51_P157462	Rgn,SMP30,AI265316	regucalcin	1.38E-03	-1.26	7.49E-27	-2.71	3.42E-01	1.78
A_52_P382802	1700102P08Rik,AI5932	RIKEN cDNA 1700102P08 gene	2.01E-01	1.14	7.91E-06	-1.83	7.28E-01	-1.05
A_51_P158678	A830059I20Rik,AB0415	RIKEN cDNA A830059I20 gene	2.81E-02	-1.12	4.42E-12	-1.54	3.04E-02	-1.09
A_51_P113242	Dnchc2,DHC2,DHC1b,D	Strain C57BL/6 dynein cytoplasmic heavy cha	2.70E-04	1.90	1.00E+00	1.00	1.00E+00	1.00
A_51_P368496	6530411B15Rik,AI4635	RIKEN cDNA 6530411B15 gene	9.98E-03	-1.10	5.93E-08	-1.56	9.47E-01	1.01
A_52_P402577	Dnajc10,JPDI,ERdj5,D2	RIKEN cDNA 1200006L06 gene	1.68E-01	1.10	1.00E-04	1.53	6.20E-04	-1.23
A_51_P371710	Tlr7	toll-like receptor 7	8.52E-06	1.30	1.12E-27	2.22	8.06E-01	1.02
A_51_P344797	Dmd,mdx,pke,Dp427,D	Dystrophin, muscular dystrophy (Dmd), mRN	5.11E-08	1.53	9.37E-01	-1.04	8.78E-01	1.06
A_52_P222073	Ccbp2,D6,CCR9,CCR10,	expressed sequence AI464239	5.69E-01	-1.04	1.78E-11	1.92	1.26E-01	1.10
A_51_P362483	Slc43a1,PB39,Pov1,R00	Lat3 mRNA for L-type amino acid transporter	4.09E-01	1.28	2.13E-11	2.78	5.17E-01	-1.10
A_52_P356204	LOC329416,Daip2	nitric oxide synthase trafficker	7.58E-01	-1.03	3.68E-19	1.85	6.80E-03	-1.30
A_51_P400335	Terf2ip	telomeric repeat binding factor 2, interacting	8.74E-01	1.01	1.28E-09	1.60	4.33E-01	-1.05
A_51_P362818	Nalp6,Pypaf5,AI504961	NACHT, leucine rich repeat and PYD contain	1.78E-01	1.11	4.89E-23	2.11	6.92E-01	1.07
A_51_P435617	Vmd2l3	Vitelliform macular dystrophy 2-like 3 (Vmd2	9.89E-08	1.86	1.84E-03	1.90	4.08E-03	1.51
A_51_P444447	Cebpd	CCAAT/enhancer binding protein (C/EBP), de	1.29E-10	1.56	1.51E-07	2.33	9.13E-01	-1.01
A_51_P411874	Ankhd1,MASK,AA5714(	Ankyrin repeat and KH domain containing 1,	3.00E-05	-1.70	6.22E-01	-1.10	4.52E-01	-1.13
A_52_P505944	Samd8,MGC118303,11	Sterile alpha motif domain containing 8 (Sam	3.78E-02	1.12	1.47E-19	1.56	5.36E-01	1.07
A_52_P71105	Sertad3,Rbt1	replication protein binding trans-activator	1.28E-28	-1.54	3.84E-02	-1.40	4.44E-01	-1.04
A_52_P273891	Fmo2,AW107733,MGC	flavin containing monooxygenase 2	8.19E-02	-1.13	4.52E-10	3.86	3.70E-01	-1.16
A_51_P402378	Arfl4,ARL6,AW456149,	RIKEN cDNA 1110036H21 gene	1.72E-06	1.31	5.80E-24	1.56	3.44E-01	-1.04
A_51_P253270	Rpgrip1,AA415034,061	Retinitis pigmentosa GTPase regulator intera	9.00E-05	1.30	2.43E-01	-1.61	6.75E-01	1.07
A_52_P528926	Zfp287,Skat2,SKAT-2,Zr	Zinc finger protein 287 (Zfp287), mRNA	3.24E-23	-1.84	3.06E-02	-1.41	3.15E-01	-1.06
A_52_P679105	Prss23,Spuve,AI790574	RIKEN cDNA 2310046G15 gene	9.34E-02	1.11	1.15E-07	1.58	8.92E-01	1.01
A_52_P117294	9030227G01Rik	RIKEN cDNA 9030227G01 gene	9.49E-29	1.85	1.18E-01	1.33	1.58E-02	1.21
A_51_P184672	Tcte3,LC2,Tcte-3,Tctex	t-complex-associated testis expressed 3	9.10E-01	-1.02	2.94E-11	1.68	2.17E-02	-1.17
A_51_P125965	2210022J03Rik	Apoptosis, caspase activation inhibitor (Aven	2.81E-06	1.50	1.00E-05	1.25	3.28E-01	1.12
A_52_P472353	9330187F13Rik	ODZ2 (Odz2)	1.40E-04	1.34	8.00E-05	1.56	3.33E-01	1.07
A_51_P400269	Slc38a5,SN2,JM24,C81	expressed sequence C81234	2.85E-03	1.18	1.03E-21	-2.46	1.21E-02	1.18
A_52_P574291	Chic2,BTL,1700081B18	Cysteine-rich hydrophobic domain 2 (Chic2),	3.20E-12	1.35	1.09E-03	1.57	8.28E-01	1.01
A_52_P891818	Magi1,AIP3,BAP1,WWF	Putative membrane-associated guanylate kir	1.55E-06	1.44	2.60E-04	-1.70	1.63E-01	1.27
A_52_P1020831	Zfp292,Zn-15,Zn-16,AI4	Zinc finger protein 292, mRNA (cDNA clone II	2.00E-05	1.52	8.95E-01	1.06	9.87E-01	-1.00
A_52_P127069	Sgk3,Cisk,2510015P22F	serum/glucocorticoid regulated kinase 3	8.90E-02	-1.09	3.09E-19	1.88	9.16E-01	1.01
A_52_P361534	Wnt3,Int-4,Wnt-3	wingless-related MMTV integration site 3	1.48E-01	1.12	6.59E-17	1.73	7.81E-01	-1.02
[illegible]	[illegible]	[illegible]	[illegible]	[illegible]	[illegible]	[illegible]	[illegible]	[illegible]

A_52_P330201	2310004L02Rik,C03004	RIKEN cDNA 2310004L02 gene	4.27E-01	-1.07	1.91E-06	1.64	1.25E-01	-1.14
A_52_P955856	C430019N01Rik	Anthrax toxin receptor 2, mRNA (cDNA clone	6.34E-01	1.05	1.33E-09	1.94	3.14E-01	1.13
A_51_P215147	Carf,Als2cr8	Calcium response factor (Carf), mRNA	7.67E-08	1.93	4.26E-01	1.56	4.34E-06	1.33
A_51_P235139	Kbtbd5,2310024D23Rik	PREDICTED: kelch repeat and BTB (POZ) dom	8.61E-01	-1.02	1.75E-24	1.64	3.68E-01	-1.10
A_52_P676877	Sbf2,Mtmr13,mMTMH	Myotubularin related protein 13, mRNA (cDN	7.00E-05	1.41	4.26E-07	1.82	4.42E-01	1.07
A_52_P74144	4933437K13Rik	RIKEN cDNA 4933437K13 gene, mRNA (cDNA	2.00E-05	1.20	3.41E-10	-1.50	6.96E-01	-1.03
A_52_P554451	1300017J02Rik	RIKEN cDNA 1300017J02 gene	1.47E-01	-1.33	2.00E-05	-2.02	9.94E-02	1.46
A_52_P98452	NAP123849-1	Unknown	1.64E-11	-1.87	7.07E-01	1.12	1.00E-05	1.65
A_52_P523835	Nfe2l2,Nrf2,AI194320	Nuclear factor, erythroid derived 2, like 2, m	6.06E-08	1.85	7.26E-06	2.28	6.89E-01	-1.03
A_52_P1115511	6030422H21Rik	FK506 binding protein 5, mRNA (cDNA clone	1.49E-21	3.19	3.78E-10	3.76	3.13E-01	1.11
A_52_P625296	Vav2,AI847175,281004	Vav2 oncogene (Vav2), mRNA	1.34E-02	-1.10	1.31E-06	1.50	1.36E-02	1.10
A_51_P332506	V1rg5	Mus musculus vomeronasal 1 receptor, G5 (V	5.05E-06	-2.16	1.00E+00	1.00	2.30E-01	-1.53
A_51_P215380	Dscam,4932410A21Rik	Down syndrome cell adhesion molecule (Dsc	8.58E-08	1.73	7.73E-01	1.07	4.68E-01	1.13
A_52_P715406	Nol4,Gm1262,1700013	Nucleolar protein 4 (Nol4), mRNA	7.00E-05	1.68	1.00E+00	1.00	1.00E+00	1.00
A_52_P171160	D930030D11Rik	RIKEN cDNA D930030D11 gene	2.80E-03	1.28	6.00E-05	1.53	9.83E-01	1.00
A_52_P320109	C530014P21Rik	Ephrin A5 (Efna5), transcript variant 2, mRNA	9.27E-07	3.00	7.99E-01	1.12	6.00E-01	-1.27
A_52_P599014	Kif1b,D4Mil1e,AI44821	Mus musculus kinesin family member 1B (Kif	4.04E-01	1.06	9.75E-07	1.82	8.60E-01	-1.01
A_51_P155873	Ppp1r3g,1600032L17Ri	PREDICTED: hypothetical protein LOC76487	1.04E-02	1.17	3.68E-29	1.57	4.67E-01	-1.04
A_52_P201934	E330009E22Rik	Ankyrin repeat domain 17, mRNA (cDNA clor	4.50E-04	1.54	1.00E+00	1.00	9.51E-01	-1.01
A_51_P406509	4933428P19Rik	RIKEN cDNA 4933428P19 gene	8.77E-07	1.35	4.02E-07	2.51	1.27E-01	-1.15
A_52_P1163837	Capn2,Capa2,Capa-2,AI	Calpain 2 (Capn2), mRNA	2.26E-01	1.06	2.00E-04	1.80	3.93E-01	1.07
A_52_P5454	Cd248,Tem1,Cd164l1,A	CD164 sialomucin-like 1	9.90E-01	-1.00	2.13E-11	-1.65	5.16E-07	1.20
A_52_P314421	Fbxo30,1700026A16Rik	F-box protein 30, mRNA (cDNA clone MGC:4	8.00E-05	-1.65	6.50E-04	-1.82	6.07E-01	-1.09
A_51_P464182	Mat1a,Ams,MAT,SAMS	methionine adenosyltransferase I, alpha	7.74E-01	1.06	2.83E-07	-5.32	6.58E-06	7.56
A_51_P385190	BC057371,B930030J24	Zinc finger, MYND domain containing 12 (Zm	2.80E-22	1.52	3.70E-04	1.66	2.72E-01	1.11
A_51_P295192	Nfkbia,Nfkbi,AI462015	nuclear factor of kappa light chain gene enha	6.43E-37	1.79	7.41E-11	2.21	9.14E-01	-1.01
A_51_P310109	Olfr52,IE6,ORL446,MO	olfactory receptor 52	8.24E-01	-1.05	8.07E-07	-2.04	3.38E-01	-1.29
A_52_P691960	Suhw4,ZNF634,BC0271	Suppressor of hairy wing homolog 4 (Drosop	8.93E-02	-1.32	4.91E-06	-2.14	3.72E-01	1.11
A_51_P315890	Kcnk6,Toss,Twik2,D7Er	TWIK-2 two-pore-domain K+ channel (Kcnk6)	1.00E-05	1.28	1.40E-07	1.58	4.11E-01	-1.06
A_51_P442759	Arhgef19,WGEF,60304	RIKEN cDNA 6430573B13 gene	2.11E-01	1.05	7.50E-14	-1.76	8.99E-01	-1.02
A_51_P170807	Map3k6,MAPKKK6	mitogen-activated protein kinase kinase kina	1.09E-06	1.27	4.95E-15	3.16	5.62E-01	1.04
A_51_P133911	Olfr1043,MOR185-7	Mus musculus olfactory receptor MOR185-7	4.54E-01	-1.13	4.12E-07	-1.91	4.78E-01	1.18
A_52_P762514	Sucla2,4930547K18Rik	ATP-specific succinyl-CoA synthetase beta su	5.57E-01	1.08	1.80E-04	2.63	3.07E-01	-1.09
A_52_P402431	5430437A18Rik	Putative SH3BGR protein (SH3BGR gene)	1.69E-01	-2.11	1.00E+00	1.00	6.03E-07	4.26
A_51_P325836	Hpxn,hx	hemopexin	2.80E-01	1.11	0.00E+00	-6.74	5.03E-02	2.86
A_52_P105707	AK080632	10 days neonate cortex cDNA, RIKEN full-len	3.84E-13	1.84	1.00E+00	1.00	7.85E-01	-1.17
A_51_P266883	Mup4,Mup-4	major urinary protein 4	8.63E-01	-1.03	0.00E+00	-18.44	2.40E-01	1.99
A_51_P250217	Pck1,PEPCK,Pck-1,AI26	phosphoenolpyruvate carboxykinase 1, cytos	7.28E-01	-1.09	1.24E-08	-3.61	4.00E-05	17.74
A_51_P363400	Prg2,MBP,mMBP,mMB	proteoglycan 2, bone marrow	4.95E-01	1.25	3.55E-14	-1.61	1.47E-02	-1.80
A_51_P519354	Ryr3	Mus musculus ryanodine receptor 3 (Ryr3), r	1.44E-25	1.85	4.65E-03	1.91	3.14E-01	1.14
A_52_P288470	Mgea5,Hy5,AA408215,	Meningioma expressed antigen 5 (hyaluronic	6.10E-14	1.64	5.96E-02	1.41	9.51E-02	1.14
A_52_P899611	Nrxn3,6332407J11,D12	Neurexin III, mRNA (cDNA clone MGC:67582	3.60E-04	1.56	7.19E-01	1.08	1.02E-01	1.24
A_52_P963431	Ehbp1,NACSIN,AF4246	EH domain binding protein 1 (Ehbp1), mRNA	4.00E-10	1.57	5.11E-01	1.13	2.00E-01	1.21
A_51_P382512	Zfp418,MGC31575,A23	RIKEN cDNA A230102I05 gene	6.00E-09	-1.61	5.67E-03	-1.34	2.83E-02	-1.09
A_52_P276986	Dclre1b,mSNM1B,AI45	DNA cross-link repair 1B, PSO2 homolog (S. c	3.10E-04	1.28	6.29E-11	1.60	3.33E-01	-1.14
A_51_P491017	Gnptg,Tce7,Mdcp1,AU	M6PR domain containing protein 1 precursor	4.81E-01	1.03	1.50E-04	1.52	7.36E-01	1.02
A_51_P238576	Cyp4a14,AI314743,MG	cytochrome P450, family 4, subfamily a, poly	7.26E-01	-1.03	8.18E-32	-15.34	4.41E-03	1.62
A_52_P787975	AK076568	Activated spleen cDNA, RIKEN full-length enr	5.77E-01	-1.11	3.00E-05	-1.78	1.78E-01	1.21
A_52_P642136	2610209C05Rik,49334	Zinc and ring finger 2 (Znrf2), mRNA	9.65E-01	-1.00	1.73E-09	1.62	4.24E-01	-1.08
A_51_P350453	Pdk4,AV005916	pyruvate dehydrogenase kinase, isoenzyme 4	5.77E-01	1.05	6.43E-18	1.78	2.51E-02	-1.20
A_51_P355040	Nsun3,AU022521,6720	expressed sequence AU022521	1.92E-01	1.08	8.84E-09	1.55	3.47E-01	-1.10
A_51_P243559	4833431D13Rik	Mus musculus hypothetical protein 4833431	5.96E-15	2.29	1.86E-01	-1.33	1.83E-01	1.69
A_51_P389120	6130401L20Rik	PREDICTED: hypothetical protein LOC75740	3.10E-04	1.55	5.09E-01	1.34	5.30E-01	1.12
A_52_P681771	Pnpla2,TTS-2.2,061003	RIKEN cDNA 0610039C21 gene	3.54E-03	1.12	1.44E-18	1.75	7.15E-01	1.03
A_51_P236267	St8sia4,PST,PST-1,Siat8	Mus musculus sialyltransferase 8 (alpha-2, 8-	1.83E-01	-1.09	3.02E-13	-1.50	9.69E-01	-1.01
A_51_P341498	Ankrd39,9130416N05R	Ankyrin repeat domain 39 (Ankrd39), mRNA	4.45E-02	1.08	1.37E-18	1.50	6.97E-01	1.02
A_51_P155323	Hc,C5,He	hemolytic complement	5.17E-01	-1.05	2.77E-15	-1.86	6.20E-01	-1.04
A_51_P353895	Sult1c2,ST1C1,Sult1c1,	sulfotransferase family, cytosolic, 1C, membe	7.72E-01	-1.04	5.14E-07	1.55	5.82E-01	1.05
A_52_P454430	AK081094	PREDICTED: Mus musculus expressed sequer	1.25E-18	1.81	7.42E-02	1.65	2.71E-01	-1.15
A_52_P430194	Smox,PAO,SMO,PAOh1	Mus musculus 0 day neonate eyeball cDNA,	8.79E-27	1.61	5.50E-04	1.26	6.13E-01	1.07
A_52_P550375	Ank2,Ank-2,AI835472,A	Ankyrin 2, brain, mRNA (cDNA clone MGC:67	1.00E-04	1.58	3.86E-03	2.07	6.87E-01	-1.09
A_51_P152023	1200016G03Rik,23100	Mus musculus RIKEN cDNA 1200016G03 gen	1.21E-01	1.08	1.16E-08	1.70	2.99E-01	1.05
A_52_P883243	Smug1,C85220,120001	Mus musculus adult male corpora quadrigem	6.02E-03	1.24	4.37E-06	1.56	6.29E-02	1.17
A_52_P27075	0610040B10Rik	PREDICTED: hypothetical protein LOC67672	1.70E-03	1.30	6.41E-12	1.84	4.08E-02	-1.15
A_52_P370031	AK043317	7 days neonate cerebellum cDNA, RIKEN full-	4.43E-01	-1.12	8.85E-21	1.79	6.45E-02	-1.11
A_51_P333324	Slc1a3,Gmt1,Eaat1,GLA	Solute carrier family 1 (glial high affinity gluta	2.11E-09	1.59	2.31E-01	1.45	8.68E-01	1.02
A_51_P144014	Gdap5	Dishevelled associated activator of morphog	9.51E-01	-1.00	9.51E-06	-1.59	2.72E-01	1.11
A_52_P377160	Galnt6,AW047994,Gal	UDP-N-acetyl-alpha-D-galactosamine:polype	8.37E-03	-1.13	4.60E-04	-1.78	8.46E-01	1.01
A_52_P605556	4930519N16Rik,MGC2	RIKEN cDNA 4930519N16 gene	1.55E-01	1.08	2.44E-09	1.51	8.89E-01	1.01
A_51_P413866	H2-Bf,B,Bf,FB,AI195813	histocompatibility 2, complement componen	2.35E-01	1.44	4.36E-07	-1.84	2.40E-02	2.17
A_52_P996561	Pja2,AI447901,AL0227	Praja 2, RING-H2 motif containing (Pja2), tra	4.27E-09	1.72	1.00E+00	1.00	7.97E-01	1.08
A_51_P382347	Nphp3,pcy,AI550417,C	Nephronophthisis 3 (adolescent) (Nphp3), m	2.20E-07	1.79	1.16E-01	1.54	7.64E-01	1.07
A_52_P947137	Rnd1,Arhs,A830014L09	0 day neonate thymus cDNA, RIKEN full-leng	9.58E-01	1.01	9.00E-05	1.54	4.00E-01	-1.07
A_52_P671965	NAP030023-1	Unknown	8.99E-01	1.05	2.40E-04	-2.80	9.18E-01	1.04
A_52_P233411	3830422K02Rik,MGC1	RIKEN cDNA 3830422K02 gene	2.11E-01	-1.07	1.34E-24	-3.00	2.36E-02	-1.16
A_52_P35470	Bai3,MGC107444,A830	Brain-specific angiogenesis inhibitor 3, mRN	2.71E-07	1.61	1.68E-01	-1.61	2.53E-01	1.10
A_51_P516219	Rpl5,U21RNA,AI315274	Ribosomal protein L5 (Rpl5), mRNA	3.00E-05	1.30	1.60E-04	1.64	3.03E-01	1.23
[illegible]	[illegible]	[illegible]	[illegible]	[illegible]	[illegible]	[illegible]	[illegible]	[illegible]

A_51_P363947	Cdkn1a,P21,CDKI,CIP1,S	cyclin-dependent kinase inhibitor 1A (P21)	1.90E-04	1.43	0.00E+00	2.90	4.07E-01	-1.08
A_52_P1155986	Elmo1,CED-12,AI17300	Engulfment and cell motility 1, ced-12 homol	4.00E-04	1.23	1.68E-03	1.51	9.42E-01	1.01
A_52_P496726	Rasd1,Dexras1	RAS, dexamethasone-induced 1	9.00E-05	1.44	9.28E-06	1.69	3.47E-01	1.08
A_52_P900390	E030004H24Rik	RIKEN cDNA 5830434P21 gene, mRNA (cDNA	4.69E-07	1.48	1.19E-03	2.12	5.44E-01	-1.15
A_52_P92037	Uox,AI663847	urate oxidase	8.00E-01	-1.02	2.39E-22	-1.97	1.78E-01	1.12
A_52_P386037	Hbxap	Mus musculus hypothetical protein Hbxap (H	6.49E-02	1.15	3.96E-10	2.22	5.16E-01	-1.09
A_52_P803797	AK085108	Mus musculus 13 days embryo lung cDNA, R	3.49E-15	1.53	8.80E-03	-1.30	1.77E-01	1.34
A_51_P209258	AU022855	PREDICTED: RIKEN cDNA A630039F14 [Mus r	4.07E-01	1.09	2.60E-07	1.57	2.92E-01	1.23
A_52_P1107474	B230104C14Rik	Adult male corpora quadrigemina cDNA, RIKE	3.44E-13	-1.57	9.15E-01	1.01	5.82E-01	-1.04
A_52_P508163	NAP021083-001	Unknown	9.45E-03	1.33	9.69E-06	2.33	4.20E-04	-1.46
A_52_P666442	TC975013	AY167973 very large inducible GTPase-1 {Mu	9.00E-05	-1.24	1.39E-02	-1.71	4.25E-02	-1.12
A_51_P213223	Galntl4,BC024988,MGC	UDP-N-acetyl-alpha-D-galactosamine:polype	1.50E-10	1.61	4.66E-01	1.23	9.38E-01	-1.01
A_52_P1091831	4933425L03Rik,Nag	PREDICTED: neuroblastoma-amplified protei	1.38E-11	8.69	1.00E+00	1.00	1.00E+00	1.00
A_52_P1018726	4921515E04Rik	Mus musculus adult male testis cDNA, RIKEN	1.10E-04	-1.52	5.01E-02	-1.52	1.00E-02	1.22
A_52_P481770	XM_144609	Mus musculus similar to High mobility group	9.68E-01	-1.00	1.20E-04	1.53	6.00E-02	-1.10
A_51_P371892	2700046A07Rik,A33004	PREDICTED: hypothetical protein LOC78449 [	3.80E-04	1.31	1.00E-05	1.56	8.56E-01	-1.03
A_52_P639362	4930570G19Rik,C63001	Adult male hippocampus cDNA, RIKEN full-le	2.00E-05	1.25	6.89E-06	1.63	9.96E-01	1.00
A_52_P358951	Zfp26,Zfp70,mkr-3,KRA	Mus musculus mRNA for mszf14, partial cds.	1.10E-01	1.24	4.60E-04	1.72	1.03E-01	-1.12
A_51_P217498	Slc2a4,Glut4,Glut-4	solute carrier family 2 (facilitated glucose tra	4.63E-01	-1.10	5.64E-07	1.57	4.07E-01	1.07
A_52_P169838	Polr2a,220kDa,Rpo2-1	Polymerase (RNA) II (DNA directed) polypept	2.59E-11	1.60	6.00E-05	1.68	1.26E-01	1.08
A_52_P13680	C920021L13Rik	CDNA sequence BC028528 (BC028528), mRN	4.82E-02	1.16	1.40E-04	1.79	5.37E-01	1.06
A_52_P23987	Set,AA407739,StF-IT-1,I	SET translocation	7.73E-01	1.03	1.00E-05	1.84	2.22E-01	-1.05
A_51_P340027	Pappa,PAG1,PAPP-A,IG	Pregnancy-associated plasma protein A (Pap	6.48E-01	1.06	5.29E-06	-1.55	4.36E-01	1.07
A_51_P514712	Parp14,BC021340,KIAA	Poly (ADP-ribose) polymerase family, membe	1.91E-06	-1.26	4.27E-10	-1.63	4.44E-01	-1.05
A_52_P303803	D9Ertd809e,BC035275,	PREDICTED: Mus musculus RIKEN cDNA B130	4.55E-11	1.90	3.37E-01	1.51	9.47E-01	-1.02
A_52_P14666	NAP030651-1	Unknown	5.45E-01	1.03	2.00E-05	1.74	5.12E-01	-1.06
A_51_P317214	Gloxd1,BC034099,MGC	cDNA sequence BC034099	2.64E-02	1.28	4.79E-06	1.56	7.76E-01	1.02
A_52_P316765	Alox12b,Aloxe2,e-LOX2	arachidonate 12-lipoxygenase, 12R type	5.15E-02	1.09	2.25E-11	1.55	8.92E-01	-1.01
A_51_P134452	Edg7,lpA3	endothelial differentiation, lysophosphatidic	9.64E-01	-1.01	4.20E-04	2.31	4.81E-01	-1.09
A_52_P1003754	AK045754	Mus musculus adult male corpora quadrigem	6.22E-03	1.21	3.99E-10	2.55	4.60E-02	1.16
A_52_P73385	Fas,lpr,APT1,CD95,APO	Fas (TNF receptor superfamily member) (Fas	7.63E-03	1.36	7.74E-07	2.61	8.70E-01	1.02
A_51_P171531	2900006K08Rik,AI4277	Aldehyde dehydrogenase family 6, subfamily	3.64E-01	1.04	2.13E-06	1.64	5.85E-01	1.05
A_52_P8286	2810428J06Rik	10, 11 days embryo whole body cDNA, RIKEN	1.76E-10	1.54	1.00E+00	1.00	4.59E-02	1.53
A_52_P843948	AK079718	0 day neonate thymus cDNA, RIKEN full-leng	3.38E-18	1.60	1.32E-01	1.21	7.91E-01	1.04
A_51_P385237	Dguok,dGK	Deoxyguanosine kinase 3	5.51E-09	1.94	1.00E+00	1.00	7.62E-01	-1.13
A_51_P178828	Mbl2,MBL,L-MBP,MBL-	mannose binding lectin, serum (C)	2.76E-02	-1.28	3.58E-33	-2.26	2.25E-03	1.22
A_52_P67758	Senp7,AI790676,mKIAA	SUMO1/sentrin specific peptidase 7 (Senp7),	1.38E-02	1.32	1.48E-13	1.91	4.74E-01	1.08
A_51_P251465	Exosc1,AI447561,26100	RIKEN cDNA 2610104C07 gene	9.40E-01	1.00	5.39E-22	1.51	7.69E-01	-1.02
A_51_P462428	Galntl2,4631401E18Rik	UDP-N-acetyl-alpha-D-galactosamine:polype	4.70E-03	1.11	3.94E-06	1.52	3.78E-01	-1.10
A_52_P542612	Ephb4,Htk,MDK2,Myk1	Eph receptor B4	8.19E-02	-1.12	4.60E-04	-1.52	6.75E-02	1.11
A_52_P147644	5430401H09Rik,AW987	6 days neonate head cDNA, RIKEN full-length	6.60E-01	1.06	5.09E-08	1.95	3.03E-01	1.13
A_51_P273790	Pbsn,PB,Prbs	probasin	7.26E-03	-1.23	4.00E-05	-2.14	1.59E-02	1.14
A_51_P520198	Vps37a,AW261445,D8E	DNA segment, Chr 8, ERATO Doi 531, expres	1.97E-08	-1.54	1.00E+00	1.00	6.36E-01	1.25
A_51_P297195	4930543L23Rik	RIKEN cDNA 4930543L23 gene	2.40E-03	1.73	5.88E-07	2.60	7.24E-01	-1.03
A_52_P526607	A730017C20Rik	RIKEN cDNA A730017C20 gene, mRNA (cDNA	1.31E-15	1.60	3.52E-18	1.59	4.71E-02	1.15
A_52_P526677	4932442K08Rik,AW049	RIKEN cDNA 4932442K08 gene, mRNA (cDNA	1.00E-05	1.62	1.31E-14	1.96	1.36E-01	-1.09
A_52_P14569	1700101I11Rik	Gamma-aminobutyric acid (GABA(A)) recepto	3.61E-01	1.06	4.52E-12	1.54	4.23E-01	-1.07
A_51_P383599	Golgb1,AU042952,KIAA	PREDICTED: Mus musculus golgi autoantigen	3.02E-03	1.39	3.42E-09	1.86	4.98E-01	-1.12
A_51_P224493	Pdzk4,LU1,Xlu,PDZD4,P	PDZ domain containing 4 (Pdzk4), mRNA	5.17E-03	-1.15	3.18E-07	-2.35	2.41E-01	1.16
A_51_P509627	Rere,ARG,ARP,Atr2,DNI	MKIAA0458 protein	2.17E-31	2.89	1.00E+00	1.00	1.00E+00	1.00
A_51_P266644	Sesn1,PA26,SEST1,AU0	Sestrin 1, mRNA (cDNA clone MGC:118148 IM	2.13E-01	1.07	2.80E-04	1.70	1.25E-01	-1.11
A_52_P1076565	DXImx50e,Jm11,Sfc25,A	Adult male bone cDNA, RIKEN full-length enr	5.31E-01	1.09	4.00E-05	1.90	1.39E-01	1.18
A_52_P593965	Fdps,Fdpsl1,AI256750,N	Mus musculus 13 days embryo male testis cD	5.87E-10	-1.59	3.07E-02	-1.31	7.96E-01	-1.03
A_51_P376838	LOC433658	hypothetical gene supported by AK035802	7.73E-01	1.03	8.48E-25	1.95	1.48E-01	-1.16
A_51_P103706	Cyp2c29,AHOH,Ah-2,AH	cytochrome P450, family 2, subfamily c, poly	2.24E-01	-1.14	3.77E-36	-5.87	3.68E-02	2.85
A_51_P480311	F2,Cf2,FII,Cf-2	coagulation factor II	9.34E-01	-1.01	0.00E+00	-5.18	1.71E-01	2.02
A_52_P119350	4732419C18Rik	PREDICTED: hypothetical protein XP_489255	2.01E-03	1.27	2.10E-04	1.59	1.26E-01	1.12
A_52_P747157	AK038433	Mus musculus adult male hypothalamus cDN	8.29E-01	-1.03	1.77E-09	1.75	1.61E-01	-1.10
A_51_P134574	Col4a3bp,CERT,GPBP,A	Procollagen, type IV, alpha 3 (Goodpasture a	2.68E-02	-1.21	2.00E-04	-1.52	8.55E-01	-1.01
A_51_P142529	Saa4,Saa-4,Saa-5	serum amyloid A 4	7.39E-01	-1.20	1.92E-03	-2.87	1.25E-06	4.36
A_51_P196444	Foxc2,Mfh1,Fkh14,MFH	forkhead box C2	9.98E-06	-1.58	1.44E-01	-1.17	3.34E-03	1.22
A_52_P32886	AI256396	Mus musculus 4 days neonate male adipose	4.55E-03	-1.17	4.88E-06	-1.79	1.31E-01	1.08
A_52_P127012	Ap4s1,AI314282	Adaptor-related protein complex AP-4, sigma	1.42E-01	-1.30	3.62E-09	-1.98	1.31E-01	1.14
A_51_P186547	Pah,AW106920	phenylalanine hydroxylase	2.03E-01	-1.27	2.73E-40	-5.89	4.28E-01	1.68
A_51_P340653	Slc16a4,AW146050,MG	solute carrier family 16 (monocarboxylic acid	1.10E-01	-1.14	9.05E-26	-2.03	8.59E-01	-1.03
A_52_P386754	Blcap,Bc10,AI462828,N	Bladder cancer associated protein homolog (	1.76E-02	-1.27	1.59E-06	-1.65	5.78E-01	1.05
A_52_P431483	A730095J18Rik	Zinc finger protein, subfamily 1A, 2 (Helios) (Z	4.86E-01	-1.03	7.63E-06	1.63	2.26E-01	1.08
A_52_P343455	Tnfrsf13b,Taci,1200009	Mus musculus adult male lung cDNA, RIKEN f	9.19E-01	-1.01	2.58E-07	-1.58	1.01E-01	-1.10
A_51_P433810	Npas4,Nxf,LE-PAS	bHLH-PAS type transcription factor NXF	1.15E-11	-2.11	3.45E-12	-2.65	3.98E-01	1.12
A_51_P421628	Mjd,ATX3,MJD1,Sca3,A	Machado-Joseph disease (spinocerebellar ata	1.87E-02	1.23	1.40E-04	2.17	1.32E-01	1.19
A_52_P268489	Prdm2,Riz,Riz1,Gm1033	PR domain containing 2, with ZNF domain, m	6.38E-16	1.55	1.07E-01	1.32	5.80E-01	1.05
A_52_P139306	LOC546251	similar to RIKEN cDNA 1700029I01	7.51E-01	1.02	1.96E-07	1.57	4.72E-01	-1.09
A_52_P1147388	6820402I19Rik	Ubiquitin-conjugating enzyme E2E 2 (UBC4/5	4.00E-05	1.52	1.40E-04	1.24	4.13E-01	1.15
A_52_P424655	D14Ertd171e,CAST,643	DNA segment, Chr 14, ERATO Doi 171, expre	2.00E-05	1.65	4.97E-10	2.37	1.38E-02	1.11
A_52_P122649	Dmrta1,Dmrt4	doublesex and mab-3 related transcription fa	2.32E-02	1.35	3.07E-20	5.50	4.51E-01	-1.20
A_52_P116919	Ctag3	Mus musculus RIKEN cDNA 4933427I01 gene	4.23E-01	-1.03	2.26E-06	1.53	9.60E-01	1.00
A_51_P370372	Olfr876,MOR161-1	Mus musculus olfactory receptor MOR161-1	3.65E-01	-1.12	3.60E-04	-1.68	6.52E-01	1.07
[illegible]	[illegible]	[illegible]	[illegible]	[illegible]	[illegible]	[illegible]	[illegible]	[illegible]

A_52_P358360	Igh-6,Igm,muH,Igh6,Igh	Immunoglobulin heavy chain 6 (heavy chain (	7.90E-02	-1.12	1.28E-07	-1.51	3.39E-01	-1.09
A_52_P614741	Btbd11,6330404E16Rik	BTB (POZ) domain containing 11 (Btbd11), tr	7.00E-05	1.52	1.00E+00	1.00	2.13E-01	1.44
A_51_P197910	BC013672,MGC19297,9	CDNA sequence BC013672, mRNA (cDNA clo	4.79E-06	2.03	1.00E+00	1.00	4.58E-01	-1.51
A_52_P851862	Nrp1,Nrp,NP-1,Npn1,N	Neuropilin 1 (Nrp1), mRNA	3.00E-05	-1.21	4.00E-05	-1.64	6.98E-01	1.06
A_51_P346704	Sox10,Dom,Sox21	SRY-box containing gene 10, mRNA (cDNA clo	9.38E-02	-1.06	2.00E-05	-1.72	6.24E-01	1.04
A_52_P34058	Klra3,5E6,Nk2,Nk-2,Ly4	killer cell lectin-like receptor, subfamily A, me	9.62E-01	-1.02	2.00E-05	-2.05	9.54E-01	1.02
A_51_P219192	AK090180	Mus musculus 11 days embryo spinal cord cD	2.99E-09	2.12	1.88E-01	1.22	1.00E+00	1.00
A_52_P311148	2410002O22Rik,AI4518	RIKEN cDNA 2410002O22 gene, mRNA (cDNA	2.00E-05	1.22	1.30E-04	1.64	1.08E-02	1.11
A_52_P409746	Serpinc1,At3,At-3,ATIII,	serine (or cysteine) proteinase inhibitor, clad	7.45E-01	1.06	0.00E+00	-42.55	5.36E-02	6.65
A_52_P135455	TC954299	AF302077 neprilysin-like peptidase gamma {I	2.83E-13	-1.58	2.69E-02	-1.35	2.14E-01	-1.09
A_51_P503671	Syt13,AI549909,mKIAA	synaptotagmin 13	6.03E-02	1.10	1.56E-07	-2.33	4.82E-01	-1.06
A_51_P159194	Junb	Jun-B oncogene	3.14E-09	-1.35	7.45E-08	-1.60	7.53E-01	1.04
A_52_P554679	4631402N15Rik	RIKEN cDNA 4631402N15 gene	2.93E-06	1.72	4.72E-01	1.47	4.43E-01	-1.38
A_52_P295822	Robo2,mKIAA1568,260(	MKIAA1568 protein	6.68E-01	-1.05	3.10E-04	2.48	9.73E-02	-1.13
A_52_P227	4930520O04Rik	MRNA similar to RIKEN cDNA 2810026P18 ge	2.17E-03	1.22	4.00E-16	1.80	3.84E-01	1.08
A_52_P843941	Pld1,Pld1a,Pld1b,C8539	Phosphatidylcholine-specific phospholipase D	3.17E-10	1.50	3.62E-12	2.06	9.64E-01	-1.01
A_52_P334593	Ccrl2,E01,CCR11,L-CCR,	chemokine (C-C motif) receptor-like 2	8.78E-01	1.01	6.59E-09	-1.89	2.09E-02	-1.17
A_51_P284177	9030611N15Rik,Akr1c1	RIKEN cDNA 9030611N15 gene	2.31E-02	-1.23	2.97E-34	-2.16	7.48E-01	-1.05
A_52_P78078	5730507A11Rik,943007	Germ cell nuclear factor protein (Nr6a1)	5.79E-02	-1.13	3.10E-06	-2.18	9.10E-02	1.18
A_52_P368690	Gm638	Mus musculus similar to adhesion molecule /	5.27E-01	1.05	2.66E-06	1.61	4.51E-01	1.08
A_51_P333923	Tspan1,9030418M05Ril	RIKEN cDNA 9030418M05 gene	7.27E-01	1.04	4.20E-04	1.62	5.66E-01	-1.11
A_51_P303160	Arg1,AI,PGIF,Arg-1,AI25	arginase 1, liver	3.99E-01	-1.05	6.00E-05	-2.10	2.43E-01	1.64
A_52_P382325	Ahnak,DY6,AA589382,/	RIKEN cDNA 2310047C17 gene	8.83E-01	1.02	3.35E-10	1.78	5.89E-01	1.04
A_52_P357593	Herc1,MGC7618,B2302	Hect (homologous to the E6-AP (UBE3A) carb	3.50E-04	1.87	1.00E+00	1.00	8.57E-02	1.37
A_52_P424692	Cd47,IAP,Itgp,AA40786	CD47 antigen (Rh-related antigen, integrin-as	0.00E+00	-2.34	5.45E-01	-1.15	1.48E-03	-1.21
A_52_P469939	2600014C01Rik,Gcc2,A	RIKEN cDNA 2600014C01 gene, mRNA (cDNA	3.93E-01	1.09	6.40E-06	3.70	4.23E-02	-1.43
A_51_P410845	Rnf148,Greul3,4933432	Ca2+-dependent activator protein for secreti	1.70E-04	1.84	1.00E+00	1.00	3.57E-01	-1.31
A_51_P183571	Serpine1,PAI1,PAI-1,Pla	serine (or cysteine) proteinase inhibitor, clad	6.30E-04	1.36	1.35E-11	1.61	6.58E-01	-1.03
A_52_P1042412	1700029I01Rik	Mus musculus RIKEN cDNA 1700029I01 gene	4.42E-01	1.03	4.00E-05	1.52	2.54E-01	-1.16
A_52_P164105	9530091C08Rik	RIKEN cDNA 9530091C08 gene (9530091C08	9.88E-11	1.64	2.58E-01	-1.89	4.48E-01	1.10
A_52_P199169	Rap1gds1,GDS1,BC011	RAP1, GTP-GDP dissociation stimulator 1 (Ra	1.76E-01	1.12	3.80E-04	1.85	5.57E-02	1.10
A_51_P380743	Lrpprc,LSFC,GP130,C76	Leucine-rich PPR-motif containing (Lrpprc), n	1.10E-04	2.11	1.00E+00	1.00	6.61E-01	-1.27
A_52_P914857	1600017P15Rik	Mus musculus adult female placenta cDNA, F	1.05E-23	1.53	5.19E-02	1.31	1.35E-01	1.22
A_52_P994399	Defcr4,Crp4	defensin related cryptdin 4	8.29E-01	-1.08	4.79E-08	3.19	9.84E-01	-1.00
A_52_P907763	AK036634	Mus musculus adult male bone cDNA, RIKEN	3.16E-13	1.60	7.34E-02	1.28	6.06E-03	1.88
A_52_P533030	Olfr438,MOR261-4	olfactory receptor MOR261-4	1.00E-05	-1.54	2.34E-02	-2.30	3.57E-01	1.42
A_51_P320137	Zxdc,MGC7160,BC0033	cDNA sequence BC003332	3.72E-17	-1.59	1.70E-03	-1.38	9.67E-02	-1.20
A_52_P309657	NAP028759-1	Unknown	1.49E-01	1.10	8.80E-04	1.57	7.66E-11	-1.25
A_52_P335768	AK035943	Mus musculus 16 days neonate cerebellum c	1.08E-09	1.81	3.69E-01	-1.20	1.20E-04	2.01
A_52_P298854	2600005C20Rik,Kiaa01	RIKEN cDNA 2600005C20 gene	2.78E-01	1.11	5.07E-10	1.51	8.37E-01	1.02
A_52_P172691	4732465E10Rik	MKIAA0716 protein	3.41E-16	1.53	9.59E-01	1.01	4.97E-01	1.10
A_52_P14158	Enah,Mena,WBP8,Ndp	Enabled homolog (Drosophila) (Enah), mRNA	6.13E-03	-1.22	4.66E-01	-1.51	8.00E-05	1.58
A_51_P456486	AK037870	16 days neonate thymus cDNA, RIKEN full-len	8.20E-08	-2.40	8.87E-01	-1.05	5.08E-01	1.15
A_51_P181031	Nalp2,NBS1,PAN1,PYPA	hypothetical protein E330007A02	3.46E-01	-1.38	1.23E-08	-1.52	8.95E-01	1.07
A_52_P1156578	Fars2,Fars1,2810431B2	Phenylalanine-tRNA synthetase 2 (mitochond	1.00E-04	1.67	1.11E-01	1.19	1.30E-07	1.85
A_52_P501007	Ugt2b5,m-1,Udpgt-3,AI	UDP-glucuronosyltransferase 2 family, memb	4.27E-01	-1.16	0.00E+00	-5.25	7.49E-01	1.04
A_52_P322612	Agpat1,1-AGP,1-AGPAT	1-acylglycerol-3-phosphate O-acyltransferase	7.89E-01	-1.01	2.14E-12	-1.59	9.44E-01	1.01
A_52_P686095	2410116I05Rik	RIKEN cDNA 2410116I05 gene	3.66E-01	1.15	2.72E-06	1.76	4.49E-01	1.08
A_52_P300451	Tcf23,Out,2010002O16	transcription factor 23	2.30E-01	1.10	4.65E-07	1.91	3.90E-06	1.49
A_51_P219109	Il12rb1,CD212,IL-12R[b	interleukin 12 receptor, beta 1	2.49E-03	1.20	2.49E-16	2.36	5.95E-01	1.07
A_51_P229759	2310075M15Rik,C8540	RIKEN cDNA 2310075M15 gene (2310075M1	3.10E-04	3.58	1.00E+00	1.00	1.00E+00	1.00
A_51_P512306	Kirrel2,NLG1,NEPH3,C3	kin of IRRE like 2 (Drosophila)	2.10E-01	-1.14	2.25E-06	1.81	9.24E-01	-1.01
A_52_P57317	7530404M11Rik,TAFA3	RIKEN cDNA 7530404M11 gene (7530404M1	7.48E-01	1.04	3.50E-04	1.86	2.80E-04	-1.53
A_52_P209328	Xpo5,Exp5,RanBp21,AI(	exportin 5	5.67E-01	1.04	3.40E-04	1.55	4.34E-01	-1.09
A_51_P167185	Lrp1b,LRP-DIT,9630004	Low density lipoprotein-related protein 1B (c	6.00E-05	1.54	1.02E-01	2.05	2.86E-01	-1.38
A_52_P875299	Rock1,Rock-I,1110055K	Rho-associated coiled-coil forming kinase 1 (	7.01E-02	1.22	6.97E-07	1.81	6.70E-01	-1.28
A_52_P923083	6230424H07Rik	PREDICTED: hypothetical protein LOC76138		6.00E-05	-1.62	1.30E-04	-1.54	1.55E-01	1.15
A_51_P310164	2810459M11Rik	RIKEN cDNA 2810459M11 gene, mRNA (cDN	2.42E-02	-1.10	1.16E-12	-1.51	9.18E-01	1.01
A_51_P461703	Mup1,Up-1,Ltn-1,Mup-	major urinary protein 1	4.71E-01	-1.08	1.42E-36	-36.20	2.97E-01	2.13
A_51_P189121	1810010G06Rik,KIAA07	RIKEN cDNA 1810010G06 gene, mRNA (cDNA	3.94E-01	-1.11	6.00E-05	1.72	1.13E-01	1.06
A_51_P181297	Serpinb1a,EI,EIA,LEI,PI2	serine (or cysteine) proteinase inhibitor, clad	1.61E-01	-1.13	3.71E-10	-1.58	1.60E-06	-1.39
A_51_P112682	Olfr159,mOR37e,Olfr37	olfactory receptor 159	2.40E-01	1.07	1.20E-07	-1.78	6.64E-01	1.04
A_52_P560113	H2-Bl,H2-B1	histocompatibility 2, blastocyst	1.50E-04	1.23	1.35E-24	1.63	1.28E-01	-1.13
A_51_P160713	Alb1,Alb-1,albumin	albumin 1	6.59E-01	1.07	1.67E-13	-6.26	6.39E-09	4.08
A_52_P884048	AK081536	PREDICTED: similar to mKIAA1616 protein [M	1.80E-08	2.78	1.00E+00	1.00	1.00E+00	1.00
A_51_P235821	Tsc22d3,DIP,Gilz,Dsip1,	glucocorticoid-induced leucine zipper	5.57E-01	1.04	6.88E-11	1.51	9.57E-01	1.00
A_51_P173107	1810046K07Rik	PREDICTED: hypothetical protein LOC69809		2.03E-03	-2.08	8.90E-01	1.05	6.00E-05	6.12
A_51_P251352	Slc25a13,Ctrn,AI78547!	solute carrier family 25 (mitochondrial carrie	1.40E-04	-1.17	1.52E-20	2.27	2.60E-01	-1.10
A_52_P241619	3110049J23Rik,Mawbp	RIKEN cDNA 3110049J23 gene	7.21E-01	1.04	4.43E-11	1.97	7.91E-01	1.03
A_52_P609024	2610036L11Rik	PREDICTED: hypothetical protein LOC66311		3.02E-01	1.11	1.30E-10	1.68	1.18E-01	-1.09
A_52_P517224	Trim63,MuRF1,Rnf28	PREDICTED: similar to tripartite motif-contair	5.21E-01	1.08	4.53E-06	2.33	4.32E-01	1.10
A_52_P421947	Mtss1,Mim,BC024131,I	Metastasis suppressor 1 (Mtss1), mRNA	6.13E-01	-1.03	1.64E-10	-1.56	1.27E-01	1.13
A_52_P691533	AB041803,MNCb-1768	CDNA sequence AB041803 (AB041803), mRN	2.53E-06	1.62	8.37E-01	1.03	7.34E-01	1.07
A_52_P262219	Fos,c-fos,D12Rfj1	FBJ osteosarcoma oncogene	9.70E-26	-1.71	1.18E-17	-3.22	7.39E-02	-1.54
A_52_P9425	TC997338	Unknown	7.07E-03	1.49	1.75E-06	2.63	3.86E-01	-1.12
A_52_P329105	1700009P03Rik,C8622!	RIKEN cDNA 1700009P03 gene	6.15E-10	1.58	1.26E-02	1.53	9.63E-01	1.01
A_52_P617817	Hspa4,70kDa,APG-2,Hs	heat shock protein 4	8.51E-01	1.01	1.80E-04	1.80	5.26E-02	-1.24
[illegible]	[illegible]	[illegible]	[illegible]	[illegible]	[illegible]	[illegible]	[illegible]	[illegible]

A_52_P1051923	BC049807	CDNA sequence BC049807 (BC049807), mRN	1.70E-10	1.59	1.00E+00	1.00	8.60E-01	-1.05
A_52_P972052	AK045847	PREDICTED: similar to mKIAA1616 protein [M	2.50E-04	1.52	9.78E-01	-1.01	4.43E-01	1.27
A_52_P384690	Tex11,BB024231,49305	testis expressed gene 11	4.20E-01	-1.08	1.22E-06	1.55	4.99E-01	1.08
A_52_P219266	1200004M23Rik,AU018	RIKEN cDNA 1200004M23 gene	3.76E-02	-1.11	8.05E-12	-1.60	1.56E-03	-1.13
A_52_P201892	Mnat1,P36,MAT1,E130	Menage a trois 1 (Mnat1), mRNA	5.36E-07	1.83	7.99E-01	-1.12	1.27E-01	1.92
A_52_P1187915	AI452102,B230337J15	PREDICTED: hypothetical protein XP_287711	4.50E-04	1.66	2.04E-02	1.77	6.11E-01	1.12
A_52_P615051	1200016E24Rik	Mus musculus adult male urinary bladder cD	4.22E-12	2.04	2.22E-09	2.10	3.00E-01	1.10
A_52_P834977	Pld1,Pld1a,Pld1b,C853	Phosphatidylcholine-specific phospholipase D	1.34E-02	1.35	2.60E-04	1.81	5.43E-01	1.10
A_51_P340060	Setbp1,Seb,MGC90748,	SET binding protein 1 (Setbp1), mRNA	1.81E-27	1.89	5.99E-01	1.28	2.04E-01	1.57
A_51_P183723	9330132E09Rik	PREDICTED: RIKEN cDNA 9330132E09 gene [	3.04E-01	1.06	1.20E-04	-1.92	7.53E-01	1.02
