# Supplementary material for: Gene expression profiling in brain of mice exposed to the marine neurotoxin ciguatoxin reveals an acute anti-inflammatory, neuroprotective response
Source: BMC Neurosci. 2010 Aug 26;11:107. doi: 10.1186/1471-2202-11-107 (PMC2939656; doi:10.1186/1471-2202-11-107)
Supplement: Additional file 2 — PCR primers. Accession numbers for genes along with PCR primers and annealing temperatures used for RT-PCR validation of microarray data. [file 1471-2202-11-107-S2.PDF]

Additional file 2. PCR primers

Gene Name	Accession #	Forward Primer	Reverse Primer	Annealing Temp
bHLH-PAS nxf	NM_153553.1	TTGCCCAGGGCGACAGTATCTA	GTGAGCATGGAATCGACCTCGA	58°C
Cirbp	NM_007705	CGAGGCTTTGGGTTTGTCACCT	CCGGTTGTCAGAAGACTTGCCA	58°C
Cxcl7	NM_023785	GCTTCATCTGTGCCAAACCATT	GCCCGTCTTCATCATTGTTTGT	60°C
Egr1	NM_007913	TGCCACCACCTCATTCCCAT	GGTGCTGAAGGAGCTGCTGA	58°C
Egr4	NM_020596	GCTTCTCTGCAGTAGGGACCAA	ACCTGGGGCCAACGAGAA	55°C
Fos	NM_010234	CTGGAGTTTATTTTGGCAGCCC	AGGAATTGCTGTGCAGAGGCT	62°C
Hp	NM_017370.1	GTGGTGGATATCGGGCTAATCA	CGGCCTGGTGCTATGTAGTCTT	60°C
Ier2	NM_010499	CTGTGGGTGAGCCTGAACTGAA	CCGTGGGAAAAGTAAACAGACT	58°C
Il1b	NM_008361	CCTTCCAGGATGAGGACATGA	CCTTCCAGGATGAGGACATGA	58°C
Infi47	NM_008330	GCTCTTGCCCAATATCTCGGAT	CGATTGATCATCCAAGCCAAAA	58°C
JunB	NM_008416	ACAGCTGGGTTTGAGTCGTGGC	TCTTCACCTTGTCCTCCAGGCG	62°C
Map3k6	NM_016693	GCGTGAGAGCTTCAGCATGAC	GCTGCCAACACAATCCGAGTTC	62°C
NF-KappaB	NM_010907	CGTTCCTGCACTTGGCAATCA	GCCAGCTTTCAGAAGTGCCTCA	58°C
Plac8	NM_139198	GAAGATGGCTCAGGCACCAACA	TCCGCAGTCACTGAAGCAATCA	58°C
Rbm3	NM_016809	GGAGGTCCACTGCAAAGCTTTA	CCTGAGTTTTGGAGGCTGGAGT	55°C
s100a8	NM_013650	TCGAGGAGTTCCTTGCGATGG	CATATCCAGGGACCCAGCCCTA	55°C
s100a9	NM_009114	TGGCCAACAAAGCACCTTCTCA	TGCCAACTGTGCTTCCACCATT	62°C
scarb2	NM_007644.2	CGGTAGACCAGACGATCGAGAA	CCTTGGAGGATCTCCTCAGGAT	55°C
Sgk	NM_011361	GAGAAGGATGGGCCTGAACGAT	CGGACCCAGGTTGATTTGTTGA	60°C
Sgk3	AK077817	CGTATGCTGTGTATGCGCGTGT	CGATCTGAGCGGGGTTTACA	55°C
Tgtp	NM_011579	TCCAGAAGATCACCATGGCAAA	CCGATGTCCCTGTTTCCAAAAT	58°C
Tmem59	NM_029565.2	CTTGCACACCTACCCGAAGGA	GCTTCTGTGCACGCAGATTCA	55°C
